# Home-Based Exercise and Self-Management After Lung Cancer Resection: A Randomized Clinical Trial

**DOI:** 10.1001/jamanetworkopen.2024.47325

**Published:** 2024-12-02

**Authors:** Catherine L. Granger, Lara Edbrooke, Phillip Antippa, Gavin Wright, Christine F. McDonald, Diana Zannino, Shaza Abo, Meinir Krishnasamy, Louis Irving, Karen E. Lamb, Georgina Whish-Wilson, Linda Denehy, Selina M. Parry

**Affiliations:** 1 Department of Physiotherapy, The University of Melbourne, Parkville, Victoria, Australia; 2 Department of Physiotherapy, The Royal Melbourne Hospital, Parkville, Victoria, Australia; 3 Department of Health Services Research, Peter MacCallum Cancer Centre, Melbourne, Victoria, Australia; 4 Department of Cardiothoracic Surgery, The Royal Melbourne Hospital, Parkville, Victoria, Australia; 5 Department of Cardiothoracic Surgery, St Vincent’s Hospital Melbourne, Fitzroy, Victoria, Australia; 6 Research and Education Lead Program, Victorian Comprehensive Cancer Centre, Melbourne, Victoria, Australia; 7 Institute for Breathing and Sleep, Heidelberg, Victoria, Australia; 8 Department of Respiratory and Sleep Medicine, Austin Hospital, Heidelberg, Victoria, Australia; 9 Centre for Epidemiology and Biostatistics, Melbourne School of Population and Global Health, The University of Melbourne, Parkville, Victoria, Australia; 10 MISCH (Methods and Implementation Support for Clinical Health) Research Hub, Faculty of Medicine, Dentistry and Health Sciences, The University of Melbourne, Parkville, Victoria, Australia; 11 Department of Nursing, The University of Melbourne, Parkville, Victoria, Australia; 12 Department of Respiratory and Sleep Medicine, Royal Melbourne Hospital, Parkville, Victoria, Australia

## Abstract

**Importance:**

Patients with lung cancer have poor physical functioning and quality of life. Despite promising outcomes for those who undertake exercise programs, implementation into practice of previously tested hospital-based programs is rare.

**Objective:**

To evaluate a home-based exercise and self-management program for patients after lung resection.

**Design, Setting, and Participants:**

A randomized clinical trial with assessor blinding was conducted among 116 patients undergoing surgery for non–small cell lung cancer from November 23, 2017, to July 31, 2023, at tertiary hospitals in Australia. Patients were followed up for 12 months postoperatively.

**Intervention:**

Patients randomized to the intervention group received a postoperative 3-month home-based exercise and self-management program, supported by weekly physiotherapist-led telephone consultations. Patients randomized to the control group received usual care.

**Main Outcomes and Measures:**

The primary outcome was self-reported physical function (30-item European Organization for the Research and Treatment of Cancer Core Quality of Life Questionnaire [EORTC QLQ-C30] score) at 3 months. Secondary outcomes included objective measures of physical function and exercise capacity (at 3 and 6 months) and patient-reported outcomes including quality of life (at 3 and 6 months, with some questionnaires completed at 12 months). Analysis was performed on an intent-to-treat basis.

**Results:**

A total of 1370 patients were screened, with 177 eligible and 116 consented (mean [SD] age, 66.4 [9.6] years; 68 women [58.6%]). Of these 116 patients, 58 were randomized to the intervention and 58 to the control. A total of 103 patients (88.8%) completed assessments at 3 months, 95 (81.9%) at 6 months, and 95 (81.9%) at 12 months. There were no statistically significant differences between the intervention and control groups for self-reported physical function (EORTC QLQ-C30 physical functioning domain score) at the 3-month primary end point (mean [SD] score, 77.3 [20.9] vs 76.3 [18.8]; mean difference, 1.0 point [95% CI, −6.0 to 8.0 points]). Patients in the intervention group, compared with the control group, had significantly greater exercise capacity (6-minute walk distance: mean difference, 39.7 m [95% CI, 6.8-72.6 m]), global quality of life (mean difference, 7.1 points [95% CI, 0.4-13.8 points]), and exercise self-efficacy (mean difference, 16.0 points [95% CI, 7.0-24.9 points]) at 3 months as well as greater objectively measured physical function (Short Physical Performance Battery score: mean difference, 0.8 points [95% CI, 0.1-1.6 points]), exercise capacity (6-minute walk distance: mean difference, 50.9 m [95% CI, 6.7-95.1 m]), and exercise self-efficacy (mean difference, 10.1 points [95% CI, 1.9-18.2 points]) at 6 months. One minor adverse event and no serious adverse events occurred.

**Conclusions and Relevance:**

In this randomized clinical trial, a postoperative home-based exercise and self-management program did not improve self-reported physical function in patients with lung cancer. However, it did improve other important clinical outcomes. Implementation of this program into lung cancer care should be considered.

**Trial Registration:**

http://anzctr.org.au Identifier: ACTRN12617001283369

## Key Points

**Question:**

Can a 3-month home-based exercise and self-management program improve physical function after lung resection?

**Findings:**

In this randomized clinical trial of 116 patients, there were not statistically significant improvements in self-reported physical function in the intervention group compared with the control group (mean difference physical function domain of 1.0 point). There were statistically and clinically significant improvements in several secondary end points, including quality of life, exercise capacity, and self-efficacy.

**Meaning:**

The findings suggest that although a home-based exercise and self-management program did not improve physical function, it improves some important clinical outcomes in patients with lung cancer and that several benefits are maintained beyond the conclusion of the structured exercise program.

## Teaser

This randomized clinical trial evaluates whether a home-based exercise and self-management program can improve physical function among patients after lung resection.

## Introduction

Lung cancer is the most common cancer diagnosed globally.^1^ Up to 50% of individuals with non–small cell lung cancer (NSCLC) receive a diagnosis of early-stage disease (stage I-IIIA), and the treatment aim is curative, involving surgery and neoadjuvant or adjuvant therapies. Long-term deficits in physical functioning affecting health-related quality of life (HRQOL) and increasing disease burden are being increasingly documented among patients with lung cancer.^2,3,4,5,6^

Evidence for the benefits of exercise among patients with lung cancer is documented across the continuum of surgical lung cancer care from before surgery^7^ to 12 months after surgery.^8,9,10^ The postoperative exercise programs for lung cancer tested to date were typically conducted in a supervised hospital setting,^8,9,10^ and, despite promising outcomes, implementation into clinical practice has been rare worldwide.^11,12^ Qualitative data have demonstrated barriers to implementation at patient, clinician, and institutional levels, affecting translation of research into practice.^13,14^

As a result of increased lung cancer screening, early detection, and advances in lung cancer treatments, the number of lung cancer survivors will continue to increase. The lack of implementation of hospital-based exercise programs, as described, indicates an urgent need to examine novel programs that may mitigate long-term functional deficits and poor HRQOL. Unsupervised home exercise programs are an alternative model that may be less resource intensive, may increase accessibility for people living in rural areas, and may empower patients through the increased flexibility associated with choosing how and when to incorporate exercise in their daily schedule.^10,13,15^

There has been limited testing of unsupervised and remotely supported exercise programs for patients after lung cancer surgical procedures.^8,10,16^ Initial feasibility (phase 1) results showed promise for the role of an unsupervised remote program incorporating behavior change, physical activity, and strengthening.^17^ If effective in phase 2, this program has the potential to be implemented at low cost as a sustainable and scalable model of care.

The purpose of this randomized clinical trial was to examine whether a postoperative exercise and self-management program, delivered in the home setting and supported by physiotherapist-led telephone consultations and self-management strategies, was associated with improved self-reported physical function compared with usual postacute care for patients with lung cancer.

## Methods

### Study Design and Setting

This was a 2-arm parallel-group randomized clinical trial approved by the Human Research Ethics Committee at Melbourne Health. All participants provided written informed consent. Methods are described in full elsewhere.^18^ Supplement 1 includes the final trial protocol, list of amendments, and statistical analysis plan. The trial is reported according to the Consolidated Standards of Reporting Trials (CONSORT) reporting guideline statement^19^ with Non-Pharmacologic Trial and Patient-Reported Outcomes extensions,^20,21^ the Guidelines for Reporting Trial Protocols and Completed Trials Modified Due to the COVID-19 Pandemic and Other Extenuating Circumstances (CONSERVE-CONSORT) extension,^22^ and the Template for Intervention Description and Replication (TIDieR) Checklist.^23^ The trial was designed by the investigators, coordinated from The University of Melbourne, and undertaken at the lung cancer services of tertiary hospitals Royal Melbourne Hospital, St Vincent’s Hospital (Melbourne), and Austin Hospital in Melbourne, Australia.

### Study Population

The target for the intervention was patients who received lung resection for newly diagnosed NSCLC. All patients presenting to the lung cancer services were eligible for inclusion if their treatment plan was to include surgery for suspected or confirmed NSCLC, they had a life expectancy of greater than 6 months, their Eastern Cooperative Oncology Group performance status was 0 to 2, and they were not already meeting the aerobic physical activity recommendations^24^ (eMethods in Supplement 2). Data collection with follow-up to 12 months began November 23, 2017, and ended on July 31, 2023.

### Randomization

**Figure 1.**
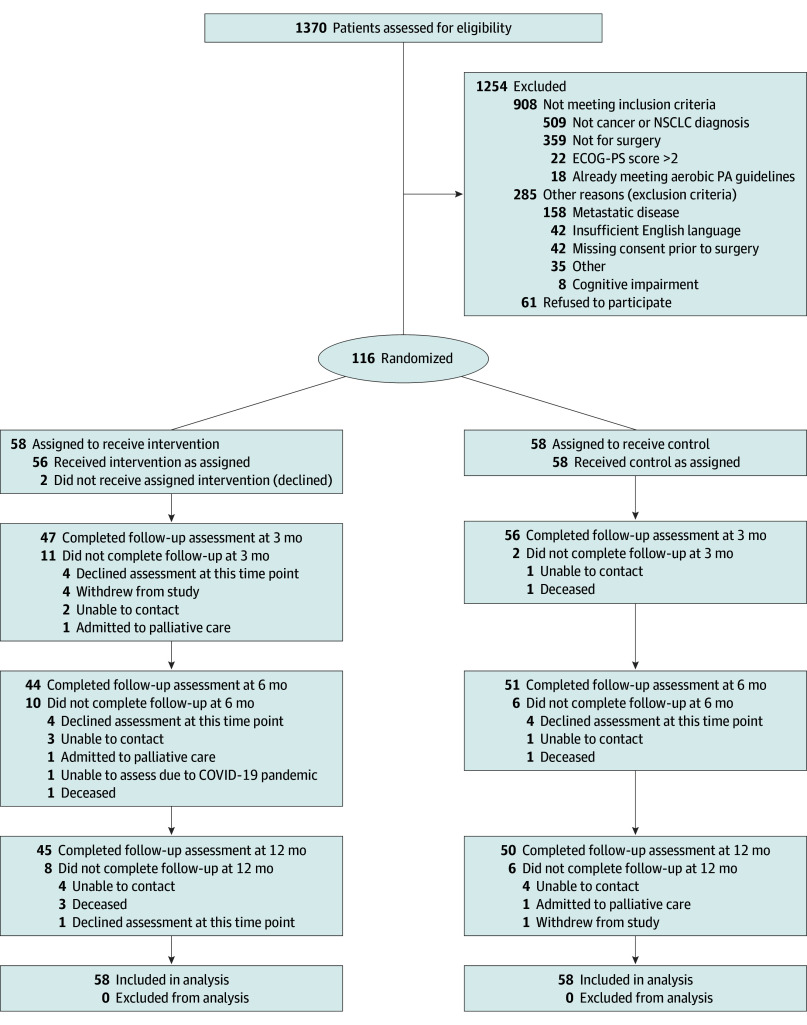
CONSORT Flow Diagram ECOG-PS indicates Eastern Cooperative Oncology Group performance status scale; NSCLC, non–small cell lung cancer; and PA, physical activity.

Patients were randomly allocated (1:1 allocation) to either the intervention or control group using a computer-generated schedule of randomly permuted blocks, stratified by site, created by an independent statistician. Allocation was conducted by the primary investigators (C.L.G. and S.M.P.) or the trial coordinator (S.A.). Randomization and group allocation occurred after the patient had consented and undergone baseline (preoperative) testing and surgery (Figure 1).

### Usual Care

Patients in the control group received usual medical, nursing, and allied health care in the perioperative period. For physiotherapy, this was early mobilization and monitoring for postoperative pulmonary complications.^11,25^ Prehabilitation or outpatient exercise programs were not provided before or after surgery. Deviations to this practice were documented, for example, if a patient was referred to outpatient pulmonary rehabilitation after surgery.

### Intervention

The intervention was a 3-month, physiotherapy-led, home-based exercise and self-management program delivered remotely. Participants completed the initial session in person prior to hospital discharge and then were supported by the physiotherapist through weekly telephone consultations for a target of 12 sessions. In the first session, participants were provided with a home exercise booklet including exercises to be performed (aerobic and strengthening), a diary to track daily program compliance, education on managing symptoms with exercise, and a physical activity monitor (to self-monitor steps at home). The physiotherapist identified readiness to engage with the exercise program and used behavior change techniques to identify potential barriers and facilitators to program participation. The exercise program was tailored depending on the patient’s functional level, symptoms, and goals. Physiotherapists received standardized training including in behavior change techniques in line with the intervention protocol.

### Data Collection and Follow-Up

Data on clinical characteristics and race and ethnicity (classified as Asian, Black, or White), determined by patients themselves (self-report) and reported to hospital staff, were collected through electronic health records. Race and ethnicity were assessed in the study to report the characteristics of the sample. Patient demographic characteristics were collected via self-administered questionnaires. Most outcomes were assessed at baseline (preoperative) and at 3 and 6 months postoperatively. The primary outcome was also assessed 12 months postoperatively. Blinded assessors were registered physiotherapists, trained by a primary investigator (S.M.P.) using a standard operating procedure developed for the trial, and their compliance with the protocol was audited every 6 months. Assessments occurred in the hospital outpatient departments or patient’s home depending on patient preference. Questionnaires were administered primarily in paper format or over the telephone or in person for participants unable to read or write.

### Outcomes

The primary outcome was physical function at 3 months postoperatively assessed using the physical functioning scale of the 30-item European Organization for the Research and Treatment of Cancer Core Quality of Life Questionnaire (EORTC QLQ-C30)^26^ (also assessed at 6 and 12 months). Scores ranged from 0 to 100, with higher scores indicating better function. Secondary outcomes assessed at 3 and 6 months included objectively measured physical function (Short Physical Performance Battery [overall score: 0 to 12 points, with a lower score indicating worse performance; subscore items: 0 to 4 points, with a lower score indicating worse performance]),^27^ functional exercise capacity (6-minute walk test),^28^ quadriceps and hand-grip muscle strength (dynamometry),^29^ and patient-reported outcomes, including physical activity levels,^30^ exercise self-efficacy,^31,32^ fatigue,^33^ sleep quality,^34^ distress,^35^ and financial toxicity.^36^ The EORTC QLQ-C30 with lung cancer subscale assessed HRQOL and symptoms over 12 months^26,37^ (eMethods in Supplement 2).

Adverse events were defined a priori and were reported as the number of participants experiencing a serious or minor adverse event across both study groups, with intervention participants asked to recall in every telephone consultation whether an adverse event was experienced in relation to intervention enactment.^18^ Physiotherapist adherence to the protocol was measured by random audits of telephone consultation recordings and intervention session documentation by investigators (C.L.G. and S.M.P.) to ensure the intervention delivery was undertaken as intended.

### COVID-19 Pandemic Modifications

The trial was under way at the start of the COVID-19 pandemic. Small modifications were made to the protocol to allow the trial to be completed within local hospital and government restrictions (see amendments to the trial protocol in Supplement 1). These modifications included allowing patients to complete follow-up testing remotely and pauses to recruitment. The third recruitment site did not open for recruitment, and site numbers were unbalanced (eMethods in Supplement 2).

### Statistical Analysis

Analysis was performed on an intent-to-treat basis. A detailed plan was developed prior to database lock (statistical analysis plan in Supplement 1). Additional details are provided in eMethods in Supplement 2. A total of 112 patients was needed to detect a minimal clinically important difference (MCID) of 12.9 units in the mean EORTC-QLQ-C30 physical function score^38^ (primary outcome) between groups at 3-month follow-up (primary time point), assuming an SD of 21.3 units,^17^ with 20% attrition (80% power; 2-sided α, 5%).

Participants were analyzed according to randomized study group. Continuous primary and secondary outcomes that had a preoperative assessment (baseline) and at least 1 postoperative time point (3, 6, and/or 12 months) were analyzed using a likelihood-based longitudinal data analysis model incorporating all available outcome data as the response. This modeling approach assumes missing outcomes data are missing at random. Each model included group allocation, time point, a group-by-time point interaction, and recruitment site. Differences in mean change in outcome from baseline between groups were estimated at each postoperative time point and reported with their corresponding 95% CI. The complier mean causal effect was estimated in secondary analyses of the primary outcome, using collected adherence data. Heterogeneity of the intervention effect according to postoperative cancer treatment (yes or no) for the primary outcome was assessed by including interactions between postoperative treatment, study group, and time. Analyses were performed in Stata/SE, version 17.0 (Stata Corp).

## Results

### Study Participants

From November 23, 2017, to July 11, 2022, 116 patients were enrolled from 1370 screened (Figure 1). The main reason patients did not meet the inclusion criteria was not having an NSCLC diagnosis (509 of 1254 patients excluded [40.6%]). Sixty-one patients declined participation. From December 11, 2017, to July 18, 2022, 116 patients (mean [SD] age, 66.4 [9.6] years; 68 women [58.6%] and 48 men [41.4%]; 11 Asian patients [9.5%], 2 Black patients [1.7%], and 103 White patients [88.8%]) were randomized postoperatively (Table 1), resulting in 58 patients in each study group (Figure 1). Patient demographic and clinical characteristics at baseline were similar between groups, with slight imbalances for performance status, histologic type, postoperative therapy, smoking status, and occupational status (Table 1).

**Table 1.** Patient Demographic and Clinical Characteristics at Baseline

Measurement variable	No. (%)		
	Control (n = 58)	Intervention (n = 58)	Total (N = 116)
Age, mean (SD), y	67.5 (8.1)	65.4 (10.8)	66.4 (9.6)
Sex			
Female	35 (60.3)	33 (56.9)	68 (58.6)
Male	23 (39.7)	25 (43.1)	48 (41.4)
Race and ethnicity			
Asian	5 (8.6)	6 (10.3)	11 (9.5)
Black	2 (3.4)	0	2 (1.7)
White	51 (87.9)	52 (89.7)	103 (88.8)
BMI, mean (SD)	27.8 (7.0)	27.1 (5.3)	27.5 (6.2)
Smoking status			
Never smoked	8 (13.8)	14 (24.1)	22 (19.0)
Quit smoking >8 wk ago	38 (65.5)	28 (48.3)	66 (56.9)
Current smoker	12 (20.7)	16 (27.6)	28 (24.1)
Lung cancer histologic type			
Squamous	13 (22.4)	7 (12.1)	20 (17.2)
Adenocarcinoma	38 (65.5)	46 (79.3)	84 (72.4)
Other	7 (12.1)	5 (8.6)	12 (10.3)
Cancer stage			
IA or IB	28 (50.0)	30 (52.6)	58 (51.3)
IIA or IIB	16 (28.6)	12 (21.1)	28 (24.8)
IIIA or IIIB	8 (14.3)	11 (19.3)	19 (16.8)
IV	4 (7.1)	4 (7.0)	8 (7.1)
Comorbidities			
COPD	20 (34.5)	21 (36.2)	41 (35.3)
Tobacco consumption	50 (86.2)	46 (79.3)	96 (82.8)
Diabetes	11 (19.0)	6 (10.3)	17 (14.7)
Kidney insufficiency	1 (1.7)	3 (5.3)	4 (3.5)
Respiratory	27 (46.6)	26 (44.8)	53 (45.7)
Cardiovascular	37 (63.8)	36 (63.2)	73 (63.5)
Neoplastic	23 (40.4)	21 (36.2)	44 (38.3)
Alcoholism	4 (7.0)	5 (8.9)	9 (8.0)
Colinet comorbidity score, mean (SD)	8.6 (3.6)	7.8 (3.5)	8.2 (3.5)
ECOG-PS doctor rated (preoperative)			
0	47 (82.4)	46 (86.8)	93 (84.6)
1	7 (12.3)	6 (11.3)	13 (11.8)
2	3 (5.3)	1 (1.9)	4 (3.6)
Highest educational level			
No formal schooling	2 (3.6)	0	2 (1.8)
Primary schooling	4 (7.1)	2 (3.6)	6 (5.4)
Secondary or high school	29 (51.8)	30 (53.6)	59 (52.7)
Trade or community college	9 (16.1)	12 (21.4)	21 (18.8)
University degree	7 (12.5)	9 (16.1)	16 (14.3)
Other	5 (8.9)	3 (5.3)	8 (7.1)
Occupational status			
Working full time (≥35 h/wk)	4 (7.1)	14 (25.9)	18 (16.4)
Working part time (<35 h/wk)	11 (19.6)	5 (9.3)	16 (14.6)
Retired	32 (57.1)	22 (40.7)	54 (49.1)
Home duties, carer, or volunteer	2 (3.6)	2 (3.7)	4 (3.6)
Sick leave or receiving disability payments	3 (5.4)	9 (16.7)	12 (10.9)
Unemployed	3 (5.4)	2 (3.7)	5 (4.5)
Other	1 (1.8)	0	1 (0.9)
Living arrangement			
Home alone, independent	14 (24.2)	13 (22.4)	27 (23.3)
Home with family	41 (70.7)	35 (60.3)	76 (65.5)
Home with supports	2 (3.4)	6 (10.3)	8 (6.9)
Retirement community	1 (1.7)	1 (1.7)	2 (1.7)
Other	0	3 (5.2)	3 (2.6)
Respiratory function, mean (SD)			
FVC, L	3.2 (0.8)	3.3 (0.9)	3.3 (0.9)
FVC, % predicted	102.2 (18.4)	106.1 (19.5)	104.2 (18.9)
FEV_1_, L	2.1 (0.6)	2.3 (0.7)	2.2 (0.7)
FEV_1_, % predicted	85.3 (19.8)	93.2 (20.8)	89.2 (20.6)
FEV_1_ to FVC ratio	72.5 (15.2)	77.1 (14.6)	74.8 (15.0)
DLCO, mL/mm Hg/min	17.5 (6.0)	17.8 (5.9)	17.6 (5.9)
DLCO, % predicted	71.7 (19.4)	76.3 (16.9)	74.0 (18.2)
Surgical and hospital details			
Type of surgery			
Lobectomy, lower or upper	35 (60.3)	39 (67.2)	74 (63.8)
Segmentectomy, lower or upper	3 (5.2)	5 (8.6)	8 (6.9)
Wedge resection, lower, middle or upper	10 (17.2)	9 (15.6)	19 (16.4)
Pneumonectomy	2 (3.5)	1 (1.7)	3 (2.6)
Other (including combination)	8 (13.8)	4 (6.9)	12 (10.3)
Surgical approach			
Thoracotomy, standard posterolateral	4 (6.9)	5 (8.6)	9 (7.8)
Thoracotomy, muscle sparing	3 (5.2)	4 (6.9)	7 (6.0)
VATS	51 (87.9)	49 (84.5)	100 (86.2)
Hospital length of stay, median (IQR), d	5.0 (4.0-8.0)	5.0 (3.0-9.0)	5.0 (3.0-8.0)
Postoperative complication	25 (43.8)	28 (48.3)	53 (46.1)
Critical care admission after surgery	1 (1.7)	1 (1.7)	2 (1.7)
Discharge destination			
Home	56 (100.0)	56 (96.6)	112 (98.2)
Inpatient rehabilitation	0	1 (1.7)	1 (0.9)
Other (deceased during admission)	0	1 (1.7)	1 (0.9)
Referred to prehabilitation before surgery	0	0	0
Referred to pulmonary rehabilitation after discharge	7 (12.1)	7 (12.1)	14 (12.1)
Postoperative cancer treatment			
No chemotherapy or RT	31 (53.4)	37 (63.8)	68 (58.6)
Postoperative chemotherapy	18 (31.0)	15 (25.9)	33 (28.4)
Postoperative stereotactic radical RT	3 (5.2)	0	3 (2.6)
Postoperative radical chemotherapy and RT	0	0	0
Postoperative palliative chemotherapy	0	0	0
Postoperative palliative RT	0	1 (1.7)	1 (0.9)
Other	13 (22.4)	9 (15.5)	22 (18.9)

Abbreviations: BMI, body mass index (calculated as weight in kilograms divided by height in meters squared); COPD, chronic obstructive pulmonary disease; DLCO, diffusing capacity of the lungs for carbon monoxide; ECOG-PS, Eastern Cooperative Oncology Group performance status scale; FEV_1_, forced expiratory volume in 1 second; FVC, forced vital capacity; RT, radiotherapy; VATS, video-assisted thoracoscopic surgery.

Two patients from the 58 allocated to the intervention group (3.4%) did not participate in the first intervention session or any sessions thereafter (reasons: declined [felt too unwell]; declined [burden of commitment]). Based on the a priori definition of intervention adherence (participation in ≥8 of a target of 12 sessions), 46 patients (79.3%) allocated to the intervention were considered adherent. There was no crossover of patients between groups.

Assessors were unblinded on 13 of 293 testing occasions (4.4%); 5 times due to staffing shortages related to the COVID-19 pandemic, 3 times due to staffing shortages for other reasons, and 5 times due to patients specifically telling assessors about participation in the intervention. A total of 103 patients (88.8%) completed follow-up assessment at 3 months (47 intervention; 56 control), and 95 patients (81.9%) completed assessments at 6 and 12 months only (Figure 1). Fourteen patients (12.1%; 7 in each group) were referred to pulmonary rehabilitation (Table 1).

No serious adverse events were reported. One minor adverse event was reported in the intervention group (new calf pain), which resolved spontaneously.

### Primary Outcome

There was no statistically significant between-group difference in self-reported physical function at 3 months, with a mean (SD) EORTC QLQ-C30 physical functioning scale score of 77.3 (20.9) in the intervention group and 76.3 (18.8) in the control group (mean difference, 1.0 [95% CI, −6.0 to 8.0]; *P* = .78) (eTable 1 in Supplement 2). Analysis of the complier mean causal effect showed no statistically significant difference at 3 months (0.7 [95% CI, −7.0 to 8.3]; *P* = .86). Although the direction of the estimated effect differed based on receipt of postoperative adjuvant treatment, 95% CIs for each group included the null. There were no statistically significant between-group differences in self-reported physical function at 6 or 12 months (eTable 2 in Supplement 2).

### Secondary Outcomes

**Figure 2.**
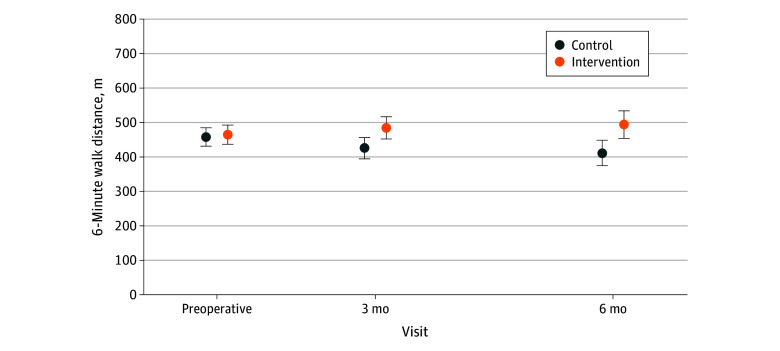
Mean Functional Exercise Capacity (6-Minute Walk Distance) Over Time Mean 6-minute walk distance in meters at baseline and 3 and 6 months’ follow-up in both intervention and control groups. Error bars indicate 95% CIs.

Statistically significant differences were detected between the intervention and control groups for many continuous secondary outcomes. The intervention showed a positive effect compared with control for objectively measured physical function at 6 months (mean [SD] Short Physical Performance Battery overall score: 11.2 [1.2] vs 9.8 [2.3] points; mean difference, 0.8 points [95% CI, 0.1-1.6 points]; mean [SD] gait subscore: 3.9 [0.3] vs 3.5 [0.7] points; mean difference, 0.4 points [95% CI, 0.1-0.6 points]; and mean [SD] balance subscore: 3.9 [0.4] vs 3.4 [0.9] points; mean difference, 0.4 points [95% CI, 0.1-0.7 points]), but the 95% CIs included zero at 3 months (Table 2; eFigure in Supplement 2). Higher exercise capacity was found for the intervention group compared with the control group at both 3 and 6 months (mean [SD] 6-minute walk at 3 months: 484.6 [97.0] vs 425.7 [100.6] m; mean difference, 39.7 m [95% CI, 6.8-72.6 m]; mean [SD] 6-minute walk at 6 months: 494.0 [114.7] vs 412.0 [116.7] m; mean difference, 50.9 m [95% CI, 6.7-95.1 m]) (Table 2; Figure 2). No statistically significant between-group differences were observed in muscle strength (Table 2).

**Table 2.** Summary of Continuous Secondary Outcomes (Objectively Measured Physical Function, Exercise Capacity, and Strength) With Measures at Baseline

Test	Mean (SD)		Mean difference (95% CI)^a^
	Control group	Intervention group	
SPPB overall score^b^			
Baseline	10.3 (1.6)	10.2 (2.3)	NA
At 3 mo	9.9 (2.1)	10.9 (1.8)	0.7 (1.4 to −0.1)
At 6 mo	9.8 (2.3)	11.2 (1.2)	0.8 (0.1 to 1.6)
SPPB gait subscore^c^			
Baseline	3.8 (0.4)	3.8 (0.7)	NA
At 3 mo	3.7 (0.8)	3.9 (0.5)	0.2 (0.5 to −0.0)
At 6 mo	3.5 (0.7)	3.9 (0.3)	0.4 (0.1 to 0.6)
SPPB balance subscore^c^			
Baseline	3.5 (0.9)	3.4 (1.0)	NA
At 3 mo	3.5 (0.9)	3.8 (0.5)	0.2 (0.6 to −0.1)
At 6 mo	3.4 (0.9)	3.9 (0.4)	0.4 (0.1 to 0.7)
SPPB chair rise subscore^c^			
Baseline	2.9 (1.1)	3.0 (1.3)	NA
At 3 mo	2.8 (1.3)	3.2 (1.2)	0.3 (0.7 to −0.2)
At 6 mo	2.9 (1.3)	3.4 (1.0)	0.1 (0.5 to −0.3)
6-min Walk distance, m			
Baseline	457.9 (98.5)	464.9 (102.3)	NA
At 3 mo	425.7 (100.6)	484.6 (97.0)	39.7 (6.8 to 72.6)
At 6 mo	412.0 (116.7)	494.0 (114.7)	50.9 (6.7 to 95.1)
Quadriceps highest peak force, kg^d^			
Baseline	21.4 (6.8)	22.2 (7.4)	NA
At 3 mo	22.6 (6.3)	22.3 (6.5)	−0.9 (1.5 to −3.3)
At 6 mo	20.7 (6.8)	22.2 (6.2)	0.1 (2.5 to −2.4)
Quadriceps longest time to peak force, s^d^			
Baseline	4.4 (1.6)	4.3 (1.5)	NA
At 3 mo	4.3 (1.4)	3.8 (1.7)	−0.5 (0.2 to −1.2)
At 6 mo	4.2 (1.3)	4.0 (1.7)	−0.2 (0.6 to −0.9)
Hand grip highest peak force, kg^d^			
Baseline	29.0 (8.7)	30.9 (10.0)	NA
At 3 mo	27.6 (9.7)	31.9 (9.7)	1.1 (3.4 to −1.2)
At 6 mo	27.1 (9.3)	30.8 (9.3)	−0.3 (2.2 to −2.8)

Abbreviations: NA, not applicable; SPPB, Short Physical Performance Battery.

^a^ The model estimates the mean difference between intervention and control from a common baseline so that any change over time between the groups is not due to any difference in baseline scores. Differences use the control group as the reference group.

^b^ SPPB overall score range: 0 to 12 points, with a lower score indicating worse performance.

^c^ SPPB subscore item range: 0 to 4 points, with a lower score indicating worse performance.

^d^ Data presented for right-side measurements.

In the intervention group preoperatively, 42.1% of patients (24 of 57) were engaged in moderate or high levels of physical activity (based on International Physical Activity Questionnaire [IPAQ] categorization), reaching 65.9% (29 of 44) by 6 months (eTable 3 in Supplement 2). In the control group preoperatively, 58.6% of patients (34 of 58) were engaged in moderate or high levels of physical activity, with similar levels at 6 months (56.0% [28 of 50]). At 3 months, there was a relative increase in the odds of moderate or high physical activity in the intervention group compared with the control (odds ratio, 1.21 [95% CI, 0.42-3.50]); at 6 months, this was a relative increase (odds ratio, 1.76 [95% CI, 0.58-5.30]), but no statistically significant differences were detected in IPAQ total metabolic equivalent of task (MET) minutes per week (Table 3). The intervention was superior to control for self-efficacy for exercise (related to barriers to exercise) at both 3 months (mean difference, 16.0 points [95% CI, 7.0-24.9 points]) and 6 months (mean difference, 10.1 points [95% CI, 1.9-18.2 points]), but there were no statistically significant differences in task or walking self-efficacy.

**Table 3.** Summary of Continuous Secondary Outcomes (Self-Reported Physical Activity Levels and Self-Efficacy for Exercise) With Measures at Baseline

Questionnaire	Mean (SD)		Mean difference (95% CI)^a^
	Control group	Intervention group	
IPAQ-SF total MET, min/wk			
Baseline	768.5 (769.4)	990.5 (1462.2)	NA
At 3 mo	904.0 (1090.7)	1765.0 (2326.9)	435.4 (1018.2 to −147.4)
At 6 mo	1097.6 (1830.0)	1384.1 (1283.9)	172.7 (833.6 to −488.1)
Barrier self-efficacy overall score, %^b^			
Baseline	40.6 (23.4)	38.3 (26.3)	NA
At 3 mo	29.2 (24.3)	44.6 (25.2)	16.0 (7.0 to 24.9)
At 6 mo	32.9 (21.7)	42.2 (24.8)	10.1 (1.9 to 18.2)
Task self-efficacy overall score, %^b^			
Baseline	31.0 (27.9)	39.8 (28.5)	NA
At 3 mo	22.1 (24.0)	33.7 (28.0)	8.2 (16.8 to −0.4)
At 6 mo	27.2 (25.6)	37.4 (30.9)	5.9 (15.3 to −3.5)
Walking self-efficacy overall score, %^b^			
Baseline	52.5 (35.0)	58.7 (33.4)	NA
At 3 mo	46.6 (32.9)	56.8 (30.3)	6.6 (17.5 to −4.2)
At 6 mo	46.2 (38.0)	55.7 (33.2)	5.0 (17.1 to −7.0)

Abbreviations: IPAQ-SF, International Physical Activity Questionnaire short form; MET, metabolic equivalent of task; NA, not applicable.

^a^ The model estimates the mean difference between intervention and control from a common baseline so that any change over time between the groups is not due to any difference in baseline scores. Differences use the control group as the reference group.

^b^ Score range, 0% to 100%, with lower scores indicating lower self-efficacy.

The intervention was superior to control for global HRQOL at 3 months (mean difference, 7.1 points [95% CI, 0.4-13.8 points]), but there were no statistically significant between-group differences in global HRQOL at 6 or 12 months (eTable 2 in Supplement 2). Generally, symptoms were not statistically different between groups at 3, 6, or 12 months. The exception to this was appetite loss favoring the intervention at 3 months, and “pain in other parts” (different from scores for overall pain, pain in chest, pain in arm, and pain in shoulder) favoring the control at 3 months. No between-group statistically significant differences were seen in sleep quality or financial toxicity. The survival probability at 12 months between groups was similar (intervention, 0.97 [95% CI, 0.87-0.99]; control, 0.95 [95% CI, 0.85-0.98]).

## Discussion

In this multisite randomized clinical trial involving patients undergoing surgery for lung cancer, a remote 3-month, physiotherapy-led, home-based exercise and self-management program did not show a statistically significant between-group difference in self-reported physical function compared with control 3 months postoperatively. However, statistically significant and clinically meaningful between-group differences favoring the intervention were detected in objectively measured physical function, exercise capacity, exercise self-efficacy, and global HRQOL.^31,39,40,41^ The 6-minute walk difference observed at 6 months was above the MCID for lung cancer (22-42 m),^40^ and the Short Physical Performance Battery score difference was within the MCID range for patients with chronic obstructive pulmonary disease (0.83-0.96 points).^39^ The global HRQOL difference at 3 months was also within the MCID range for patients with cancer undergoing treatment.^41^ Prior lung cancer trials have found similar improvements with exercise postoperatively,^8,9,10,42^ yet the novelty of our trial is the intervention design—an unsupervised, telephone-supported, and home-based design that is a simpler and less resource-intensive model that is potentially more easily implementable into clinical practice than center-based, supervised exercise programs.^8,9,10,42^ We recommend our model be considered for implementation into lung cancer care given the failure of widespread implementation of center-based, supervised exercise programs.

The objective benefits seen in physical function were not mirrored in subjective perception of benefit (measured by the EORTC QLQ-C30). These conflicting findings may be due to patients overestimating their physical function, a potential ceiling effect of the measure, or regression to the mean phenomenon. Self-reported physical function is influenced by psychosocial and environmental factors, and this result may indicate an underlying response shift and recalibration of patients’ self-assessment based on their diagnosis; this concept has been reported in other populations.^43,44,45^ Self-report and objective measures assess different constructs in relation to physical functioning.^46^ The EORTC QLQ-C30 works well in drug trials but may lack sensitivity to pick up the effect of multifaceted, complex health interventions that are nested in usual care. Future trials should carefully consider their choice of primary outcome measure.

The focus on behavior change strategies within our intervention may have resulted in the longer-term effect on physical function and exercise capacity observed beyond the conclusion of the 3-month program. For example, the intervention may have empowered patients to maintain a physically active lifestyle through self-efficacy and habit formation. This is an important finding, as most prior hospital-based lung cancer exercise trials have not observed a sustained difference in exercise capacity beyond the conclusion of the exercise program.^8,9,10,42^ At 3 and 6 months’ follow-up, our intervention patients were in the moderately confident range (40%-60%) to exercise despite barriers, compared with control patients, who were in the slightly confident range (20%-40%) to exercise despite barriers.^31^

Our intervention appears to have mitigated the decline in functioning that is commonly observed in patients with lung cancer.^2,3,4,5,6^ Maintaining functioning may be just as important as improving it in this cohort. Physical activity level is a highly variable outcome measure, and therefore it is difficult to observe significant changes in small trials. Although the MET minutes per week did not reach statistical significance, the mean and the upper bound of the 95% CI at 3 months favored the intervention group. The lack of change in muscle strength may be explained by the focus of the intervention (behavior change), which contrasts with exercise programs with rigid parameters around dosage.

Although statistical or clinically meaningful differences were not detected for self-reported physical function, the findings from our study suggest that this simple, resource-efficient, home-based program may improve physical function (objectively measured), HRQOL, exercise capacity, and self-efficacy. Future research focused on these outcomes is required to verify findings. Research is required to understand scalability across different contexts. The intervention may be valuable in low- to middle-income countries where the burden of lung cancer diagnosis is highest, as it requires minimal infrastructure due to the simplicity of the program design.

### Strengths and Limitations

This study has some strengths. The robust randomized clinical trial design with assessor blinding, excellent participant retention, and intervention adherence, as well as the use of reliable and valid outcomes all enhance the internal validity. The rigorous intervention development was informed by behavior change theory and refined based on input from clinicians and people with lived experience of lung cancer.^15^ Generalizability was maximized by broad inclusion criteria and recruitment across multiple hospitals.

This study also has some limitations. There is a potential for bias due to lack of inclusion of people from non–English-speaking backgrounds and the inability to blind participants and physiotherapists. The study was not powered to detect between-group differences in secondary outcomes, and our sample was already physically active at baseline. The comparator was usual care, and 12.1% of our cohort (evenly balanced across groups) were referred to pulmonary rehabilitation; it remains unknown how the intervention compares with supervised center-based exercise training, and this comparison is an important line of inquiry for future research.

## Conclusions

In this randomized clinical trial of a postoperative home-based exercise and self-management program, the program did not improve self-reported physical function in patients with lung cancer. However, secondary outcomes revealed improvements in objectively measured physical function, exercise capacity, exercise self-efficacy, and global HRQOL. Implementation of this program into lung cancer care should be considered.

## Notes

Supplement 1.
Trial Protocol and Statistical Analysis Plan

Supplement 2.
eMethods.
**eTable 1.** Physical Function (Primary Outcome) Measured With the EORTC QLQ-c30 at 3 Months Post-operatively, Including Subgroup Analysis **eTable 2.** Summary of Continuous Secondary Outcomes (Health-Related Quality of Life and Symptoms) With Measures at Baseline **eTable 3.** Summary of Secondary Outcome (IPAQ-SF Categorical Outcome for Activity Level) **eFigure.** Mean Objectively Measured Physical Function (Short Physical Performance Battery) Over Time
eReferences.

Supplement 3.
Data Sharing Statement
