# Supplementary material for: Home-Based Exercise and Self-Management After Lung Cancer Resection: A Randomized Clinical Trial
Source: JAMA Netw Open. 2024 Dec 2;7(12):e2447325. doi: 10.1001/jamanetworkopen.2024.47325 (PMC11612835; doi:10.1001/jamanetworkopen.2024.47325)
Supplement: Supplement 1. — Trial Protocol and Statistical Analysis Plan [file jamanetwopen-e2447325-s001.pdf]

Supplement to "Home based exercise and self-management following lung cancer resection: a randomized clinical trial"

Table of contents

1.1 Trial Protocol……2

1.2 Amendments to original protocol, including pauses to recruitment during screening period……31

1.3 Statistical Analysis Plan……34

Supplement 1.

1.1 Trial Protocol

Cancer And Physical ACtivITY (CAPACITY) trial: A randomised control trial of exercise and self-management for people with lung cancer

Protocol Number: 1
Version: 6
Date: 14/09/2022

Author/s:
A/Prof Catherine Granger, A/Prof Louis Irving, Mr Phillip Antippa, Prof Mei Krishnasamy, Dr Selina Parry, Dr Karen Lamb, Ms Shaza Abo, A/Prof Gavin Wright, Prof Christine McDonald, Ms Jane Mack, Ms Georgina Whish-Wilson and Prof Linda Denehy

Funding:
Victorian Cancer Agency Clinical Research Fellowship (CRF16011)- Dr Granger (awarded 2016);
The University of Melbourne, Melbourne Clinical and Translational Sciences (MCATS) research platform seed grant (awarded 2016).
Cancer Council Victoria Grant-in-Aid

CONFIDENTIAL
This document is confidential and the property of The University of Melbourne and Royal Melbourne Hospital. No part of it may be transmitted, reproduced, published, or used without prior written authorization from the institution.

Statement of Compliance
This study will be conducted in compliance with all stipulation of this protocol, the conditions of the ethics committee approval, the NHMRC National Statement on ethical Conduct in Human Research (2007) and the Note for Guidance on Good Clinical Practice (CPMP/ICH-135/95).

TABLE OF CONTENTS

TABLE OF CONTENTS 4
1. GLOSSARY OF ABBREVIATIONS & TERMS 10
2. STUDY SITE 10
2.1 STUDY LOCATION 10
3. INTRODUCTION/BACKGROUND INFORMATION 12
3.1 LAY SUMMARY 12
3.2 BACKGROUND INFORMATION 12
4. STUDY OBJECTIVES 14
4.1 STUDY AIMS AND HYPOTHESES 14
4.2 OUTCOME MEASURES 14
5. STUDY DESIGN 18
5.1 STUDY TYPE & DESIGN & SCHEDULE 18
5.2 USUAL CARE AND INTERVENTION 20
5.3 RANDOMISATION 22
5.4 STUDY METHODOLOGY 22
6. STUDY POPULATION 23
6.1 RECRUITMENT PROCEDURE 23
6.2 INCLUSION CRITERIA 23
6.3 EXCLUSION CRITERIA 23
6.4 CONSENT 24
7. PARTICIPANT SAFETY AND WITHDRAWAL 24
7.1 RISK MANAGEMENT AND SAFETY 24
7.2 HANDLING OF WITHDRAWALS 24
8. STATISTICAL METHODS 24
8.1 SAMPLE SIZE ESTIMATION & JUSTIFICATION 25
8.2 STATISTICAL METHODS TO BE UNDERTAKEN 25
9. DATA SECURITY & HANDLING 26
9.1 DETAILS OF WHERE RECORDS WILL BE KEPT & HOW LONG WILL THEY BE STORED 26
9.2 CONFIDENTIALITY AND SECURITY 26
9.3 ANCILLARY DATA 26
10. APPENDIX 26
11. REFERENCES 27
LIST OF ABBREVIATIONS 39
1. INTRODUCTION 40
1.1 Preface 40
1.2 Purpose of the SAP 40
2 STUDY OBJECTIVES AND ENDPOINTS 41
2.1 Study Objectives and Hypotheses 41
2.2 Outcome measures 41
2.3 Safety outcomes 42
2.4 Other outcomes 42
3 STUDY METHODS 43
3.1 Study Design and Plan 43
3.2 Intervention groups 44
3.3 Inclusion-Exclusion Criteria 44
3.4 Randomisation and Blinding 45
3.5 Sample Size 45
3.6 Study Visit Schedule 46
3.7 Outcome Variables 47
3.7.1 Physical function 48

3.7.2 Health-related quality of life and symptoms 49
3.7.3 Functional exercise capacity 51
3.7.4 Muscle strength 51
3.7.5 Physical activity levels 52
3.7.6 Self-efficacy for physical activity 53
3.7.7 Fatigue 53
3.7.8 Distress 53
3.7.9 Sleep 54
3.7.10 Financial toxicity 54
3.7.11 Return to work/Return to usual activities 55
3.7.12 Survival 55
3.7.13 Hospital re-admissions 55
3.7.14 Outcomes not being covered in the main study analysis 55
4 General Considerations 55
4.1 Timing of Final Analysis 55
4.2 Analysis Populations 56
4.2.1 Full Analysis/Randomised Population 56
4.2.2 Per Protocol Population 57
4.2.3 Safety Population 57
4.3 Covariates and Subgroups 57
4.3.1 Covariates 57
4.3.2 Subgroups 57
4.4 Missing Data 57
4.5 Interim Analyses and Data Monitoring 57
4.6 Multi-centre Studies 58
4.7 Multiple Testing 58
4.8 Estimand Framework 58
5 Summary of Study Data 59
5.1 Subject Disposition 59
5.2 Derived variables 59
5.3 Protocol deviations and violations 59
5.4 Baseline Demographic Variables 59
5.5 Treatment Compliance 60
6 Efficacy Analyses 61
6.1 Primary Efficacy Analysis 61
6.2 Secondary Efficacy Analyses 61
6.3 Estimand-to-analysis for primary and secondary objectives 62
6.4 Exploratory Efficacy Analyses 65
6.4.1 Complier average causal effect 65
6.4.2 Subgroup analyses 65
7 Safety Analyses 65
7.1 Adverse Events 65
7.2 Serious Adverse Events 66
8 Figures 66
9 Reporting Conventions 66
10 Technical Details 66
11 Summary of Changes to the Protocol 66
Appendix A – Table and Figure Listing for the Final Report 67
Appendix B – Templates for tables and figures 68
Appendix C – Data Mapping to Database 81

STUDY SYNOPSIS

Title:	Cancer And Physical ACtivITY (CAPACITY) trial: A randomised control trial of exercise and self-management for people with lung cancer
Short Title:	CAPACITY trial
Design:	Assessor blinded, two-arm superiority randomised controlled trial
Study Centres:	Royal Melbourne Hospital<br>St Vincent's Hospital Melbourne<br>Austin Hospital
Hospital:	Royal Melbourne Hospital<br>St Vincent's Hospital Melbourne<br>Austin Hospital
Study Question:	What is the benefit of an exercise and education self-management program for people undergoing surgery for lung cancer?
Primary Objectives:	In comparison to usual care (no exercise intervention) this study aims to:<br>1: Test the effect of a self-management program (exercise and education) on physical function at 3 months post-operatively in patients with operable lung cancer.<br>Hypothesis 1: The self-management program compared with usual care will improve physical function at three months after surgery.
Secondary Objectives	In comparison to usual care (no exercise intervention) this study aims to:<br>2: Test the effect of a self-management program (exercise and education) on physical activity, muscle strength and function, health-related quality of life (HRQoL), sleep and symptoms in patients with operable lung cancer at three, six and 12 months after surgery.<br>Hypothesis 2: The self-management program compared with usual care will improve physical activity, muscle strength and function, health-related quality of life (HRQoL), sleep and symptoms in patients with operable lung cancer at three, six and 12 months after surgery.<br>3: Test the effect of a self-management program (exercise and education) on health care resource usage and

	financial burden for patients with operable lung cancer at 12 months after surgery. Hypothesis 3: The self-management program compared with usual care will reduce health care resource usage (including hospitalisations, length of stay and intensive care admissions) in the first 12 months after surgery and reduce financial burden for patients.
Inclusion Criteria:	- Adults, aged 18 years or over<br>- Able to provide consent<br>- Planned to receive surgical treatment for non-small cell lung cancer (NSCLC)<br>- Expected to be alive > 6 months<br>- Surgeon or physician approval<br>- Eastern Cooperate Oncology Group (ECOG) performance status of 0-2 at study entry<br>- Not currently meeting the physical activity guidelines (150 minutes of moderate intensity physical activity per week)
Exclusion Criteria:	Participants will be excluded if they meet any of the following:<br>- Non-English speaking (insufficient English language skills to complete the questionnaires)<br>- Metastatic disease (stage IV lung cancer)<br>- Acute uncontrolled cardiovascular or respiratory issues<br>- Decompensated heart failure, severe aortic stenosis, uncontrolled arrhythmia, or acute coronary syndrome<br>- Non-ambulant (for example: amputee, spinal cord injury, wheel-chair bound)<br>- ECOG performance status of 3 or 4 at study entry<br>- Cognitive impairment (determined as not being able to provide consent for surgery)
Number of Planned Subjects:	112
Intervention:	Participants in both arms will receive usual medical, nursing and allied health care. Usual physiotherapy care does not routinely involve assessment or treatment pre-operatively or after discharge from the acute hospital stay.<br><br>In addition to usual care, participants in the intervention arm will receive an exercise and education self-management program. The program consists of two appointments with physiotherapists (prior to hospital discharge post surgery). This includes 1) assessing patient's readiness for physical activity (PA) behavioural change, goals, confidence; 2) verbal education for patient/carer/family about PA; 3) provision of resources to support PA (activity monitor – Garmin watch, exercise diary, pamphlets); 4) setting personalized PA goals and a home program; and 5) identifying barriers/enablers to

	achieve PA goals. Additionally, the patient will receive weekly phone calls to promote adherence to PA goals, discuss barriers/issues and progress exercise. The intervention is designed to be equitable and inclusive of rural patients- appointments are timed with surgical consults and additional intervention is phone-based. The intervention continues until 3 months post-surgery.
Safety considerations:	The tests and exercise program to be applied in this trial are unlikely to cause physical and/or psychological distress. The assessments will be performed by a trained physiotherapist. Participants may find the walking and functional tests and the exercise program tiring as these are types of physical activity. Participants will be provided a rest between these tests. If a participant reports an injury associated with exercise at home they will be referred to their general practitioner for review.
Statistical Methods:	The randomisation list will be devised by the independent statistician and carried out through a central telephone service to ensure allocation concealment. Following consent and assessment, participants are randomised 1:1 (intervention or usual care).<br><br>All data analyses will be undertaken on an intention-to-treat approach. We expect missing data will be missing completely at random (MCAR) so missing data will be ignored unless otherwise specified. For the primary analysis, the distribution of mean change in EORTC QLQ-C30 physical function domain from baseline to 3 months will be compared between the two arms (intervention and usual care) using a Wilcoxon rank sum test.
Subgroups:	Sub-group analyses will be conducted between participants who receive post-operative chemotherapy and or radiotherapy and participants who do not receive any post-operative chemotherapy or radiotherapy, as these groups have a different recovery path following surgery and may respond differently to the exercise intervention.

1. Glossary of Abbreviations & Terms

Abbreviation	Description
COST	COmprehensive Score for financial Toxicity
ECOG	Eastern Cooperate Oncology Group
EORTC QLQ C30 and LC13	European Organisation for the Research and Treatment of Cancer Quality of Life Questionnaire and Lung Cancer Module
HADS	Hospital Anxiety and Depression Scale
HRQoL	Health-Related Quality of Life
IPAQ	International Physical Activity Questionnaire
NSCLC	Non-small Cell Lung Cancer
PA	Physical Activity
PASE	Physical Activity Scale for the Elderly
RMH	Royal Melbourne Hospital
SPPB	Short Physical Performance Battery
QALYs	Quality adjusted life years
6MWD	Six Minute Walk Distance

2. Study Site

2.1 Study Location

Site	Address	Contact Person	Phone	Email
Royal Melbourne Hospital	Level 3 Physio Department, 300 Grattan Street Parkville Victoria	Dr Catherine Granger		
St Vincent's Hospital Melbourne	Department of Cardiothoracics<br><br>41 Victoria Parade Fitzroy 3065	A/Prof Gavin Wright		

St Vincent’s Private Hospital (Fitzroy)	59 Victoria Parade Fitzroy 3065	A/Prof Gavin Wright		
Austin Hospital	Austin Hospital, Department of Respiratory and Sleep Medicine, Harold Stokes Building, Level 5, Heidelberg VIC 3084<br><br>PO Box 5555, Heidelberg, VIC, 3084PO Box 5555, Heidelberg, VIC, 3084	Prof Christine McDonald AM		

3. Introduction/Background Information

3.1 Lay Summary

The majority of people with operable lung cancer now survive yet they suffer significant physical hardship. New models of care are required to minimise morbidity for this large and vulnerable group. This project will test the benefit of an exercise and education self-management program, compared to usual care, for people undergoing surgery for lung cancer to improve their physical function and functional recovery. The project will involve 112 people undergoing surgery for lung cancer in Victoria, Australia. Participants will be randomised before surgery to either the intervention arm (exercise and education self-management program) or the usual care (no exercise program) arm. Participants in both arms will receive usual medical, nursing and allied health care. In addition to usual care, participants in the intervention arm will receive an exercise and education self-management program. The program consists of two appointments with a physiotherapist (before hospital discharge post-operatively). Additionally, the patient will receive weekly phone calls up to 12 weeks after surgery to promote adherence to physical activity goals, discuss barriers/issues with their exercise and progress their exercise program. The intervention is designed to be equitable and inclusive of rural patients, as measurement and intervention appointments are timed with surgical consults and additional intervention is phone-based. Participants in both arms will undergo a battery of tests with a blinded assessor including measurement of physical function, quality of life and physical activity at before surgery, at hospital discharge, and at 12 weeks, 6 months and 12 months post-surgery. We will also follow up to look at health care resource usage (including hospital length of stay and hospital readmission) and the cost effectiveness of the intervention over 12 months from surgery. This study will provide important information on the benefit of an exercise based intervention for people with lung cancer.

3.2 Background information

Lung cancer significantly impacts on the health and wellbeing of Australians, the community and the healthcare system [1, 2]. The majority of people with operable lung cancer now survive (stage I-II five year survival is 38 to 76%) [3], however the burden on the patient (activity limitations, participation restrictions and diminished health-related quality of life [HRQoL]) and healthcare system (high hospital utilization) is high [1, 2, 4]. There is a need for improved models of care [5]. Our vision through the generation of high quality research is to improve the quality of survival of people with lung cancer through feasibly and sustainably implementing exercise into the model of care.

Physical inactivity is a global pandemic [6]. There are strong well-established evidence-based guidelines regarding the amount of physical activity (PA) that people with cancer should undertake [7-9]. The PA guidelines state that individuals with cancer should engage in 30 minutes of moderate intensity PA on five or more days of the week [8]. These guidelines are supported by a strong evidence base demonstrating that increased levels of PA in cancer are associated improved physical function, fitness, HRQoL and lower levels of symptoms and depression [10]. In breast and colon cancer, increased PA is also associated improved survival [11, 12]. Exercise is an inexpensive, safe, simple and powerful treatment in cancer. However the evidence has not translated into clinical practice and patients are not therefore currently receiving best-practice [5]. Our research program targets this evidence-practice translation gap.

In part I of our research program we led the first study to measure PA levels of people with lung cancer in Australia (Dr Granger's PhD project; study funded by 2010 Victorian Cancer Agency Palliative/Supportive Care Capacity Building Grant) [4, 13]. Including 50 patients with stage I-IIIB non-small cell lung cancer (NSCLC) from Royal Melbourne Hospital (RMH), Peter MacCallum and Austin Hospital, and 35 aged-matched healthy controls, we found people with lung cancer have low PA levels (33% fewer steps/day than healthy-controls at diagnosis) and suffer detrimental functional decline (measured by the six minute walk distance 6MWD) over first 6-months post-surgery (Figure 1) [4, 13]. Only 40% of patients met PA guidelines at diagnosis, 26% during treatment and 31% at 6 months. Patients who were more active had better HRQoL, physical function and fitness, and less depression and symptoms [4, 13].

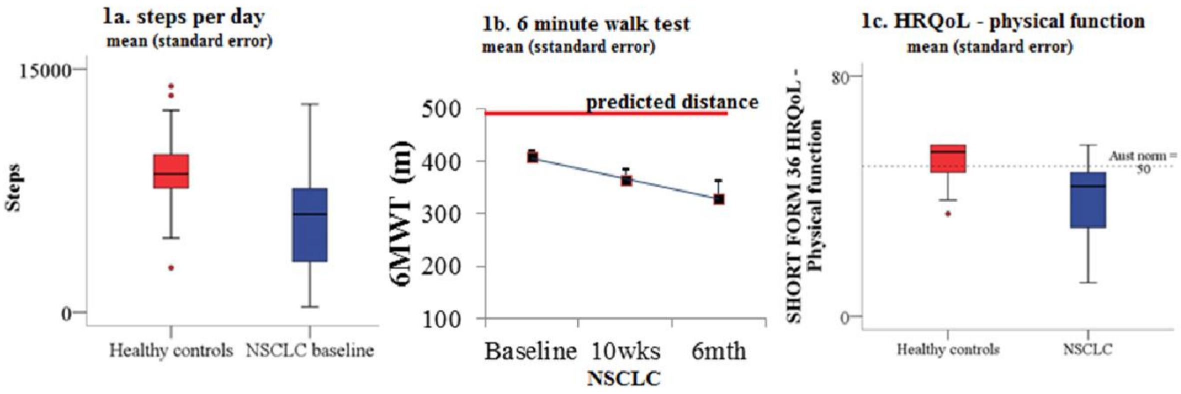

Figure 1: Low PA levels (1a) and poor HRQoL (1c) compared to aged matched healthy controls; and decline in functional capacity over 6 months from diagnosis (1b)[4, 13]

Importantly, only 8% of patients had access to exercise treatment in the first 6 months after diagnosis [4, 13]: this is a significant issue and will not change without changes to the model of care. Current outpatient exercise programs/services (i.e. pulmonary rehabilitation) are at full capacity with waiting lists and expansion of their inclusion criteria beyond respiratory diseases [14] to cancer is not possible. An alternative, cost-effective, low-resource and feasible model is required to effectively and sustainably implement exercise into cancer care to improve patient outcomes. We developed a new model and investigated the feasibility of its implementation (PART II) in 2014 as described below.

In part II of our research program (Dr Granger funded by 2013 NHMRC/Cancer Australia Translating Research Into Practice Fellowship) we piloted a new model, which included an exercise and education self-management program for patients before and after surgery for lung cancer [15]. The pilot (n=42 patients, RMH 2014 - 2015) demonstrated high feasibility (88% consent rate), safety (no adverse events), inclusion of rural patients, high patient satisfaction (mean 9.8/10 global satisfaction score) and was associated with trends of efficacy including improved global HRQoL (European Organisation for Research and Treatment of Cancer EORTC-QLQ-C30 questionnaire mean difference +21.4, 95%CI 7.9–35.0, p=0.005) and maintained PA levels (p=0.416) from pre-operatively to 6-months post-surgery. Patient's confidence to exercise increased from 5.4/10 to 9.2/10. As part of this research we also investigated barriers and facilitators to implementation of exercise using quantitative and qualitative methodology with 17 multi-disciplinary clinicians and 8 consumers, and a systematic review (including 1074 patients, 23 carers and 169 clinicians) [16, 17] - these results informed the current RCT proposal for Part III.

For part III of our research program we are now conducting a world first clinical trial to evaluate this exercise and education self-management program. The current trial is funded by the Victorian Cancer Agency via a Clinical Research Fellowship for Dr Granger (2017 - 2023) and a Cancer Council Victoria grant in aid.

4. STUDY OBJECTIVES

4.1 STUDY AIMS AND HYPOTHESES

In comparison to usual care (no exercise intervention) this study aims to:

1. Test the effect of a self-management program (exercise and education) on physical function at 3 months post-operative in patients with operable lung cancer. Hypothesis 1: The self-management program compared with usual care will improve physical function at three months after surgery.

2. Test the effect of a self-management program (exercise and education) on physical function, physical activity (levels and self-efficacy), muscle strength and function, health-related quality of life (HRQoL), sleep and symptoms in patients with operable lung cancer. Hypothesis 2: The self-management program compared with usual care will improve physical function, physical activity (levels and self-efficacy), muscle strength and function, health-related quality of life (HRQoL), sleep and symptoms in patients with operable lung cancer at three, six and 12 months after surgery.

3. Test the effect of a self-management program (exercise and education) on health care resource usage and financial burden for patients with operable lung cancer at 12 months after surgery. Hypothesis 3: The self-management program compared with usual care will reduce health care resource usage (including hospitalisations, length of stay and intensive care admissions) in the first 12 months after surgery and reduce financial burden for patients.

4. Exploratory aim: to explore the cost effectiveness of a self-management program (exercise and education) for patients with operable lung cancer. Hypothesis 4: The self-management program will be cost-effective compared to current standard treatment in patients with operable lung cancer at 12 months after surgery.

4.2 OUTCOME MEASURES

Outcomes will be evaluated at the following five time points: prior to surgery, prior to hospital discharge post-operatively, and 12 weeks, 6 months and 12 months post-operatively. Please refer to Figure 2 for the time schedule for each test (not all tests are completed at every time point). This testing will take up to 90 minutes per testing session.

The primary outcome is physical function measured by physical function domain of the European Organization for the Research and Treatment of Cancer questionnaire (EORTC QLQ C3) [18] (primary endpoint). We are also measuring physical function using two objective tests: the 6 minute walk distance [19-21] and the Short Physical Performance Battery (SPPB) [22] although these are secondary end points. The study is powered for

the primary outcome measure of physical function domain of the EORTC QLQ C30 not the secondary outcomes.

The EORTC is a self-reported questionnaire that assesses HRQoL over the past week. The 30 item core questionnaire includes nine multi-item scales compromised of five functional scales (physical, role, emotional, cognitive and social functioning), three symptom scales (pain, fatigue and nausea/vomiting) and a global health status/quality of life scale and six single-item scales (appetite loss, dyspnoea, diarrhea, constipation, insomnia and financial impact [18]. The 13 item LC13 supplementary module includes multi and single items scales measuring symptoms and treatment side-effects specific to lung cancer [23]. Responses to most questions are rated on a four point Likert type scale according to occurrence in the past week (not at all, a little, quite a bit and very much). All domain and single-item raw scores are linearly transformed to a scale of zero to 100 [24]. Higher scores on functional domains and global health status/quality of life scale represent higher functioning and higher HRQoL. Lower scores on symptom domains and single-items represent less symptoms [18, 24]. The EORTC-QLQ-C30 core questionnaire has strong test-retest reliability for the physical function domain (r=0.91) [25] and is responsive to change from pre-treatment to during-cancer treatment [18, 23].

The 6 Minute Walk Distance (6MWD) is a commonly used and validated surrogate measure of submaximal exercise tolerance, with high clinical utility [26]. Participants are asked to walk up and down a 25 – 30m flat straight corridor and cover as much ground as possible in six minutes [21]. Studies have demonstrated a familiarisation effect of the 6MWD, with the second 6MWD increasing 9 to 15m during a testing session [27]; therefore guidelines recommend the use of two repeated 6MWD [26]. The 6MWD has moderate to strong criterion-concurrent validity against the gold standard measure of functional capacity (cardio-pulmonary exercise testing) (r = 0.51 – 0.93) [28-31]. Inter-rater reliability for the 6MWD in chronic lung and cardiac disease is strong (ICC = 0.90 - 0.93) [32-35]. Responsiveness has been demonstrated by multiple studies, with the distance increased in individuals undergoing thoracic surgery or participating in an exercise intervention [31]. We have previously shown that the 6MWD deteriorates by 78m over six months in a usual care cohort (effect size = 0.7) of 90 patients with lung cancer stages I-IV and that ceiling effects occurred in only 3.6% of patients [19]. We have also shown that the minimal important difference in lung cancer is 22 to 42m [19].

The Short Physical Performance Battery (SPPB) is an objective measure of physical function [22] consists of three tests: 1) Gait speed: participants are instructed to walk a distance of eight feet (2.4 meters) and the average of two trials are used; 2) Standing balance: participants are assessed in three different static positions (side-by-side stand, semi-tandem stand and tandem stand) for 10 seconds each; and 3) Chair rise task: participants are instructed to stand up and sit down five times in a row as quickly as possible. Each individual test is scored on a scale of zero to four points (higher scores are better performance). The three test scores are summated to give an overall SPPB performance score ranging from zero to 12 points. A zero score indicates poor function whilst 12 indicates excellent function. If the participant is unable to physically perform a specific test, a score of zero is assigned. It has been previously reported in the literature that for older adults a score of 10 is considered the cut-off for mobility impairment (i.e. scores <10 = poor mobility) [36].

Secondary outcomes:

The following secondary outcomes will also be assessed.

Objective tests:

- *Quadriceps muscle Strength:* Participants will have their isometric peripheral muscle strength measured in bed for quadriceps muscles using the MicroFET hand held dynamometer (which can be used to assess peripheral musculature). This device has been shown to have robust measurement properties (both reliability and validity) against the criterion reference KinCom laboratory based dynamometer [37]. These muscles will be assessed using standardised methodology in a seated position. The highest value achieved among three maximum contractions with a coefficient of variation less than 10% will be recorded.

- *Quadriceps muscle ultrasound Imaging:* Ultrasound imaging will be conducted using Sonosite iViz ultrasound machine with a linear transducer by the blinded assessor physiotherapist. Device settings will be kept constant between participants and across timepoints. The quadriceps will be measured at 2/3 distance from anterior superior iliac spine to superior border of the patella. Muscle ultrasound is inexpensive, and has high reproducibility and reliability [38].

- *Physical activity levels:* A movement sensor device will be placed on the participant's wrist or hip (depending on patient preference). The device is small (size of a matchbox) and will be worn for up to one week at each time-point of testing and participants will be given a pre-paid addressed envelope to post the device back to the investigators at the end of the week. This has worked in our previous studies.

Questionnaires (please see the appendix for a copy of the questionnaires):

- *Physical activity levels*: Physical activity will be measured using the International Physical Activity Questionnaire (IPAQ) [39] and the Physical Activity Scale for the Elderly (PASE) (Washburn 1999; Washburn 2000; Schuit 1997). There is significant controversy within the literature as to the most suitable questionnaire to assess physical activity and therefore to allow us to compare our results with the recommended guidelines, normative Australian and international data and previous studies we need to use both questionnaires. Participants will be informed that whilst there is some overlap in the questions, it is important to try to complete all questions.

- *Self-efficacy questionnaires:* measured with the barriers, task and walking self-efficacy scales [40, 41] which have been used in cancer exercise trials before and were specifically designed to evaluate self-efficacy of people with cancer.

- *Sleep*: measured with the 8-item Sleep Disturbance – Short Form 8b, a valid measure of sleep disturbance [42].

- *Fatigue*: measured with the Brief Fatigue Inventory [43]. This tool assesses the severity of fatigue and the impact of fatigue on daily function in patients with cancer, and asks about fatigue over the past 24 hours [43].

- *Mood:* measured with the Hospital Anxiety and Depression Scale (HADS) [44, 45]. The HADS is a tool for identification of depression, anxiety and distress [44, 46, 47].

Distress will be recorded using the Distress Thermometer, a modified 11 point visual analogous scale, which asks the participant to rate the distress they have been experiencing over the previous seven days from zero (no distress) to 10 (extreme distress) [48].

- *Financial toxicity:* measured with the COmprehensive Score for financial Toxicity (COST) questionnaire specifically developed and validated to measure financial risk for patients with cancer [49, 50].

- *Return to work:* measured with the Employment questionnaire [51]. This is a newly developed questionnaire by our colleagues studying intensive care rehabilitation in the USA [51]. We have adapted this to refer to lung cancer instead of intensive care as a means to collect these data.

- *Economic analyses:* using the European Quality of Life EQ-5D-5L [52]. This 5-item questionnaire is a commonly used measure of HRQoL that can be used to calculate QALYs (quality adjusted life years) for health economic analysis. For example, we can calculate an incremental cost-effectiveness ratio which will inform dollars per QALY gained as a result of the intervention. This is a useful metric that will allow comparisons with other interventions.

Demographic and medical data will be obtained. This includes age, sex, residential post-code, type of cancer, type of cancer treatment, smoking history, body mass index, respiratory function, social situation, pre-morbid mobility and comorbidities. Comorbidities will be scored with the simplified Colinet comorbidity score. The surgical procedures, complications and hospital length of stay will be recorded. All hospitalisations including costs over a 12-month period will be obtained from routinely collected hospital administrative data. All costs which are relevant to the implementation and delivery of the service will be identified, measured and valued accordingly. This includes the cost to coordinate the project (e.g. project manager), number of staff (and staff time) involved in planning and supporting the intervention (this may include training and service delivery). Participants will be followed up for survival for five years after diagnosis.

Participants in the intervention group will be asked to complete a survey before and after the intervention program to gather their views on the program and ongoing barriers and enablers to continued exercise following conclusion of the intervention (please see Appendix).

A random sample of 15 (minimum) to 20 (maximum) participants in the intervention group will also be asked to participate in a semi-structured interview to further explore their views on the actual program at 3 months post-operatively. The interview will be conducted at a time agreed upon with the patient, using video-conferencing (preferred option), in person (other preferred option) or over the phone (least preferred option) by a physiotherapist member of the research team who was not their treating physiotherapist and did not provide the intervention program to them.

For the video-conferencing option: these will be conducted via Zoom videoconferencing software hosted by The University of Melbourne. Video interviews are preferred over phone interviews due to their ability to facilitate increased rapport between the participant and the interviewer, as well as allowing the interviewer to obtain more accurate field notes. Zoom interview meetings will be password protected, with participants unable to

enter the virtual meeting until being ‘admitted’ from the virtual waiting room by the researcher as a second-line security measure. Interview sessions will be manually ‘locked’ by the researcher, meaning no other participants will be able to enter after the session commences (although interviews will not be scheduled immediately before or after another participant). Participants will be informed of the intent to audio and video record the interview at the time of scheduling and asked for their specific consent to record at the commencement of the interview (as per our original protocol). Participants who decline to be video recorded will be guided by the researcher to turn off their camera during the Zoom interview. Zoom interviews will be both audio and video-recorded via the Zoom application. Telephone interviews will be utilised as a back-up in the event of connection breakdown. To account for the possibility of software failure, back-up audio-only recordings will be taken on an external audio recording device and deleted once the Zoom recording is confirmed to have saved correctly The rationale for obtaining video recordings is to facilitate the researcher to be able to expand upon field notes after the interview concludes, ensuring their accuracy and detail. This will allow one sole researcher to complete the interviews and not rely on a second researcher to be present. All video and audio recordings will be immediately saved onto the RMH server on a locked folder only accessible to the research team.

The interview will be conducted in a semi-structured open format. The interview will be recorded and transcribed at a later date. Transcriptions will be checked by a second researcher. Participants will be emailed or posted (depending on their preference) a summary of the analysis and asked to determine if the interpretation is consistent with their perspective. Participants who perform the interview in person or via video-conferencing will be asked if the interview can be videoed. With their specific consent this will be performed. The rationale for this is if the intervention is effective for implementation into practice. Video clips could be used for 1) “advertising” the program to patients in the future who have been told they need surgery to help motivate them and encourage them to think positively about life after the surgery. This would be a new approach to patient information and preparation pre-surgery; 2) provide excellent material for presenting at conferences and encouraging other clinicians to take up/implement the intervention if shown to be effective; 3) would be great for consumer advocacy and information in terms of what to ask for should they be diagnosed with a need surgery for lung cancer; and 4) great for inclusion as novel teaching resource across VCCC clinical partners. The qualitative aspect of this trial is overseen by Prof Mei Krishnasamy who has extensive experience in patient outcomes and qualitative research in cancer.

5. STUDY DESIGN

5.1 STUDY TYPE & DESIGN & SCHEDULE

This is an assessor blinded, two-arm superiority randomised controlled trial. This will be conducted in at the Royal Melbourne Hospital, St Vincent’s Hospital Melbourne, St. Vincent’s Private Hospital (Fitzroy) and Austin Hospital. 112 participants will be included in this study. The trial will be reported according to the CONSORT Statement extension for non-drug interventions. Following ethical approval, the trial will be registered on clinicaltrials.gov and the protocol and statistical plan will be published.

Participants will also be asked to participate in five testing sessions across the 12 month period with the first testing session just prior to surgery and subsequent sessions

scheduled: before hospital discharge, and at 3 months, 6 months and 12 months post surgery. Each testing session is anticipated to take a maximum of 90 minutes in total. Assessments will occur in the outpatient physiotherapy, thoracic surgery or respiratory medicine departments, however for participants unable to travel/drive to attend or those who prefer not to attend the hospital the hospital for follow-up appointments, will be offered a home visit assessment if they live within a feasible distance of the hospital.

Home Visit Procedure and Contingency Plan:

If a patient is unable or prefers not to attend the hospital for follow up assessments they may be offered a home visit assessment. Home visits will be conducted in line with policies and procedures of the relevant hospital. At Royal Melbourne Hospital, this assessment will be conducted in line with the Melbourne Health Home Visit Screen and Assessment Policy (Number: MH15.22, expiry date 27 Nov 2017). As per the policy guidelines no home visit will occur until a Risk Assessment Plan has occurred (Occupational Health and Safety Home Visit Risk Assessment Form OP/IP 18/NWMH Risk Assessment and Plan form) and it has been deemed safe for the home visit to occur. This form will also be filed with medical records. This will occur at each time that the patient requires a home visit. Any incidents or potential hazards that are identified will be reported on Riskman as per the policy. Assessors performing a home visit will follow the usual home visit physiotherapy procedures including carrying a mobile phone and calling in and out (phoning Dr Catherine Granger, or if Dr Granger is performing the home visit she is to call the Physiotherapy Manager Alana Jacob) before and after the home visit. We expect home visits to be infrequent as we will try to align the follow up assessments with existing medical appointments to minimise participant burden. The costs of home visits for the trial will be recorded.

Plan for Data collection and Storage:

The information collected will be kept in a potentially re-identifiable format – in case after submission for manuscript publication the journal requires further patient information from the medical history. Paper copies of data collection sheets and questionnaires will be coded and stored in locked filing cabinet stored in a locked office which will only be accessible by study personnel. At Royal Melbourne Hospital, these forms will be stored in Dr Granger's research office in Level 5 Allied Health. Data will also be stored in electronic format on password-protected computer. Data will be kept for a minimum of five years after publication and after this time paper copies will be shredded and electronic files deleted.

Table 1: Outcome measures to be administered across study time points

Assessment/ Procedure	Screening	Pre-surgery and before randomisation	Post-operatively before hospital discharge	3 months post-operatively	6 months post-operatively	12 months post-operatively
Informed Consent	x					
Demographic and medical information, including surgical procedure		X	X			

**EORTC QLQ C30 and L13 questionnaire**		**X**		**X***	X	X
Physical function test– 6MWD		X		X	X	X
Physical function test – SPPB		X	X	X	X	X
Muscle strength and function tests		X	X	X	X	X
Physical activity – activity devices, and PA levels questionnaires		X		X	X	X
Self efficacy questionnaires		X		X	X	X
Symptoms, sleep, fatigue, financial burden, return to work and mood questionnaires		X		X	X	X
Intervention satisfaction and knowledge questionnaires (intervention group only)		X		X		
EQ 5D 5L		X		X	X	X
Health care resource usage data over 12 months						X
Pre post surveys (views of program)		X		X		
Interviews with 15-20 participants				X		
Survival follow-up						to 5 years

*Primary endpoint

5.2 USUAL CARE AND INTERVENTION

Usual care (both arms): Usual medical, nursing and allied health care will be provided to both groups. Usual physiotherapy care does not routinely involve assessment or treatment pre-operatively or after discharge from the acute hospital stay. Physiotherapy in the post-operative period (when patients are in hospital after surgery) follows a clinical pathway as per usual practice. This will be audited via the medical records for the purpose

of this study to document the physiotherapy participants in both groups receive. The usual care physiotherapist on the ward will be blinded to group allocation.

Intervention arm: In addition to usual care, participants randomised to the intervention arm will also receive an exercise and education self-management program as tested in our feasibility study [15]. The intervention implements the best available evidence regarding PA for lung cancer [7, 8, 53]. The intervention components are focused around education, training and enablement from the behavioural change wheel framework [54] and on behavioural change support to address patients' behavioural, emotional, situational and/or cognitive barriers to initiating/sustaining action (exercise) [55, 56]. The program consists of two appointments with physiotherapist (just before hospital discharge post-op) and weekly telephone call support up to 12 weeks post-operatively. The following describes the intervention (which was piloted in our previous study) [15]:

1. Post--operative physiotherapy appointment(s). If needed this appointment can be split over two sessions. These sessions are usually delivered while the patient is still in hospital after surgery, but if the patient is discharged home quickly, they can be delivered over the telephone if needed: The first half of the appointment is focused on:
    a. Assessing the patient's current level of PA, fitness, HRQoL and mood
    b. Assessing the patient's readiness for behavioural change regarding PA

    The focus of the second half of the appointment will be:
    c. Educating the patient and their carer(s) about PA. Patients will be given access to resources with the information about PA to take home. Participants will also be given a Garmin activity tracker watch to use during the intervention period to self-monitor their physical activity
    d. Working with the patient and their carer to set personalized and individual goals regarding their participation in PA. Patients with lung cancer present with other comorbid disease, including depression and COPD that are unique to this diagnosis. Therefore to improve efficacy a targeted approach to PA is required, that addresses individual goals and PA prescribed from a baseline PA/fitness test for each individual separately.
    e. Identifying barriers and enablers for the patient to be engaged in sufficient PA and brainstorm strategies to overcome these.

    A focus of the program is Behavioural Change Support. There is growing recognition that provision of treatment education or advice alone is not sufficient to produce significant changes in health behaviour. There are three main groups of processes that are required to support patient behaviour change for adherence to treatment [55]. These are building a strong therapeutic alliance, building or reinforcing motivation and building or reinforcing self-efficacy. The clinical practice model developed from these processes instructs clinicians on how to engage patients in clinical face-to-face and telephone consultations [56]. The model actively addresses individual behavioural, emotional, situational and cognitive barriers to initiating and sustaining action to meet clinical guidelines for PA. A cancer diagnosis may represent "a teachable moment"-a time that survivors are more receptive to health behaviour counselling in PA [57]. Furthermore improvement in PA, symptoms and HRQoL in people with a variety of chronic diseases including lung cancer are reported using these techniques [58 , 59].

2. Weekly follow-up phone calls from the physiotherapist to the patient (and where possible the carer) to promote adherence to PA goals, progress exercise and discuss any new barriers or issues (the phone calls occur each week up until 12 weeks post-op).

Participants will be asked to keep an exercise diary during the intervention period to record their daily exercises.

The costs of delivering the intervention will be recorded.

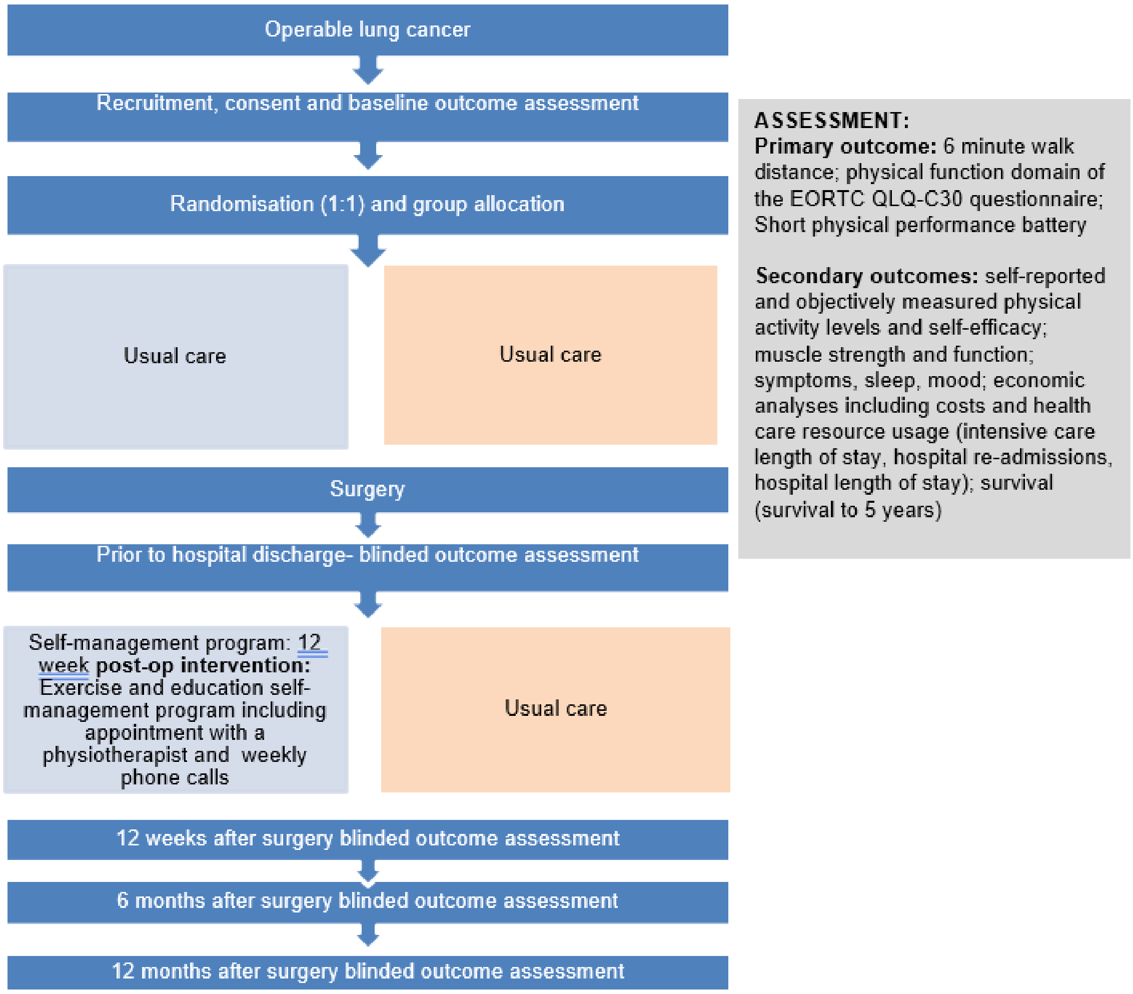

Figure 2: Trial time-line

5.3 Randomisation

The randomisation list will be devised by the independent statistician and carried out through a central telephone service to ensure allocation concealment. Following consent and assessment, participants are randomised 1:1 (intervention or usual care).

5.4 Study Methodology

Procedure: Potentially eligible participants identified through weekly cancer multi-disciplinary team meetings/clinics at the hospitals. At recruitment participants provide written informed consent (if being recruited in person at the hospital) or Redcap e-consent (if consented by the doctor during their telehealth appointment), complete assessment and are randomly allocated off-site to ensure concealment (Figure 2). Reasons for attrition over the 12 month trial period will be collected and assessed.

Intervention methodology: Trained physiotherapists will provide the intervention. An intervention protocol will be developed to ensure standardization of the intervention. The intervention procedures will be strictly monitored for protocol adherence.

Blinding: Blinding of participants and therapists cannot be achieved due to the nature of the intervention. However, assessors blinded to allocation will undertake assessments. Assessor blinding will be assessed with a questionnaire. The usual care ward physiotherapist (who is not involved in the trial intervention) will be blinded to group allocation. Blinding of this physiotherapist will be assessed by a questionnaire.

Outcome measures: will be evaluated across the following time points: prior to surgery, before hospital discharge, 3 months post-operatively, 6 months post-operatively, and 12 months post operatively. This testing will take up to 90 minutes per testing session. The outcomes and the outcome measures are described in section 4.2.

6. STUDY POPULATION

6.1 RECRUITMENT PROCEDURE

Consecutive patients will be screened from lists of patients reviewed at the lung cancer multi-disciplinary team weekly meeting and outpatient clinics (medical oncology, radiotherapy, respiratory lung mass and thoracic surgery) at each hospital. Participants may be recruited any time between time of presentation to the lung clinic/MDT meeting and surgery (this period of time is usually only a few weeks). Participant eligibility for inclusion will be confirmed by their surgeon or respiratory physician. Due to COVID-19 many patients are now seen by their doctor(s) at the lung cancer outpatient clinics via telehealth. At recruitment participants will be asked to provide informed consent (via a hard copy of the PICF if attending the clinic in person, or via Red-cap e-consent if attending the clinic via telehealth), complete assessment and then be randomly allocated to the intervention or usual care arm off-site to ensure concealment (Figure 2).

6.2 INCLUSION CRITERIA

Eligible participants will include:

- Adults, aged 18 years or over
- Able to provide consent
- Planned to receive surgical treatment for non-small cell lung cancer
- Expected to be alive > 6 months
- Surgeon or physician approval
- Not currently meeting the physical activity guidelines (150 minutes of moderate intensity physical activity per week)
- Eastern Cooperate Oncology Group (ECOG) performance status of 0-2 at study entry

ECOG 0	Fully active
ECOG 1	Walking, but only can do light work
ECOG 2	Rest in bed LESS than half the day, do not work but can care for self
~~ECOG 3~~	~~Rest in bed MORE than half the day, and only partially cares for self~~
~~ECOG 4~~	~~Bedridden~~

6.3 EXCLUSION CRITERIA

Participants will be excluded if they satisfy any of the following criteria:

- Non-English speaking (insufficient English language skills to complete the questionnaires)

- Metastatic disease (stage IV lung cancer)
- Acute uncontrolled cardiovascular or respiratory issues
- Decompensated heart failure, severe aortic stenosis, uncontrolled arrhythmia, or acute coronary syndrome
- Non-ambulant (for example: amputee, spinal cord injury, wheel-chair bound)
- ECOG performance status of 3 or 4 at study entry
- Cognitive impairment

6.4 Consent

For this trial, individual consent will be obtained from participants themselves. Potential participants will be asked to provide consent prior to participation (and prior to baseline outcome assessment and randomisation) (Figure 2). According to the National Statement on the Ethical Conduct in Human Research, participation will be voluntary and all information will be given to the participant in order to clarify the purposes, methods, risks and potential benefits of the research.

Due to COVID-19 and the transition to many hospital outpatient appointments being conducted via telehealth, as of 28 October 2021, we added an option for consent via Redcap e-consent for those patients who are attending their lung clinic medical/surgical appointments via telehealth to still allow them the option to participate in the trial.

6.5 LIMITATIONS

With the design of this trial it is not possible to blind the participants or the interventionists. We will ensure the assessors are blinded. Another potential limitation is selection bias in that participants who consent to the trial may be those who are interested in exercise. Another potential limitation is that participants in both groups may be encouraged to exercise based on their participation in this trial. We are recording their exercise levels and will carefully assess the level of exercise (including change) of participants in both groups throughout the study and report on this.

7. Participant Safety and Withdrawal

7.1 Risk Management and Safety

The tests and exercise program to be applied in this trial are unlikely to cause physical and/or psychological distress. The assessments will be performed by a trained physiotherapist who will be with the participant at all times. Participants may find the walk tests and functional tests tiring and the exercise programs as these are types of physical activity. Participants will be provided a rest between these tests. If a participant reports an injury associated with exercise they will be referred to their general practitioner for review.

7.2 Handling of Withdrawals

As with any physiotherapy assessment or treatment patients can choose not to have it. If the patient or their families request not to have physiotherapy assessment this decision will be respected. Based on current clinical practice we do not expect patients to request to be withdrawn from the study due to the assessment and intervention proposed. All assessment tools are safe and quick and testing at each time point will take a maximum of 90 minutes. No adverse events are anticipated to occur during the protocol. If an adverse event does occur the surgeon will be notified immediately.

8. Statistical Methods

8.1 Sample Size Estimation & Justification

Sample size estimation: The sample size is based on a primary hypothesis that participants in the intervention arm will improve physical function measured by EORTC QLQ-C30 physical function domain compared to participants in usual care arm. A total sample size of 88 (44 per arm) provides 80% power with a two-sided alpha = 0.05 to detect a minimum difference of 12.9 in mean outcome between the two arms at follow-up, assuming a standard deviation of 21.3 (from our prior published data [64]). We will recruit 112 participants, to allow for approximately 88 participants after 20% drop out (our current drop-out rate is 18%).

Feasibility: In July 2022 we finished recruitment of the 112 participants. Allowing for all patients to complete the final 12-month assessment, we predict the trial will complete participant data collection by June 2023. The final six months (second half of 2023) will be utilized for completion of data entry, data processing (ultrasound images), statistical analyses, trial reporting, and preparation for dissemination of results (conferences and publications).

8.2 Statistical Methods To Be Undertaken

The statistical analysis plan for this trial is overseen by Dr Karen Lamb; and the health economics and cost effectiveness aspect is overseen by Michelle Tew.

All data analyses will be undertaken on an intention-to-treat approach, according to the intention-to-treat definition set by the FDA the planned treatment regimen) rather than the actual treatment given. It has the consequence that subjects allocated to a treatment group should be followed up, assessed, and analyzed as members of that group irrespective of their compliance with the planned course of treatment. We expect missing data will be missing completely at random (MCAR) so missing data will be ignored unless otherwise specified. For the primary analysis, the distribution of mean change in EORTC QLQ-C30 physical function domain from baseline to 3 months will be compared between the two arms (intervention and usual care) using a Wilcoxon rank sum test. Participants will be excluded in this analysis if a participant's EORTC QLQ-C30 physical function data is not collected at either baseline or 3 months visit. Similar analyses will be performed for change from baseline to 6 and 12 months follow-up. For the secondary objectives, the effect of a self-management program intervention, measured by physical activity, muscle strength and function, exercise capacity, health-related quality of life, sleep and symptoms in patients with operable lung cancer will be analyzed using Wilcoxon rank sum test (continuous outcomes) and Fisher's exact test (binary outcomes). A detailed statistical analysis plan will be documented prior to data base lock

The health economic analysis will a healthcare system perspective. It will consider the implementation costs of the exercise and self-management program, compared with usual care in addition to the downstream cost differences associated with length of stay, complications and formal and informal care post-surgery. An incremental cost-effectiveness ratio will be constructed based on a cost per additional case of hospitalization averted. In addition utility data derived from the EQ-5D will be used to calculate a cost per quality adjusted life year (QALY) gained for the intervention group in comparison to standard care. Extensive one-way and probabilistic sensitivity analyses will be conducted to test the sensitivity of the results.

Qualitative data from interviews will be analysed using thematic analysis [61]. Interview data will be transcribed by one researcher and cross checked by another researcher. De-identified interview transcripts will be uploaded to NVivo 1.0 hosted by The University of

Melbourne for coding. NVivo files will be password protected and accessible only to the research team. Text will be read line by line and themes will be identified to best describe the views and responses of the group. Data will be coded in themes. The following questions will be considered: What are people saying? What are people feeling? What is really important? [62]. Member checking of interpreted data will be used as recommended by Creswell 2009 [63]. This form of member checking involves providing participants with a summary of the analysis of their interview transcript to determine if the interpretation is consistent with the participants' perspective.

9. DATA SECURITY & HANDLING

9.1 DETAILS OF WHERE RECORDS WILL BE KEPT & HOW LONG WILL THEY BE STORED

Study data will be kept in a locked filing cabinet only accessible to researchers. All records will be kept for a minimum of 5 years post study closure.

9.2 CONFIDENTIALITY AND SECURITY

Study data (coded) will be stored in a secure and confidential manner. All participant data will be coded. Only the members of the research team will have access to the study database. Paper data will be kept in a locked filing cabinets in locked areas. Electronic versions will be kept on a password-protected database accessible only to the research team. This data will only be used for purposes of research. Only summary data will be published.

9.3 ANCILLARY DATA

Any muscle ultrasound images collected within this study will be de-identified with images assigned a code. It will not be possible to re-identify the participant the code will not be linked to a re-identifiable database. All images will be stored in a password protected database at RMH to ensure confidentiality. Data collected will be kept for a minimum of 5 years post study closure at RMH.

Addition to plan (14 September 2022): The University of Kentucky collaborators (Dr Mayer and Dr Yuan Wen) have new software (MyoVision MKUS) which enables automated image analysis of the muscle ultrasound images to occur. This software was not available at the time of design of this study when the initial ethics application was submitted. The University of Kentucky will be provided with de-identified ultrasound images to 1) assess the accuracy and reliability of MyoVision MKUS in different patient populations including lung cancer using some of our samples. One of the researchers on our team (Dr Selina Parry) will manually analysed several images (as per the original plan). This analysis will compared against the automated analysis using the MyoVision MKUS software. If appropriate accuracy and reliability exists for our samples, all images from the cohort will then be automatically analysed using this software. No personal health information will be shared. Equal access and ownership of the data will occur between two collaborators.

We will transfer de-identified ultrasound images via One-drive which is only accessible to members of the research team.

10. APPENDIX

List of Attachments included:

Document Name
Demographic data collection form
6MWD testing procedure and recording sheet
Muscle strength testing procedure and recording sheet
Participant questionnaires
Pre and post exercise intervention survey

11. References

1. Australian Institute of Health and Welfare, *Cancer in Australia: an overview, 2014. Cancer series no. 78. Cat. no. CAN 75. Canberra: AIHW.* 2014.
2. Cancer Australia, *Report to the Nation - Lung Cancer 2011. Cancer Australia, Sydney, NSW.* 2011.
3. Cancer Council Australia, *Clinical practice guidelines for the prevention, diagnosis and management of lung cancer.* 2014.
4. Granger, C., et al., *Low physical activity levels and functional decline in individuals with lung cancer.* Lung Cancer, 2014. **83**(2): p. 292-299.
5. Granger, C., *Physiotherapy management of lung cancer.* Journal of Physiotherapy, 2016. **62**(2): p. 60-67.
6. Lee, I.M., et al., *Effect of physical inactivity on major non-communicable diseases worldwide: an analysis of burden of disease and life expectancy.* Lancet, 2012. **380**(9838): p. 219-29.
7. American Cancer Society. *Cancer prevention and early detection facts and figures 2012* 2012 [cited Accessed February 2, 2014; Available from: http://www.cancer.org/acs/groups/content/@epidemiologysurveilance/documents/document/acspc-033423.pdf
8. Schmitz, K., et al., *ACSM roundtable on exercise guidelines for cancer survivors.* Medicine Science Sports Exercise, 2010. **42**(7): p. 1409-1426.
9. WHO, *Global recommendations on physical activity for health.* Geneva: World Health Organization, 2010.
10. Schmitz, K., et al., *Controlled physical activity trials in cancer survivors: a systematic review and meta-analysis.* Cancer Epidemiol Biomarkers Prev, 2005. **14**(7): p. 1588-1595.
11. Meyerhardt, J., et al., *Physical activity and survival after colorectal cancer diagnosis.* Journal of Clinical Oncology, 2006. **24**(22): p. 3527-3534.
12. Ibrahim, E. and A. Al-Homaidh, *Physical activity and survival after breast cancer diagnosis: meta-analysis of published studies.* Medical Oncology, 2011. **28**(3): p. 753-765.
13. Granger, C.L., et al., *Physical activity measured using global positioning system tracking in non-small cell lung cancer: An observational study.* Integrative Cancer Therapies, 2014. **13**(6): p. 482-492.
14. Spruit, M.A., et al., *An official American thoracic society/European respiratory society statement: Key concepts and advances in pulmonary rehabilitation.* American Journal of Respiratory and Critical Care Medicine, 2013. **188**(8): p. e13-e64.
15. Granger, C., et al., *Implementation of the physical activity guidelines in lung cancer: The CAPACITY study.* European Respiratory Journal, 2016. **48**(suppl 60).

16. Granger, C.L., et al., *Understanding factors influencing physical activity and exercise in lung cancer: a systematic review.* Supportive Care in Cancer, 2017. **25**(3): p. 983-999.
17. Granger, C.L., et al., *Barriers to Translation of Physical Activity into the Lung Cancer Model of Care. A Qualitative Study of Clinicians' Perspectives.* Ann Am Thorac Soc, 2016. **13**(12): p. 2215-2222.
18. Aaronson, N., et al., *The European Organization for Research and Treatment of Cancer QLQ-C30: a quality-of-life instrument for use in international clinical trials in oncology.* Journal of the National Cancer Institute, 1993. **85**(5): p. 365-376.
19. Granger, C., et al., *Minimal important difference of the 6-minute walk distance in lung cancer.* Chronic Respiratory Disease, 2015. **12**(2): p. 146.
20. Singh, S.J., et al., *An official systematic review of the European Respiratory Society/American Thoracic Society: measurement properties of field walking tests in chronic respiratory disease.* The European Respiratory Journal, 2014. **44**(6): p. 1447-1478.
21. Holland, A.E., et al., *An official European Respiratory Society/American Thoracic Society technical standard: field walking tests in chronic respiratory disease.* The European Respiratory Journal, 2014. **44**(6): p. 1428-1446.
22. Guralnik, J.M., et al., *A short physical performance battery assessing lower extremity function: association with self-reported disability and prediction of mortality and nursing home admission.* J Gerontol, 1994. **49**(2): p. M85-94.
23. Bergman, B., et al., *The EORTC QLQ-LC13: a modular supplement to the EORTC Core Quality of Life Questionnaire (QLQ-C30) for use in lung cancer clinical trials. EORTC Study Group on Quality of Life.* European Journal of Cancer, 1994. **30A**(5): p. 635-642.
24. Fayers, P., et al., *EORTC QLQ-C30 Scoring Manual (3rd edition). Published by: European Organisation for Research and Treatment of Cancer, Brussels. .* 2001.
25. Hjermstad, M., et al., *Test-Retest Study of the European Organization for Research and Treatment of Cancer Core Quality-of-Life Questionnaire.* Journal of Clinical Oncology, 1995. **13**(5): p. 1254.
26. American Thoracic Society, *ATS statement: guidelines for the six-minute walk test.* American Journal of Respiratory and Critical Care Medicine, 2002. **166**: p. 111-117.
27. Alison, J., et al., *Repeatability of the Six-Minute Walk Test and Relation to Physical Function in Survivors of a Critical Illness.* Physical Therapy, 2012. **92**(12): p. 1556-1563.
28. Solway, S., et al., *A qualitative systematic overview of the measurement properties of functional walk tests used in the cardiorespiratory domain.* Chest 2001. **119**(1): p. 256-270.
29. Jenkins, S.C., *6-Minute walk test in patients with COPD: clinical applications in pulmonary rehabilitation.* Physiotherapy 2007. **93**(3): p. 175-182.
30. Bellet, N., L. Adams, and N. Morris, *Systematic review: The 6-minute walk test in outpatient cardiac rehabilitation: validity, reliability and responsiveness—a systematic review.* Physiotherapy, 2012.
31. Sadaria, K. and R. Bahannon, *The 6-Minute Walk Test: A Brief Review of Literature.* Clinical Exercise Physiology, 2001. **3**(3): p. 127.
32. Guyatt, G.H., et al., *How should we measure function in patients with chronic heart and lung disease?* Journal of Chronic Diseases, 1985. **38**(6): p. 517-524.
33. Andersson, M., et al., *Measuring walking speed in COPD: test-retest reliability of the 30-metre walk test and comparison with the 6-minute walk test.* Primary Care Respiratory Journal, 2011. **20**(4): p. 434-440.
34. Puhan, M., *Interpretation of treatment changes in 6-minute walk distance in patients with COPD.* European Respiratory Journal, 2008. **32**(3): p. 637.
35. Portney, L. and M. Watkins, *Foundations of clinical research applications to practice*. 2nd ed. 2000, Upper Saddle River, New Jersey: Prentice Hall.
36. Guralnik, J.M., et al., *Lower-Extremity Function in Persons over the Age of 70 Years as a Predictor of Subsequent Disability.* New England Journal of Medicine, 1995. **332**(9): p. 556-561.

37. Mentiplay, B.F., et al., *Assessment of Lower Limb Muscle Strength and Power Using Hand-Held and Fixed Dynamometry: A Reliability and Validity Study.* PLOS ONE, 2015. **10**(10): p. e0140822.
38. Puthucheary, Z., *Acute Skeletal Muscle Wasting in Critical Illness.* JAMA, 2013. **310**(15).
39. Craig, C.L., et al., *International physical activity questionnaire: 12-country reliability and validity.* Medicine & Science in Sports & Exercise, 2003. **35**(8): p. 1381-1395.
40. Rogers, et al., *Exercise barrier and task self-efficacy in breast cancer patients during treatment.* Support Care Cancer, 2006. **14**: p. 84-90.
41. McAuley, E. and S. Mihalko, *Advances in sports and exercise psychology measurement.* . 1998: Morgantown, WV: Fitness Information Technology, Inc.
42. Yu, L., et al., *Development of short forms from the PROMIS sleep disturbance and Sleep-Related Impairment item banks.* Behav Sleep Med, 2011. **10**(1): p. 6-24.
43. Mendoza, T.R., et al., *The rapid assessment of fatigue severity in cancer patients: use of the Brief Fatigue Inventory.* Cancer, 1999. **85**(5): p. 1186-96.
44. Bjelland, I., et al., *The validity of the Hospital Anxiety and Depression Scale - An updated literature review.* Journal of Psychosomatic Research, 2002. **52**(2): p. 69-77.
45. Mitchell, A., *Diagnostic validity of the Hospital Anxiety and Depression Scale (HADS) in cancer and palliative settings: A meta-analysis.* Journal of Affective Disorders, 2010.
46. Luckett, T., et al., *A review and recommendations for optimal outcome measures of anxiety, depression and general distress in studies evaluating psychosocial interventions for English-speaking adults with heterogeneous cancer diagnoses.* Supportive Care in Cancer, 2010. **18**(10): p. 1241-1262.
47. Zigmond, A.S. and R.P. Snaith, *The hospital anxiety and depression scale.* Acta Psychiatr Scand, 1983. **67**(6): p. 361-70.
48. Roth, A., et al., *Rapid screening for psychologic distress in men with prostate carcinoma: a pilot study.* Cancer, 1998. **82**(10): p. 1904-1908.
49. de Souza, J.A., et al., *The development of a financial toxicity patient-reported outcome in cancer: The COST measure.* Cancer, 2014. **120**(20): p. 3245-53.
50. de Souza, J.A., et al., *Measuring financial toxicity as a clinically relevant patient-reported outcome: The validation of the COmprehensive Score for financial Toxicity (COST).* Cancer, 2017. **123**(3): p. 476-484.
51. Kamdar, D.B.B., et al., *Joblessness and Lost Earnings After ARDS in a 1-Year National Multicenter Study.* American Journal of Respiratory and Critical Care Medicine. **0**(ja): p. null.
52. Janssen, M.F., et al., *Measurement properties of the EQ-5D-5L compared to the EQ-5D-3L across eight patient groups: a multi-country study.* Quality of Life Research, 2013. **22**(7): p. 1717-1727.
53. Rock, C., et al., *Nutrition and physical activity guidelines for cancer survivors.* CA: A Cancer Journal of Clinicians, 2013. **62**(4): p. 242-274.
54. Michie, S., M. van Stralen, and R. West, *The behvioural change wheel: A new method for characterising and designing behvioural change interventions.* Implementation Science, 2011. **6**(42).
55. Gale, J. and H. Skouteris, *Health coaching: Facilitating health behaviour change for chronic condition prevention and self-management*, in *Handbook of Applied Topics in Health Psychology*, M. Caltabiano and L. Ricciardelli, Editors. 2012, Wiley-Blackwell.
56. Gale, J., *A practical guide to health behaviour change using the HCA approach*. 2012, Health Change Australia: Sydney, Australia.
57. Demark-Wahnefried, W. and L.W. Jones, *Promoting a healthy lifestyle among cancer survivors.* Hematol Oncol Clin North Am, 2008. **22**(2): p. 319-42, viii.
58. Inglis, S.C., et al., *Which components of heart failure programmes are effective? A systematic review and meta-analysis of the outcomes of structured telephone support or telemonitoring as the primary component of chronic heart failure management in 8323 patients: Abridged Cochrane Review.* Eur J Heart Fail, 2011. **13**(9): p. 1028-40.

59. Clark, M., et al., *Motivational readiness for physical activity and quality of life in long-term lung cancer survivors.* Lung Cancer, 2008. **61**(1): p. 117-122.
60. Granger, C.L., et al., *Low physical activity levels and functional decline in individuals with lung cancer.* Lung Cancer, 2014. **83**(2): p. 292-299.
61. Attride-Stirling, J., *Thematic Networks: an analytical tool for qualitative research* Qualitative Research, 2001. **1**(3): p. 385-405.
62. Song, Y., Liu, D., Chen, Y., He, G., *Using focus groups to design a psychoeducation program for patients with schizophrenia and their family members.* Int J Clin Exp Med, 2014. **7**(1): p. 177-185.
63. Creswell, J.W., *Research design: Qualitative, quantitative, and mixed methods approaches*. 2009: Thousand Oaks, CA: Sage.

64. Granger CL, Irving L, Antippa P, Edbrooke L, Parry SM, Krishnasamy M, Denehy L. CAPACITY: A physical activity self-management program for patients undergoing surgery for lung cancer, a phase I feasibility study. Lung Cancer. 2018 Oct;124:102-109. doi: 10.1016/j.lungcan.2018.07.034.

1.2 Amendments to original protocol, including pauses to recruitment due to COVID-19 during screening period

Date	Nature of amendment	Reason for amendment
1st December 2017	Updated methodology related to quadriceps muscle ultrasound measurement: Quadriceps muscle ultrasound measures to be completed day 1 post-surgery before the patient is randomised.	Initially the protocol included quadriceps muscle ultrasound to be completed pre-surgery but this was deemed not feasible and the timing changed to immediately post-operative.<br><br>Note: these data contribute to a secondary analysis and are not reported in the main trial paper. Data will be reported separately.
23rd March 2020	Recruitment paused for trial	Due to COVID-19, we were required to pause participant recruitment.
6th January 2021	Correction to intervention description to more clearly describe that there is one initial face to face appointment (consultation) with the physiotherapist on the ward after surgery before discharge (not two sessions). The session can be split over two days if needed, but the standardised intervention provides one session for patients after surgery before they are discharged home (usually 3-4 days later). Additionally, the word 'pamphlet' was replaced with 'booklet' to more accurately reflect the information to be given to patients given it is 10 pages long.	On reflection it was noted these descriptions were not clear in the protocol and hence the wording was updated. There was an initial error referring to a pre-operative consultation which was not part of the intervention in the current trial.
12th February 2021	Recruitment resumed for trial	Due to easing of COVID-19 restrictions, we were permitted to resume recruitment.
12th February 2021	In-person assessments windows widened from +/- 14 days to +/- 28 days	Due to COVID-19, assessment windows were widened to allow increased possibility of completing in-person assessments outside of any periods of isolation.
13th May 2021	Updated methodology related to qualitative interviews. Qualitative interviews were planned to be conducted on a sub-set of participants allocated to the intervention group (changes made prior to commencement of recruitment into this sub-study). Changes to	Change to timing to allow interviews to occur soon after completion of the intervention period to reduce the limit of recall bias.<br><br>Change to allow videoconferencing for interviews due to COVID-19.

	protocol: interviews will occur at 3 months post-operatively instead of 6 months post-operatively; interviews can be conducted in-person or via video-conferencing (rather than only as an in-person option); and sample size for interviews changed to 'until data saturation is reached' rather than pre-set of 15 participants.	Sample size for interviews updated according to best practice that interviews continue until data saturation rather than a pre-determined set of n=15 as previously noted.<br><br>Note: these data contribute to a secondary analysis and are not reported in the main trial paper. Data will be reported separately.
20th August 2021	Recruitment paused for trial	Due to COVID-19, we were required to pause participant recruitment
17th September 2021	Recruitment resumed for trial	Due to easing of COVID-19 restrictions, we were permitted to resume recruitment.
5th October 2021	In preparing the trial protocol for publication, the planned statistical analyses were refined. The updated version stated:<br>"A constrained longitudinal data analysis model will be used to analyse the primary outcome (EORTC QLQ c30) across all time points (baseline, 3, 6 and 12 months after surgery), with study group, time point and a study group by time point interaction, and recruitment site included in the model. The model will be restricted to have a common baseline mean score across the study groups based on the assumption that there are no differences in the mean outcome between groups at baseline due to randomisation. The absolute difference in mean change in EORTC QLQ c30 physical function domain from baseline between groups will be estimated (including two-sided 95% confidence interval) at 3-months after surgery (primary time point). The constrained longitudinal data analysis model provides valid inference if the missing data mechanism is at most missing at random. Similar analyses will be conducted for the secondary outcomes. The complier average causal effect will be estimated in primary outcome analyses, in addition to the intention-to-treat effect, using collected adherence data. Heterogeneity of the	Previous version stated:<br>"For the primary analysis, the EORTC QLQ-C30 physical function domain at 3 months will be compared between the two arms (intervention and usual care) controlling for EORTC QLQ-C30 at baseline and the hospital site (used to stratify the randomisation) using ANCOVA. Prior to analysis, tests for normality will be undertaken and if the assumptions are violated, non-parametric (bootstrapping) methods will be used. For the secondary objectives, the effect of the intervention, measured by physical activity levels and self-efficacy, physical function, muscle strength and function, exercise capacity, health-related quality of life, sleep and symptoms in patients with operable lung cancer, similar analyses will be performed at 3 month follow-up. To evaluate whether primary and secondary outcomes are maintained over time (3 and 6 months; quality of life to 12 months), a mixed-effects linear model adjusting for baseline will be performed. A detailed statistical analysis plan will be documented prior to data base lock"

	intervention effect according to post-operative cancer treatment (no post-operative treatment/post-operative chemotherapy, radiotherapy or chemoradiotherapy) will be assessed in exploratory analyses by including interactions between post-operative treatment and study group. The number and percentage of participants with adverse events will be summarised by study group. A health economic analysis run alongside the clinical trial.”	
28th October 2021	Addition of option for e-consent via Redcap	Due to COVID-19 many hospital outpatient appointments were converted to telehealth. Protocol amended to add an option for consent via Redcap e-consent for those patients who are attending their lung clinic medical/surgical appointments via telehealth to still allow them the option to participate in the trial.

1.3 Statistical Analysis Plan (SAP)

Protocol HREC 2017.152 V6 14 September 2022
ACTRN12617001283369

Cancer And Physical ACtivITY (CAPACITY) trial: A randomised control trial of exercise and self-management for people with lung cancer

Statistical Analysis Plan

Document Version History

Version Date	Version	Author/s	Signature	Change Description	Reason/Comment
18th August 2023	1	Diana Zannino		Initial release.	Not applicable.
14th December 2023	2	Diana Zannino			Addition of the consideration of using multiple imputation if 3-month data on primary outcome is missing.

CHIEF INVESTIGATOR	A/Prof Catherine Granger<br>Royal Melbourne Hospital
TRIAL STATISTICIANS	Diana Zannino and Karen Lamb<br>University of Melbourne

Trial personnel

SAP Signatures

I give my approval for the attached SAP entitled CAPACITY dated <SAP VERSION DATE>

Chief Investigator

Name / Affiliation	Signature	Date
A/Prof Catherine Granger<br>(Principal investigator)		14/12/23

SAP Authors

Name / Affiliation	Signature	Date
Prof Julie Simpson on behalf of Prof Karen Lamb<br>(Senior statistician)		14/12/2023
Ms Diana Zannino<br>(Trial statistician)		14/12/2023

Table of Contents

List of Abbreviations 39
1 Introduction 40
1.1 Preface 40
1.2 Purpose of the SAP 40
2 Study Objectives and Endpoints 41
2.1 Study Objectives and Hypotheses 41
2.2 Outcome measures 41
2.3 Safety outcomes 42
2.4 Other outcomes 42
3 Study Methods 43
3.1 Study Design and Plan 43
3.2 Intervention groups 44
3.3 Inclusion-Exclusion Criteria 44
3.4 Randomisation and Blinding 45
3.5 Sample Size 45
3.6 Study Visit Schedule 46
3.7 Outcome Variables 47
3.7.1 Physical function 48
3.7.2 Health-related quality of life and symptoms 49
3.7.3 Functional exercise capacity 51
3.7.4 Muscle strength 51
3.7.5 Physical activity levels 52
3.7.6 Self-efficacy for physical activity 53
3.7.7 Fatigue 53
3.7.8 Distress 53
3.7.9 Sleep 54
3.7.10 Financial toxicity 54
3.7.11 Return to work/Return to usual activities 55
3.7.12 Survival 55
3.7.13 Hospital re-admissions 55
3.7.14 Outcomes not being covered in the main study analysis 55
4 General Considerations 55
4.1 Timing of Final Analysis 55
4.2 Analysis Populations 56
4.2.1 Full Analysis/Randomised Population 56
4.2.2 Per Protocol Population 57
4.2.3 Safety Population 57
4.3 Covariates and Subgroups 57
4.3.1 Covariates 57
4.3.2 Subgroups 57
4.4 Missing Data 57
4.5 Interim Analyses and Data Monitoring 57
4.6 Multi-centre Studies 58
4.7 Multiple Testing 58
4.8 Estimand Framework 58
5 Summary of Study Data 59
5.1 Subject Disposition 59
5.2 Derived variables 59
5.3 Protocol deviations and violations 59
5.4 Baseline Demographic Variables 59
5.5 Treatment Compliance 60
6 Efficacy Analyses 61
6.1 Primary Efficacy Analysis 61

6.2 Secondary Efficacy Analyses 61
6.3 Estimand-to-analysis for primary and secondary objectives 62
6.4 Exploratory Efficacy Analyses 65
6.4.1 Complier average causal effect 65
6.4.2 Subgroup analyses 65
7 Safety Analyses 65
7.1 Adverse Events 65
7.2 Serious Adverse Events 66
8 Figures 66
9 Reporting Conventions 66
10 Technical Details 66
11 Summary of Changes to the Protocol 66
Appendix A – Table and Figure Listing for the Final Report 67
Appendix B – Templates for tables and figures 68
Appendix C – Data Mapping to Database 81

LIST OF ABBREVIATIONS

AE	Adverse Event
BFI	Brief Fatigue Inventory
CACE	Complier Average Causal Effects
CI	Confidence Interval
CONSORT	Consolidated Standards of Reporting Trials
COPD	Chronic Obstructive Pulmonary Disease
COST	COmprehensive Score for financial Toxicity
DLCO	Diffusing capacity of the lungs for carbon monoxide
ECOG-PS	Eastern Cooperate Oncology Group - Performance Status
EORTC-QLQ	European Organization for the Research and Treatment of Cancer - Quality of Life Questionnaire
FEV1	Forced Expiratory Volume in the first second
FVC	Forced Vital Capacity
HRQoL	Health-Related Quality of Life
ICH	International Council for Harmonisation
IPAQ-SF	International Physical Activity Questionnaire Short Form
LC	Lung Cancer
MAR	Missing At Random
MET	Metabolic Equivalent Tasks
NSCLC	Non-Small Cell Lung Cancer
PVD	Peripheral Vascular Disease
REDCap	Research Electronic Data Capture
SAE	Serious Adverse Event
SAP	Statistical Analysis Plan
SD	Standard Deviation
SPIRIT	Standard Protocol Items: Recommendations for Interventional Trials
SPPB	Short Physical Performance Battery
TIDieR	Template for Intervention Description and Replication
6MWD	6-Minute Walk Distance

1. INTRODUCTION

1.1 Preface

The majority of people with operable lung cancer now survive yet they suffer significant physical hardship. New models of care are required to minimise morbidity for this large and vulnerable group. This project will test the benefit of an exercise and education self-management program, compared to usual care, for people undergoing surgery for lung cancer to improve their physical function and functional recovery. The project will involve a planned sample of 112 people undergoing surgery for lung cancer in Victoria, Australia. Participants will be randomised before surgery to either the intervention arm (exercise and education self-management program) or the usual care (no exercise program) arm. Participants in both arms will receive usual medical, nursing and allied health care. In addition to usual care, participants in the intervention arm will receive an exercise and education self-management program. The program consists of one to two appointments with a physiotherapist (before hospital discharge post-operatively). Additionally, the patient will receive weekly phone calls up to 12 weeks after surgery to promote adherence to physical activity goals, discuss barriers/issues with their exercise and progress their exercise program. The intervention is designed to be equitable and inclusive of rural patients, as measurement and intervention appointments are timed with surgical consults and the additional intervention is phone-based. Participants in both arms will undergo a battery of tests with a blinded assessor including measurement of physical function, quality of life and physical activity prior to surgery, at hospital discharge, and at 12 weeks, 6 months and 12 months post-surgery. This study will provide important information on the benefit of an exercise-based intervention for people with lung cancer.

1.2 Purpose of the SAP

The purpose of this Statistical Analysis Plan (SAP) is to outline the pre-planned analyses to be completed to support the main publication of the CAPACITY trial. Versions of the SAP will be tracked until unblinding and thereafter, with a clear distinction between the changes before and after unblinding. Any analyses not identified in the SAP after breaking of the study blind will be clearly identified as such in the main publication and will be considered post-hoc. This SAP excludes the description of the analysis of the economic data (European Quality of Life Instrument, healthcare resource usage, cost of delivering intervention) collected as part of the study which will be documented separately.

2 Study Objectives and Endpoints

2.1 Study Objectives and Hypotheses

The primary objective of this study is:

- to evaluate the effect of a self-management program (exercise and education) on physical function at 3 months post-operative in patients with operable lung cancer compared with usual care (control).
  Hypothesis 1: The self-management program compared with usual care will improve physical function at three months after surgery.

The secondary objectives are:

- To assess the effect of a self-management program (exercise and education) on physical function, physical activity (levels and self-efficacy), muscle strength and function, health-related quality of life (HRQoL), fatigue, sleep, symptoms, distress, financial toxicity, survival and return to work/usual activities in patients with operable lung cancer compared with usual care (control).
  Hypothesis 2: The self-management program compared with usual care will improve physical function, physical activity (levels and self-efficacy), muscle strength and function, health-related quality of life (HRQoL), sleep and symptoms in patients with operable lung cancer at 3, 6 and 12 months after surgery.

- *To assess the effect of a self-management program (exercise and education) on health care resource usage and financial burden for patients with operable lung cancer at 12 months after surgery compared with usual care (control).
  Hypothesis 3: The self-management program compared with usual care will reduce health care resource usage (including hospitalisations, length of stay and intensive care admissions) in the first 12 months after surgery and reduce financial burden for patients.

- *To explore the cost effectiveness of a self-management program (exercise and education) for patients with operable lung cancer compared with usual care (control).
  Hypothesis 4: The self-management program will be cost-effective compared to current standard treatment in patients with operable lung cancer at 12 months after surgery.

**Note: Analyses for these objectives (hypotheses 3 and 4) will not be described in this SAP but will be documented in a separate analysis plan to be undertaken in the future*

2.2 Outcome measures

The primary outcome measure is the physical function domain score of the European Organization for the Research and Treatment of Cancer questionnaire (EORTC QLQ C30 version 3) at 3 months post-operatively.

The secondary outcome measures are listed below. Derivation of the measures, where applicable, will be provided in Section 3.5. Information on demographic and baseline variables can be found in Section 0.

- Physical function:
  - Physical function domain score of the EORTC QLQ-c30 at 6 and 12 months after surgery.
  - Short Physical Performance Battery (SPPB) overall and individual (gait, balance, and chair) scores at 3 and 6 months post-operatively
- Health-related quality of life and symptoms:
  - EORTC QLQ-C30 domain and single item scores at 3, 6 and 12 months post-operatively

- EORTC QLQ-LC13 domain and single item scores at 3, 6 and 12 months post-operatively
- Functional exercise capacity:
  - 6-minute walk distance at 3 and 6 months post-operatively
- Muscle strength:
  - Quadriceps strength test at 3 and 6 months post-operatively
  - Hand grip strength test at 3 and 6 months post-operatively
- Physical activity levels at 3 and 6 months post-operatively:
  - International Physical Activity Questionnaire short form (IPAQ-SF)
    - Total MET (minutes/week)
    - Categorical Score of IPAQ physical activity (high, moderate or low)
  - Sedentary activity at 3 and 6 months post-operatively
    - Television and video viewing time
- Self-efficacy for physical activity:
  - Barriers, task and walking self-efficacy scales at 3 and 6 months post-operatively
- Fatigue:
  - Brief fatigue inventory global score at 3 and 6 months post-operatively
- Distress:
  - Distress thermometer at 3 and 6 months post-operatively
- Sleep:
  - Sleep disturbance-short form 8b PROMIS Item Bank at 3 and 6 months post-operatively
- Financial toxicity:
  - COmprehensive score for financial toxicity (COST) at 3 and 6 months post-operatively
- Return to work for participants who had employment or non-paid volunteer work prior to surgery: Employment questionnaire adapted for lung cancer at 3, 6 and 12 months post-operatively
  - Current employment status
  - Time to return to work after hospital discharge
- Return to usual activities for participants who did not have employment in the 6 months prior to surgery: Employment questionnaire adapted for lung cancer at 3, 6 and 12 months post-operatively
  - Time to return to usual activities after hospital discharge
- Survival status at 12 months

2.3 Safety outcomes

Intervention participants will be asked during telehealth consultations to report any adverse events. These include:

- Serious adverse events are defined as any events which are life-threatening or result in death or hospitalisation (or prolongation of hospitalisation), incapacity or disability.
- Minor adverse events include new or progressive pain, non-injurious falls, severe dyspnoea, worsening fatigue, palpitations, neurological deficits and altered cognitive status.

2.4 Other outcomes

Data will be collected on the number and reason for hospital re-admissions in 12 months since surgery.

3 Study Methods

3.1 Study Design and Plan

This is a multisite, parallel-group, two-arm, phase II randomised controlled superiority trial with a 1:1 allocation ratio to either a 12-week programme of home-based exercise and self-management plus usual care (Program/intervention) or usual care alone (control). The study schema is shown in Figure 1. The protocol will follow the recommendations of Standard Protocol Items: Recommendations for Interventional Trials (SPIRIT), Guidelines for Reporting Trial Protocols and Completed Trials Modified Due to the COVID-19 Pandemic and Other Extenuating Circumstances CONSERVE-SPIRIT extension) and Template for Intervention Description and Replication (TIDieR). The total recruitment target is 112 participants.

Participants will be asked to participate in five testing sessions across the 12 month period of the study with the first testing session just prior to surgery and subsequent sessions scheduled: before hospital discharge, and at 3 months, 6 months and 12 months post surgery. Each testing session is anticipated to take a maximum of 90 minutes in total. Assessments will occur in the outpatient physiotherapy, thoracic surgery or respiratory medicine departments, however for participants unable to travel/drive to attend or those who prefer not to attend the hospital the hospital for follow-up appointments, will be offered a home visit assessment if they live within a feasible distance of the hospital.

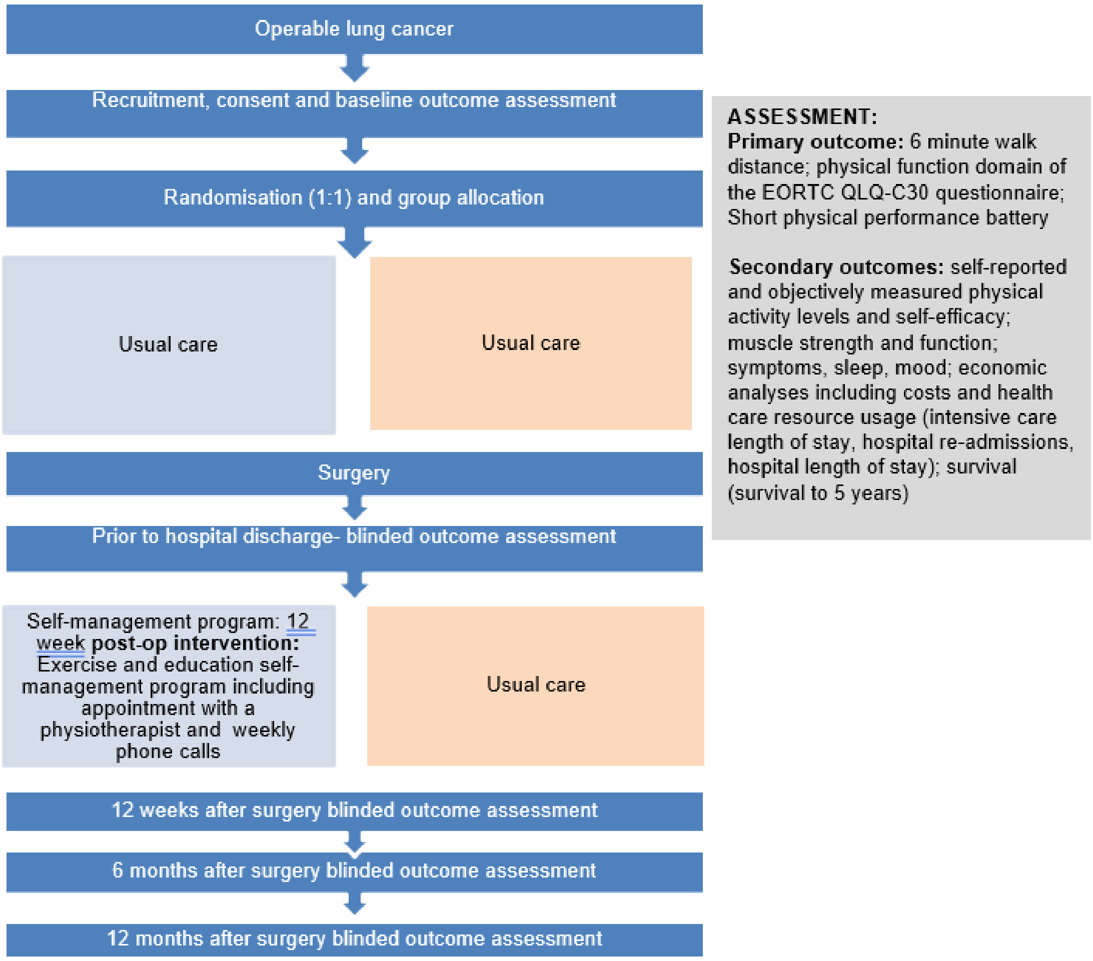

Figure 1: CAPACITY Trial schema

3.2 Intervention groups

The program consists of one to two appointments with a physiotherapist (before hospital discharge post-operatively). Additionally, the patient will receive weekly phone calls up to 12 weeks after surgery to promote adherence to physical activity goals, discuss barriers/issues with their exercise and progress their exercise program. The intervention is designed to be equitable and inclusive of rural patients, as measurement and intervention appointments are timed with surgical consults and the additional intervention is phone-based.

3.3 Inclusion-Exclusion Criteria

Eligible participants are adults, aged 18 years or over who meet all the following inclusion criteria:

i) Able to provide consent
ii) Planned to receive surgical treatment for non-small cell lung cancer
iii) Expected to be alive > 6 months
iv) Have surgeon or physician approval to participate in the study
v) Are not currently meeting the physical activity guidelines (150 minutes of moderate intensity physical activity per week)
vi) Have an Eastern Cooperate Oncology Group (ECOG) performance status of 0-2 at study entry
   - ECOG 0: Fully active
   - ECOG 1: Walking, but only can do light work
   - ECOG 2: Rest in bed LESS than half the day, do not work but can care for self

Participants will be deemed ineligible to participate if they satisfy any of the following exclusion criteria:

i) Are non-English speaking (insufficient English language skills to complete the questionnaires)
ii) Have metastatic disease (stage IV lung cancer) at study entry
iii) Have acute uncontrolled cardiovascular or respiratory issues
iv) Have decompensated heart failure, severe aortic stenosis, uncontrolled arrhythmia, or acute coronary syndrome
v) Are non-ambulant (for example: amputee, spinal cord injury, wheel-chair bound)
vi) Have an ECOG performance status of 3 or 4 at study entry
vii) Have cognitive impairment (determined as not being able to provide consent for surgery)

3.4 Randomisation and Blinding

Eligible participants were randomly allocated in a 1:1 ratio to either the exercise and education arm or to the usual care arm. Randomisation was stratified by hospital site (A – Royal Melbourne Hospital and B – St Vincent's Hospital Melbourne). The randomisation schedule was computer generated by an independent statistician and allocation was carried out centrally by the lead investigator or site trial coordinator using the Research Electronic Data Capture (REDCap) randomisation module. Block permuted randomisation with varying block sizes was used. Allocation concealment was achieved by only releasing the randomisation code after the participant completed the baseline outcome measures and had undergone surgery.

Randomisation occurred day 1 or 2 post surgery to ensure all patients had undergone surgery and received confirmation of a diagnosis of non-small cell lung cancer (NSCLC). If the histological diagnosis was still unconfirmed at this point, the patient was randomised and retained in the trial[1]. The final diagnoses will be reported at the end of the trial. Following randomisation, the intervention physiotherapist was contacted by the lead investigator or trial coordinators and informed of the group allocation. Blinding of participants and the intervention physiotherapists was not possible due to the nature of the exercise intervention. Outcome assessors were blinded to group allocation throughout all assessment periods. If an assessor became unblinded, it was documented and reported. All usual care staff who provide usual clinical care to patients on the ward were blinded to group allocation. Other study investigators (excluding those involved in delivering the intervention), including the study statistician, will remain blinded until the database has been cleaned, a blinded data review has taken place and the data are ready for analysis.

3.5 Sample Size

A sample size of 88 (44 per arm) was required to provide 80% power with a two-sided alpha of 0.05 to detect a minimum difference of 12.9 in mean EORTC QLQ-C30 physical function score (primary outcome) between the two arms at 3-months follow-up (primary time point), assuming a standard deviation of 21.3 (from our prior published data). To allow for an anticipated drop-out rate of 20% the sample size was increased to 112 participants (an additional 24 participants).

At the time of writing the SAP recruitment was completed with 116 participants randomised.

[1] However, if no patients no longer meet the eligibility criteria (Section 0) they will be deemed ineligible and excluded in the analysis population.

3.6 Study Visit Schedule

Study variables were collected according to the study visit schedule in Table 1. At the time of writing this SAP, recruitment was complete, and the study was in the follow-up period.

Table 1: Schedule of enrolment, intervention and assessments

**Assessment/ Procedure**	**Pre-surgery and before randomisation**	**Post-operatively before hospital discharge**	**3 months post-operatively**	**6 months post-operatively**	**12 months post-operatively**
**ENROLMENT:**					
Eligibility screen	X				
Informed Consent	X				
Allocation		X*			
Demographic and medical information, including surgical procedure	X	X			
**ASSESSMENTS:**					
EORTC QLQ-c30 and LC13 questionnaire	X		X**	X	X
Physical function test– 6MWD	X		X	X	
Physical function test –SPPB	X	X	X	X	
Muscle strength and function tests	X	X	X	X	
Physical activity – activity devices, and PA levels questionnaires	X		X	X	
Self-efficacy questionnaires	X	X	X	X	
Symptoms, sleep, fatigue, financial burden, return to work and mood questionnaires	X		X	X	X (return to work only)
Intervention satisfaction and knowledge questionnaires (intervention group only)	X		X		
EQ 5D 5L	X		X	X	X
Health care resource usage data over 12 months					X
Pre post surveys (views of program)	X		X		
Interviews with 15-20 participants				X	
Survival follow-up					x (and at 5 years)

*1 to 2 days post-op
**Timepoint of the primary endpoint (physical function score)

Time-windows will be applied to all visit data collected at baseline, 3-months, 6-months and 12-months, with assessments outside the predefined visit windows excluded from the analyses. Relative days inclusive of visit date will be derived as the visit date minus the randomisation date. The visit windows in Table 2 will be applied to the relative days.

Table 2: Assessment visit windows

Visit	Target day	Lower limit (incl.)	Upper limit (incl.)
Day -1 – Screening		Day -10	Day -1
Pre-surgery and before randomisation			
Day 0 – Randomisation date		Day 0	Day 0
Post-operatively before hospital discharge*			
Month 3 – 3 months post-operatively	Day 90	Day 62	Day 118
Month 6 – 6 months post-operatively	Day 180	Day 152	Day 208
Month 12 - 12 months post-operatively	Day 365	Day 323	Day 407

*Assessments at this visit are not included in this analysis

3.7 Outcome Variables

A detailed description of the efficacy study variables, grouped according to domain, is presented in Sections 0 to 0. For each outcome measure, details on the data type of variable, whether it will be derived and the variable name in the study database is provided in Table 9.

The patient-reported outcomes were obtained from the following questionnaires:

- The European Organization for the Research and Treatment of Cancer Quality of Life Questionnaires: core 30-item (EORTC QLQ-c30, version 3) and the Lung Cancer 13-item supplement (EORTC QLQ-LC13)
- The International Physical Activity Questionnaire Short Form (IPAQ-SF)
- Self-efficacy for physical activity
- Brief Fatigue Inventory (BFI)
- Distress Thermometer
- Sleep Disturbance-Short Form 8b PROMIS Item Bank V1.0
- COmprehensive Score for financial Toxicity (COST)

The objective outcomes were obtained from the following questionnaires or tests:

- Short Physical Performance Battery (SPPB)
- 6-minute walk distance test (6MWD)
- Quadriceps strength test
- Hand grip strength test

Published scoring manuals, if available, will be used if required to derive the outcomes for each questionnaire used in the study.

In the subsections that follow, some of the outcome variables are to be derived by the statistician performing the analysis. In most cases there is a questionnaire manual detailing how to compute composite scores if items are missing. If a questionnaire manual does not describe how to handle missing data for a questionnaire then the score will be coded as missing.

3.7.1 Physical function

Physical function will be measured via two methods: the EORTC QLQ-c30 instrument (primary endpoint) and the Short Physical Performance Battery (SPPB).

EORTC QLQ-c30

The physical function score from the EORTC QLQ-c30 will be derived from 5 items (Items 1 to 5) which are coded with response categories of: 1 - Not at all; 2 - A little; 3 - Quite a bit; and 4 - Very much. The responses to the 5 items are averaged to produce a raw score (RS, with range of 1 to 4) and then a linear transformation is applied to re-scale and produce a score (S) with a range of 0 to 100. A high physical function score indicates better function.

In general, the calculation of a functional scale from the QLQ-c30 is derived as follows:

1. Calculate the raw score (RS): $RS = (I_1 + I_2 + \cdots + I_n)/n$
2. Apply the linear transformation to 0-100 to obtain the score S,
   Functional scales: $S = \left\{1 - \frac{(RS-1)}{range}\right\} \times 100$

If at least 50% of the subscale items are missing, for the physical function scale this equates to any 3 of the 5 questions, then the physical function score will be treated as missing.

SPPB

The SPPB will produce 4 outcomes based on the following tests performed by participants:

1. Gait speed: participants are instructed to walk a distance of 4 meters and the average of two trials are used;
2. Standing balance: participants are assessed in three different static positions (side-by-side stand, semi-tandem stand and tandem stand) for 10 seconds each, and
3. Chair rise task: participants are instructed to stand up and sit down five times in a row as quickly as possible.
4. Overall SPPB performance score

Each individual test is scored on a scale of zero to four points with higher scores indicating better performance. The three test scores are summated to give an overall SPPB performance score ranging from zero to 12 points. A zero score indicates poor function whilst 12 indicates excellent function. It has been previously reported in the literature that for older adults a score of 10 is considered the cut-off for mobility impairment (i.e. scores <10 = poor mobility) and will be used to guide interpretation of results (not as an outcome).

If a participant attends the visit but was not physically able to complete a test, a score of 0 is given for that test. If a participant did not attend or refuses to do the test, the score is treated as missing.

3.7.2 Health-related quality of life and symptoms

Both the EORTC QLQ-c30 and EORTC QLQ-LC13 questionnaires will be used to assess health-related quality of life and symptoms.

EORTC QLQ-c30

Health-related quality of life and symptoms outcomes will be derived using scales from the EORTC QLQ-c30. The EORTC QLQ-c30 generates 9 multi-item scales: 5 functional scales (physical, role, emotional, cognitive and social functioning), 3 symptom scales (pain, fatigue and nausea/vomiting) and a global health status/quality of life scale and six single-item scales (appetite loss, dyspnoea, diarrhea, constipation, insomnia and financial impact). The items used to generate each scale are summarised in Table 3. The first 28 items are functional and symptom scales coded with response categories: 1 - Not at all; 2 - A little; 3 - Quite a bit; and 4 - Very much. The last two items measuring global health status/QoL are recorded on a Likert scale: 1 - very poor to 7 - excellent.

Table 3 The EORTC QLQ C30 version 3.0 scales and items

	Scale	Number of items	Item range*	**Version 3.0** Item numbers	Function scales
**Global health status / QoL**					
Global health status/QoL (revised)†	QL2	2	6	29, 30	
**Functional scales**					
Physical functioning (revised)†	PF2	5	3	1 to 5	F
Role functioning (revised)†	RF2	2	3	6, 7	F
Emotional functioning	EF	4	3	21 to 24	F
Cognitive functioning	CF	2	3	20, 25	F
Social functioning	SF	2	3	26, 27	F
**Symptom scales / items**					
Fatigue	FA	3	3	10, 12, 18	
Nausea and vomiting	NV	2	3	14, 15	
Pain	PA	2	3	9, 19	
Dyspnoea	DY	1	3	8	
Insomnia	SL	1	3	11	
Appetite loss	AP	1	3	13	
Constipation	CO	1	3	16	
Diarrhoea	DI	1	3	17	
Financial difficulties	FI	1	3	28	

* *Item range* is the difference between the possible maximum and the minimum response to individual items; most items take values from 1 to 4, giving *range* = 3.
† (revised) scales are those that have been changed since version 1.0, and their short names are indicated in this manual by a suffix "2" – for example, PF2.

The EORTC QLQ-c30 scales have not been computed within the study database so will be derived according to the EORTC QLQ-c30 scoring manual[2]. All the scales and single-item measures are linearly transformed to a scale of zero to 100. Higher scores on functional domains and global health status/quality of life scale represent higher functioning and higher HRQoL. Lower scores on symptom domains and single items represent less symptoms.

[2] https://www.eortc.org/app/uploads/sites/2/2018/02/SCmanual.pdf

Calculation of EORTC QLQ-c30 scales

The principle for scoring these scales is the same in all cases:

1. Estimate the average of the items that contribute to the scale; this is the raw score.
2. Use a linear transformation to standardise the raw score, so that scores range from 0 to 100; a higher score represents a higher ("better") level of functioning, or a higher ("worse") level of symptoms.

In practical terms, if items $I_1 + I_2 + \cdots + I_n$ are included in a scale, the procedure is as follows:

3. Calculate the raw score (RS): $RS = (I_1 + I_2 + \cdots + I_n)/n$
4. Apply the linear transformation to 0-100 to obtain the score S,

   Functional scales: $S = \left\{1 - \frac{(RS-1)}{range}\right\} \times 100$

   Symptom scales / items: $S = \left\{\frac{(RS-1)}{range}\right\} \times 100$

   Global health status / QoL: $S = \left\{\frac{(RS-1)}{range}\right\} \times 100$

The range is the difference between the maximum possible value of RS and the minimum possible value. The QLQ-c30 has been designed so that all items in any scale take the same range of values. Therefore, the range of RS equals the range of the item values. Most items are scored 1 to 4, giving range = 3. The exceptions are the items contributing to the global health status/QoL, which are 7-point questions with a range of 6.

If at least 50% of the subscale items are missing, then the subscale will be treated as missing. Missing single items will also be treated as missing. No multiple imputation will be used.

The Stata command "qlqc30[3]" will be used to perform the scoring of the EORTC QLQ-c30. The code for this has been checked by the SAP author (DZ) to ensure the correct derivation and handling of missing data.

EORTC QLQ-LC13 lung cancer supplement module

Health-related quality of life and symptoms outcomes specific to lung cancer will be derived using scales from the EORTC QLQ-LC13 module. The QLQ-LC13 includes questions assessing lung cancer-associated symptoms (cough, haemoptysis, dyspnoea and site-specific pain) and treatment-related side effects (sore mouth, dysphagia, peripheral neuropathy and alopecia). The items used to generate each scale are summarised in Table 4.

The scoring approach for the QLQ-LC13 is identical in principle to that for the symptom scales/single items of the QLQ-c30 with missing data handled the same way as described above. The only exception to this is when deriving the "Dyspnoea" scale where if item 5 is missing then item 3 and item 4 should be used as single-items as not to introduce bias in the measure.

The Stata command "qlqlc13" will be used to perform the scoring of the EORTC QLQ-LC13. This command has been created by the SAP author (DZ) using the "qlqc30" command as template.

[3] Bascoul-Mollevi, C., Castan, F., Azria, D., & Gourgou-Bourgade, S. (2015). EORTC QLQ-C30 Descriptive Analysis with the qlqc30 Command. The Stata Journal, 15(4), 1060–1074.

Table 4 The EORTC QLQ-LC13 scales and items

Scale name	Scale	Number of items	Item range*	QLQ-LC13 Item numbers	†
**Symptom scales / items**					
Dyspnoea†	LCDY	3†	3	3,4,5	X
Coughing	LCCO	1	3	1	
Haemoptysis	LCHA	1	3	2	
Sore mouth	LCSM	1	3	6	
Dysphagia	LCDS	1	3	7	
Peripheral neuropathy	LCPN	1	3	8	
Alopecia	LCHR	1	3	9	
Pain in chest	LCPC	1	3	10	
Pain in arm or shoulder	LCPA	1	3	11	
Pain in other parts	LCPO	1	3	12	

* "Item range" is the difference between the possible maximum and the minimum response to individual items.
† The dyspnoea scale should only be used if all three items have been answered. Some respondents ignore question 5 because they never climb stairs; in this case, the score for the dyspnoea scale would be biased if it were based upon the other two items. Hence if item 5 is missing then items 3 and 4 should be used as single-item measures.

3.7.3 Functional exercise capacity

Functional exercise capacity will be measured using the 6-minute walk distance (6MWD)

<u>6-minute walk distance functional exercise capacity</u>

The 6-minute walk distance (6MWD) is a field walking test performed according to the American Thoracic Society guidelines[4]. Participants are asked to walk up and down a 30m flat straight corridor and cover as much ground as possible in six minutes. The American Thoracic Society guideline recommends using the best of two repeated 6MWD. The resulting outcome is a distance with a range from 0 meters to 800 meters. If the participant has only one attempt then this will the outcome measure. If a participant attends the visit but was not able to complete or attempt a test then a score of 0 is given for that test. If a participant declined the test or did not attend the visit then each score is treated as missing.

3.7.4 Muscle strength

Muscle strength will be measured using four outcomes derived from two tests: quadriceps strength test (left and right) and the hand grip strength test (left and right).

<u>Quadriceps strength test</u>

Quadriceps muscle strength is measured by two quantities:

i) highest peak force (kilograms) and
ii) longest time (seconds) to peak force over 6s.

The test is repeated three times for each participant on each leg after a practice bilaterally. The highest peak force (kilograms) and longest time to peak force over 6s out of the 6 trials conducted on each leg (3x left quadricep, 3x right quadricep) will be analysed. If only one leg has been tested, this result will still be used. If a test is only performed once, then the single measure obtained will be used in the analysis.

<u>Hand grip strength test</u>

4 American Thoracic Society, ATS statement: guidelines for the six-minute walk test. American Journal of Respiratory and Critical Care Medicine, 2002. 166: p. 111-117.

Hand grip strength test is measured by two quantities:

i) highest peak force (kilograms) and

ii) longest time (seconds) to peak force over 6s.

The test is repeated three times for each participant on each hand after a practice bilaterally. The highest peak force (kilograms) and longest time to peak force over 6s out of the 6 trials conducted on each hand (3x left hand, 3x right hand) will be analysed. If only one hand has been tested, this result will still be used.

If a participant attends the visit but did not complete or attempt a test then a score of 0 is given for that test. If a participant did not attend the visit then each score is treated as missing.

3.7.5 Physical activity levels

International Physical Activity Questionnaire- Short Form

Physical activity will be measured using two outcomes derived from the International Physical Activity Questionnaire Short Form (IPAQ-SF), a 7-item questionnaire asking participants to recall their physical activity from the past seven days. The first 6 items (number of minutes and days spent on walking, moderate and vigorous activity) are used to calculate the first outcome of total physical activity Metabolic Equivalent Tasks (MET)-minutes/week. This is derived by adding up the calculated MET-minutes within each physical activity intensity level: vigorous intensity, moderate intensity, and walking.

Calculation of Total physical activity MET-minutes/week

The calculation of the score* is:

1. Walking MET-minutes/week = 3.3 x walking minutes x walking days
2. Moderate MET-minutes/week = 4.0 x moderate-intensity activity minutes x moderate days
3. Vigorous MET-minutes/week = 8.0 x vigorous-intensity activity minutes x vigorous-intensity days
4. Total physical activity MET-minutes/week = sum of Walking (i)+ Moderate (ii) + Vigorous MET-minutes/week scores (iii)

* As per the IPAQ-SF guidelines[5], all walking, moderate and vigorous activity time variables exceeding 180 minutes will be truncated (that is re-coded) to be equal to 180 minutes. This rule permits a maximum of 21 hours of activity in a week to be reported for each category (3 hours * 7 days). However, if the sum of all walking, moderate and vigorous time variables exceeds 960 minutes (16 hours) then this should be excluded from the analysis[6]. Also, only values of 10 or more minutes of activity should be included in the calculation of the IPAQ-SF MET-minutes/week scores.

Calculation of categorised physical activity

The second outcome is a categorisation of the MET-minutes/week scores into three groups according to their physical activity levels:

i. "High" physical activity level - meeting any one of the following criteria: vigorous-intensity activity on ≥3 days and accumulating ≥1500 MET-min/week, or ≥7days of any activity accumulating ≥3000 MET-min/week);
ii. "Moderate" physical activity level - meeting any one of the following criteria: ≥3 days of vigorous activity of ≥20 min/day; or ≥5 days of moderate-intensity activity and/or walking ≥30 min; or ≥5 days of activity accumulating ≥600 MET-min/ week);
iii. "Low" physical activity level - no physical activity reported or some activity reported but not satisfying neither high or moderate criteria.

If data are missing in walking, moderate or vigorous days or minutes or a response of "Don't know/Not sure" then the data is set as missing.

[5] https://www.physio-pedia.com/images/c/c7/Quidelines_for_interpreting_the_IPAQ.pdf

[6] Assumes than on average an individual sleeps 8 hours per day

Sedentary behaviour

Sedentary behaviour will be assessed via the question “On average over the last week, how many hours per day did you watch television or videos?”. The outcome is a self-reported average number of hours per day with no derivation required.

3.7.6 Self-efficacy for physical activity

Self-efficacy for physical activity will be measured by three outcomes based on a questionnaire concerning barriers, task and walking self-efficacy where participants had to indicate how confident they felt carrying out exercise in certain situations. This included a 9-item barrier self-efficacy scale, a 4-item task self-efficacy scale, and a 6-item walking self-efficacy scale. Items were rated on a scale from 0% “Not at all confident” to 100% “Extremely confident” in 10% increments.

Calculation of self-efficacy scores

Overall scores for each scale (barriers, task and walking) need to be derived by averaging the scores from the items within each scale[7]:

1. The barrier self-efficacy overall score
   - Six or more items out of the 9-item barrier self-efficacy scales must be completed to calculate this score. The arithmetic mean is taken across the completed items to give a range of scores from 0% to 100%.
   - If more than 3 items are missing, then the barrier score is treated as missing.
2. The task self-efficacy overall score
   - Three or more items out of the 4-item task self-efficacy scales must be completed to calculate this score. The arithmetic mean is taken across the completed items to give a range of scores from 0% to 100%.
   - If more than 1 item is missing, then the task score is treated as missing.
3. The walking self-efficacy overall score
   - Four or more items out of the 6-item walking self-efficacy scales must be completed to calculate this score. The arithmetic mean is taken across the completed items to give a range of scores from 0% to 100%.
   - If more than 2 items are missing, then the walking score is treated as missing.

3.7.7 Fatigue

Fatigue will be measured using the Brief Fatigue Inventory (BFI) to derive the outcome global BFI score. The BFI contains 9 items assessing the severity of fatigue and the impact of fatigue on daily function in patients with cancer and asks about fatigue over the past 24 hours.

To calculate the global BFI score at least five of the nine BFI items must be completed to calculate this score. The arithmetic mean is calculated across the completed items to give a range of scores from 0 to 10. If more than 4 items are missing, then the global BFI score is treated as missing.

3.7.8 Distress

The Distress Thermometer will be used to measure distress. It is a visual analogue scale with scores from 0 (“no distress”) to 10 (“extreme distress”) with a midpoint anchor of 5 labelled as “moderate distress” (11). No derivation will be required for this score as it is a single item.

---

[7] Due to a scoring manual being unavailable for this questionnaire, the approach to deal with missing data is based on the paper by Ritter and Loring (Journal of clinical epidemiology 2014: 67:1265-1273) which used a self-efficacy questionnaire for physical activity that had scales based on 4 and 6 items and in their methods they stated how many non-missing responses were required to complete the scale. This was extrapolated for the 9-item scale.

3.7.9 Sleep

Sleep disturbance will be measured using the sleep disturbance-short form 8b PROMIS item bank 8-item Sleep Disturbance – Short Form 8b (12). Each item is rated on a 5-point scale (1=never; 2=rarely; 3=sometimes; 4=often; and 5=always). The resulting sleep score, a T-score, will be derived as per the scoring manual[8] as described below:

Calculation of T-score

1. Due to the coding format in the CAPACITY REDCap database, the following items need to be reversed so that the correct summation can occur: Items 2,3,7 and 8 will be recoded by subtracting the response from 6 so that 1 becomes 5, 2 becomes 4, 3 stays as 3, 4 becomes 2 and 5 becomes 1.

2. If *all 8 items have a response*, the total raw score is calculated by summing the 8 responses resulting in a score ranging from 8 to 40 with higher scores indicating greater severity of sleep disturbance.

3. If *at least 4 items have a response*, the total raw score is calculated by summing the response scores from the items that were answered. Multiply this sum by the total number of items in the short form then divide by the number of items that were answered. If the result is a fraction, round up to the nearest whole number. This is a \ed total raw score. For pro-rated scores, this calculation assumes that responses are missing at random.

4. If *at least 4 items do not have a response* then a total raw score cannot be computed and is treated as missing.

5. The total raw score is then converted to a T-score using Table 5. The T-score rescales the raw score into a standardized score with a mean of 50 and a standard deviation (SD) of 10. Therefore a person with a T-score of 40 is one SD below the mean.

Table 5: PROMIS 8b short form conversion table

Sleep Disturbance 8b
Short Form Conversion Table

Raw Score	T-score	SE*
8	28.9	4.8
9	33.1	3.7
10	35.9	3.3
11	38.0	3.0
12	39.8	2.9
13	41.4	2.8
14	42.9	2.7
15	44.2	2.7
16	45.5	2.6
17	46.7	2.6
18	47.9	2.6
19	49.0	2.6
20	50.1	2.5
21	51.2	2.5
22	52.2	2.5
23	53.3	2.5
24	54.3	2.5
25	55.3	2.5
26	56.3	2.5
27	57.3	2.5
28	58.3	2.5
29	59.4	2.5
30	60.4	2.5
31	61.5	2.5
32	62.6	2.5
33	63.7	2.6
34	64.9	2.6
35	66.1	2.7
36	67.5	2.8
37	69.0	3.0
38	70.8	3.2
39	73.0	3.5
40	76.5	4.4

*SE = Standard Error on T-score metric

3.7.10 Financial toxicity

Financial toxicity will be measured using the COmprehensive Score for financial Toxicity (COST) questionnaire to derive the single outcome financial toxicity score based on 11 items.

Calculation of the financial toxicity score

1. Negatively stated-items 2,3,4,5,8,9 and 10 will be recoded by subtracting the response from 4 so that 0 becomes 4, 1 becomes 3, 2 stays as 2, 3 becomes 1 and 4 becomes 0.
2. If *all 11 items have a response* then the total raw score can be calculated by summing the 11 responses to obtain a score ranging from 0 to 44 with higher scores indicating better financial well-being.
3. If *at least 6 items have a response*, the total raw score is calculated by summing the response scores from the items that were answered. Multiply this sum by the total number of items in

[8] https://staging.healthmeasures.net/images/PROMIS/manuals/PROMIS_Sleep_Disturbance_Scoring_Manual.pdf

the short form then divide by the number of items that were answered. This is a pro-rated total raw score. For pro-rated scores, this calculation assumes that responses are missing at random.

4. If *at least 5 items do not have a response* then a total raw score cannot be computed and is treated as missing.

3.7.11 Return to work/Return to usual activities

This objective will be reported differently depending on the participants employment status. Selected questions from the Employment questionnaire adapted for this trial specific to the lung cancer surgery context[9].

For participants who were working (including non-paid volunteer work) prior to their surgery, the following two questions will be used to calculate the number of weeks they record at follow-up to first return to work:

i) Have you worked at all since you left the hospital? (question 7)
ii) How many weeks after hospital discharge did you return to work? (question 8)

For participants who were not working 6 months prior to their surgery, the following question will be used to calculate the number of weeks they record at follow-up to first resume usual activity:

i) If you were not working in the 6 months before you were in the hospital, how many weeks after hospital discharge did you return to the most important activity you did before being admitted to the hospital (e.g., gardening or attending religious service)? (question 5)

3.7.12 Survival

Survival status will be determined for all participants at 12 months post-surgery.

3.7.13 Hospital re-admissions

Data will be collected on the number of hospital re-admissions in 12 months since surgery. No derivation is required.

3.7.14 Outcomes not being covered in the main study analysis

There are secondary outcomes pertaining to muscle strength and physical activity levels that were included in the study protocol that will not be considered in the main analysis of the CAPACITY study. However, it is possible that an analysis of them may be undertaken in the future at the discretion of the CAPACITY study investigators.

The measures that will not be analysed and presented are:

i) Quadriceps muscle mass and quality (Muscle strength)
ii) Respironics ActiCal Z Watch accelerometer measures (Physical activity levels)
iii) Rating against PA guidelines for people with cancer (Physical activity levels)
iv) Physical Activity Scale for the Elderly (Physical activity levels)

4 GENERAL CONSIDERATIONS

4.1 Timing of Final Analysis

Blinded study data entered and stored in the REDCap study database hosted by the University of Melbourne will be transferred to the trial biostatistician located at the Centre for Epidemiology and Biostatistics at the University of Melbourne, Melbourne, Australia. After the last participant has concluded the 12-month study participation, all study data are available and have been cleaned, a blinded review meeting will be held with the CAPACITY study team and study biostatisticians (Karen

[9] The questionnaire was created by Dale M. Needham, MD, PhD and the Johns Hopkins University Outcomes After Critical Illness & Surgery (OACIS) Group, is licensed under the Creative Commons Attribution-NonCommercial-ShareAlike 4.0 International License. It has been approved for adaption and use in the trial.

Lamb, Diana Zannino) prior to database lock (i.e., it is locked so that data cannot be subsequently amended). During this masked data review meeting, the following topics will be discussed and decided upon without knowledge of the underlying treatment code:

1. Participants who have withdrawn consent, in relation to the use of the participant's data (or part of it) in any of the analyses (Section 0).
2. Participant's inclusion or exclusion status with regards to the "as randomised" and "as treated" treatment group (e.g., in the case of misallocation). The exact process for assigning the statuses will be defined and documented in the minutes of the meeting prior to breaking the mask along with the reason for classifying dyad to "as randomised" and "as treated".
3. Participant's overall adherence to study intervention as defined in Section 0, in relation to the use of the participant's data (or part of it) in the per protocol population (Section 0) and the safety population (Section 0). Overall adherence will be listed and tabulated for the purpose of the blinded data review meeting.
4. Participants with (minor or major) protocol violations as defined in Section 0, in relation to the use of the participant's data (or part of it) in the per protocol population (Section 0). Participants with (minor/major) protocol violations will be listed and tabulated for the purpose of the blinded data review meeting.
5. Participant's inclusion or exclusion status with regards to each analysis population (Section 0) guided by items 1-4 listed previously. The exact process for assigning the statuses will be defined and documented in the minutes of the meeting prior to breaking the blind along with any predefined reasons for removing a participant (or part of its data) from a particular population.

After database lock, the random code will be obtained from the independent statistician who generated the randomisation code and added to the study database by the trial statistician. No database may be locked, random code unmasked, or analyses completed until the SAP has been approved. The planned analyses in this SAP will be conducted after unmasking of the database and any changes to this SAP after unmasking will be documented in an amendment of this SAP and considered as post-hoc analyses.

The expected timing for data review and database lock is August 2023 and the analyses described in this SAP are expected to be performed by December 2023.

4.2 Analysis Populations

The following analysis populations are planned. The status of each participant with regards to the populations and "as randomised"/ "as treated" treatment group will be finalised during the blinded data review meeting.

4.2.1 Full Analysis/Randomised Population

This will consist of all participants who were randomised, excluding participants who have withdrawn from the study, including use of all existing data collected to withdrawal date, as documented in the minutes of the blinded data review meeting. Participants will be reported and analysed according to their randomised study arm ("as randomised"). This population will be used in the analysis of the effectiveness study variables. This will be the intention-to-treat population.

4.2.2 Per Protocol Population

This will consist of all participants who were randomised and had confirmed histological diagnosis of lung cancer. This population will be used as the secondary analysis set for the efficacy analyses on primary and secondary endpoints as described in Section 3.7. This population will also be used for the exploratory effectiveness analysis, where a complier average causal effect analysis will be undertaken to determine the effect among those that receive the intervention according to the protocol, considering adherence to the intervention as defined in Section 0.

4.2.3 Safety Population

This will consist of all participants in the intervention arm who completed at least one exercise session.

4.3 Covariates and Subgroups

4.3.1 Covariates

The models used for all study variables will be adjusted for the stratification variable, participating study site (i.e., Royal Melbourne Hospital and St Vincent’s Hospital).

4.3.2 Subgroups

An exploratory subgroup analysis based on postoperative cancer treatment will be performed for the primary outcome at 3-months post-randomisation using the full analysis population. The subgroup analysis listed below will be performed irrespective of whether the primary objective of the study based on EORTC-QLQ physical function at 3 months post-randomisation was achieved or not. The subgroups are:

- Postoperative cancer treatment: postoperative treatment/postoperative chemotherapy, radiotherapy or chemoradiotherapy
- No postoperative cancer treatment: postoperative treatment/postoperative chemotherapy, radiotherapy or chemoradiotherapy

4.4 Missing Data

To describe the missing data, the frequency and percentage of participants with a missing value at baseline, 3 months post-randomisation, 6 months post-randomisation and 12 months post-randomisation will be summarised for each of the study variables in Section 0 overall and by treatment arm (program, usual care) for the full analysis population. Where available, reasons for the missingness will be tabulated. In addition, baseline and demographic characteristics (Section 0) will be summarised overall and for those with and without a missing value at baseline, 3 months post-, 6 months post-randomisation and 12 months post-randomisation separately to examine if any characteristics appear to be associated with the presence or absence of data.

As the primary strategy to handle missing data, the analysis of continuous study variables will use a likelihood-based approach (i.e., constrained longitudinal data analysis, see Section 0). This approach assumes that the probability of missing data on the study variable is not related to the missing data but to some of the observed measured data in the model. That is, it assumes that the data are Missing at Random (MAR).

4.5 Interim Analyses and Data Monitoring

There were no interim analyses planned nor has an unplanned interim analysis occurred for this study. There was no formal Data and Safety Review Committee however, data quality reviews were performed by the principal and senior investigators.

4.6 Multi-centre Studies

This was a multi-centre study recruiting from 2 sites. The randomisation schedule was stratified by site. The primary and secondary study variables will be analysed for all sites combined with adjustment for site in the models. Due to a small recruitment number at one of the sites[10] an exploratory subgroup analyses will not be performed.

4.7 Multiple Testing

The comparison of the primary outcome (physical function) between self-management program (exercise and education) compared to usual care at 3 months will be performed at the 5% significance level. All secondary outcomes are exploratory and were not powered for. Therefore, no adjustment for multiple secondary outcomes will be adopted. Instead, all effect sizes and corresponding 95% confidence intervals (CIs) will be reported to let readers use their own judgement about the relative weight of the conclusions on the effect of the interventions on the secondary outcomes. This approach aligns with the usage of p-values favoured by the American Statistical Association. This approach will also be used for exploratory subgroup analyses.

4.8 Estimand Framework

The analysis of the study outcomes will follow the estimand framework (ICH E9(R1)) which involves specifying the following attributes of the estimand[11]:

i) Population of interest
ii) Variable (or endpoint) of interest
iii) Treatment description
iv) Handling of intercurrent event(s)[12]
v) Population level summary for the variable

There is only one intercurrent event within this trial which is death due to any cause. To deal with this intercurrent event the While-alive/while on treatment strategy will be adopted for all the estimands for the primary and secondary outcomes as outlined in Section 0. This involves using measurements up until the time of the intercurrent event which censors measurements after the intercurrent event.

---

[10] St Vincent's hospital recruited 12 participants, compared to Royal Melbourne hospital with 107 participants.

[11] An estimand is a precise description of the treatment effect which reflects the clinical question posed by a clinical trial objective.

[12] An intercurrent event is one that occurs after randomisation and prior to observation of the trial endpoints that affect either the interpretation or the existence of the measurements associated with the clinical question of interest.

5 Summary of Study Data

Participant data will be analysed and presented according to their randomised treatment allocation. Outcomes will be summarised using frequencies and percentages (based on the non-missing sample size) for categorical/binary variables, mean and standard deviation for continuous variables, or median and quartiles (25th and 75th percentile) for non-symmetrical continuous variables as assessed through graphical displays (histograms and/or boxplots).

Please refer to Appendices A and B for a listing tables and figures and corresponding templates for the reporting of study data. Appendix C provides data mapping to the study database (i.e.., the name of the variables to be used).

5.1 Subject Disposition

The flow of participants will be presented in a Consolidated Standards of Reporting Trials (CONSORT) diagram (Figure 1) and a summary of subject disposition during the study period (up to 12 months post-surgery) (Table 1.1) as presented in Appendix C.

The following will be included:

- Number of participants assessed for eligibility
- Number of participants not meeting the inclusion criteria and declined to participate
- Number of participants allocated to intervention group or usual care
- Number of participants completing visits at 3 months, 6 months and 12 months and number of participants who withdrew from the study and lost to follow-up
- Number of participants dropped out and for what reasons (death, toxicity, treatment failure, withdrew consent)
- Number of participants available for analysis for each of the primary, secondary and safety outcomes.

5.2 Derived variables

Any primary or secondary outcome variables requiring derivation are described in Section 0 and are indicated in the data mapping plan in Appendix C.

5.3 Protocol deviations and violations

Protocol deviations and violations will not be provided as the study protocol did not specify what constituted a protocol deviation or violation.

5.4 Baseline Demographic Variables

The summary statistics will be produced for the following demographic and baseline variables in accordance with Section 0.

Baseline demographics

- Age (in years) at surgery
- Sex (Female/Male)
- CAPACITY study site (Royal Melbourne Hospital/St Vincent's Hospital Melbourne)
- ECOG performance status
- Cancer histological type
- Cancer stage
- Previous cancer diagnosis (including lung)
- Comorbidities (diabetes, tobacco consumption, renal insufficiency, respiratory (COPD), cardiovascular (PVD), neoplastic, alcoholism→ Colinet comorbidity Score)
- Race/ethnicity
- Body mass index

- Smoking history
- Respiratory function (FEV1, FEV1/FVC, DLCO actual, DLCO percent predicted)
- Highest Education
- Occupational status
- Living arrangement (pre-admission)

Surgical and hospital details

- Type of surgery
- Hospital length of stay
- Any postoperative treatment
- ICU admission, length of stay, intubation
- Mobility status (use of gait aid, level of independence, endurance)

Post-operative therapy

- If chemotherapy or radiotherapy received

5.5 Treatment Compliance

During the weekly telehealth consultations, participants in the intervention group will be asked to recall their adherence rate to the exercise programme and report their daily step count.

Adherence rate will be reported as the percentage of consultations delivered over the 12 weeks (number delivered against target of 12 sessions). Completion rate will be reported as the percentage of participants who continue consultations to 12 weeks.

6 Efficacy Analyses

In general, continuous primary and secondary outcomes where there was a scheduled baseline visit and at least 1 post-surgery time points (3, 6 and/or 12 months) will be analysed using a likelihood-based longitudinal data analysis model incorporating all available outcome data as the response. Each model will include the study group allocation, time point and a study group by time point interaction and recruitment site. The absolute difference in mean change in the outcome score from baseline between the two study groups will be estimated at each post-surgery timepoint and reported with corresponding 95% confidence intervals (CI).

In addition to the analysis of participants according to their allocated group, the complier average causal effect will be estimated in secondary analyses, using collected adherence data. Heterogeneity of the intervention effect according to postoperative cancer treatment (no postoperative treatment/postoperative chemotherapy, radiotherapy or chemoradiotherapy) for the primary outcome will be assessed in exploratory analyses by including interactions between postoperative treatment and study group.

Further details on the analysis approach for the other outcomes not covered above are provided in section 0.

6.1 Primary Efficacy Analysis

The primary outcome (physical function domain total score of the EORTC QLQ C30) will be analysed using a likelihood-based longitudinal data analysis model, with response consisting of all scores (baseline, 3 months, 6 months and 12 months after surgery). The model will include factors representing study group, time point and a study group by time point interaction and recruitment site included in the model. The model will be restricted to have a common baseline mean score across the study groups (enforced statistically in the statistical model) based on the assumption that there are no differences in the mean outcome between groups at baseline due to randomisation, thus assuming the randomisation was effective. The constrained longitudinal data analysis model provides valid inference if the missing data mechanism for the outcome is at most missing at random.

The absolute difference in mean change in EORTC QLQ c30 physical function domain from baseline between groups will be estimated (including corresponding two-sided 95% CI and two-sided *p*-value) at 3 months after surgery (primary time point).

6.2 Secondary Efficacy Analyses

Secondary continuous outcomes will be analysed using a likelihood-based longitudinal data analysis model as per the primary outcome analysis. The model will include factors representing study group, time point and a study group by time point interaction and recruitment site included in the model. Results will be expressed as mean difference for the groups at 3 months, 6 months and 12 months post-randomisation where applicable, including two-sided 95% CI.

The secondary categorical outcome, IPAQ physical activity with High, Moderate, Low responses, will be analysed using a likelihood-based longitudinal multinominal logistic regression model with a multinomial logit link function. The model will include factors representing study group, time point and a study group by time point interaction and recruitment site included in the model. Results will be expressed as a Relative Risk Ratio for the relative comparison of the risk of the outcome (with "Low" as the reference category) compared between study groups at 3 months and 6 months post-randomisation where applicable, including two-sided 95% CI. If the frequencies of the IPAQ physical activity categories are low (e.g., less than 10 in a category) then consideration will be made to combine categories to form a binary response (e.g., Low/Moderate vs High or Low vs Moderate/High) and will be analysed using a likelihood-based longitudinal data analysis model with a logistic link function. The model will include factors representing study group, time point and a study group by

time point interaction and recruitment site included in the model. Results will be expressed as an Odds Ratio for the relative comparison of the odds of the outcome compared between study groups at 3 months and 6 months post-randomisation where applicable, including two-sided 95% CI.

The Kaplan-Meier method will be used to estimate the survival probability at 12 months post-surgery in each arm. Follow-up time will be measured from the date of operation until the date of death from any cause or date last known alive. Participants who have not died by the study closeout date will be censored. Participants who withdraw or are lost to follow-up before the closeout date will be censored at the date they are last known to be alive. Survival probability at 12 months will be reported for each study arm. A Cox proportional hazard model will be used to estimate the hazard ratio (HR) and corresponding 95% CI for time to death between the arms with the control group as the reference.

To estimate the mean time in weeks to return to work or usual activity, as separate analyses based on the employment status of the participant 6 months prior to their surgery, an accelerated failure time survival model will be used to obtain an estimate of the mean difference between the arms with corresponding 95% CI.

For continuous secondary outcomes measured at more than 2 timepoints, differences between randomised groups over time will be presented graphically with the mean parameter value at each timepoint for each treatment group with corresponding 95% confidence intervals.

p-values for the secondary outcomes will not be presented.

6.3 Estimand-to-analysis for primary and secondary objectives

The estimands concerning the primary objective and secondary objectives are described in Table 6 and Table 7. The handling of the intercurrent events and missing data will adopt the same strategy as per Table 6 as well as the analysis approach which is further described in Section 0.

Table 6: Estimand-to-analysis for the primary endpoint

<table>
<tr><td colspan="2">Objective: To evaluate the effect of a self-management program on physical function at 3 months post-operative in patients with operable lung cancer compared with usual care</td></tr>
<tr><td colspan="2">Estimand: The difference in mean physical function score (as measured with the EORTC-QLQ c30) between a self-management program (based on exercise and education) compared with usual care (control) after 3 months post-surgery or death (whichever occurs first) in patients with operable lung cancer</td></tr>
<tr><td colspan="2">Intervention: 12-week program comprising aerobic and resistance exercises, behavioural change counselling and resources (activity tracker, diary and booklet)</td></tr>
<tr><th>ESTIMAND</th><th>ANALYSIS</th></tr>
<tr><td>Target population</td><td>Analysis set</td></tr>
<tr><td>Patients with operable lung cancer</td><td>Full analysis population (§0)</td></tr>
<tr><td>Variable</td><td>Outcome measure</td></tr>
<tr><td>Physical function score as measured with the EORTC-QLQ c30</td><td>Physical function score measured 3 months post-surgery.</td></tr>
<tr><td>Handling of intercurrent events</td><td>Handling of missing data</td></tr>
<tr><td>• Deaths: while-alive strategy</td><td>Participants will be excluded if physical function not collected at 3 months.</td></tr>
<tr><td>Population-level summary measure</td><td>Analysis approach</td></tr>
<tr><td>Absolute difference in mean change in physical function score from baseline between groups at 3 months post-surgery.</td><td>Constrained longitudinal data analysis including all timepoints (baseline, 3, 6 and 12 months after surgery), with study group, time point and a study group by time point interaction and recruitment site included in the model. A contrast will be used to obtain the mean difference at 3 months and corresponding 95% confidence interval and p-value (§0).</td></tr>
</table>

Table 7: Estimand-to-analysis for the secondary endpoints

Secondary Objective	Treatments	Population	Variable & Outcome measure/s	Handling of intercurrent events*	Population-level summary	Analysis**
To assess the effect of a self-management program (exercise and education) on **physical function** in patients with operable lung cancer compared with usual care (control).	Usual care plus self-management program (exercise and education); usual care	Full analysis population (§0)	i. EORTC QLQ-c30 Physical function domain score at 6 and 12 months post- surgery<br>ii. SPPB overall and individual (gait, balance, and chair) scores at 3- and 6-months post-surgery	While-alive strategy	Absolute difference in mean change in physical function score from baseline between groups at 3 (for SPPB scores), 6- and 12-months post-surgery.	As per Table 6 with additional contrasts for comparisons at 6- and 12-months post-surgery.
To assess the effect of a self-management program (exercise and education) on **physical activity (levels and self-efficacy)** in patients with operable lung cancer compared with usual care (control).	Usual care plus self-management program (exercise and education); usual care	Full analysis population (§0)	IPAQ-SF total MET (minutes/week) and categorical (high/moderate/low); and barriers, task and walking self-efficacy scales at 3 and 6 months post-surgery. Sedentary activity based on average television and video viewing hours per day.	While-alive strategy	Absolute difference in mean total MET from baseline between groups at 3- and 6-months post-surgery.	As per Table 6 with additional contrasts for comparisons at 6 months post-surgery.
To assess the effect of a self-management program (exercise and education) on **muscle strength and function** in patients with operable lung cancer compared with usual care (control).	Usual care plus self-management program (exercise and education); usual care	Full analysis population (§0)	Quadriceps and hand grip strength test measured as a peak force and time to peak force at 3 and 6 months post-surgery	While-alive strategy	Absolute difference in mean change in muscle strength and function from baseline between groups at 3- and 6-months post-surgery.	As per Table 6 with additional contrasts for comparisons at 6 months post-surgery.
To assess the effect of a self-management program (exercise and education) on **health-related quality of life (HRQoL) and symptoms** in patients with operable lung cancer compared with usual care (control).	Usual care plus self-management program (exercise and education); usual care	Full analysis population (§0)	EORTC QLQ-c30 and LC13 domain and single item scores at 3-, 6- and 12-months post- surgery	While-alive strategy	Absolute difference in mean change in HRQoL score from baseline between groups at 3-, 6- and 12-months post-surgery.	As per Table 6 with additional contrasts for comparisons at 6- and 12-months post-surgery.
To assess the effect of a self-management program (exercise and education) on **fatigue** in patients with operable lung cancer compared with usual care (control).	Usual care plus self-management program (exercise and education); usual care	Full analysis population (§0)	Brief fatigue inventory global score at 3- and 6-months post-surgery	While-alive strategy	Absolute difference in mean change in fatigue score from baseline between groups at 3- and 6-months post-surgery.	As per Table 6 with additional contrasts for comparisons at 6 months post-surgery.
To assess the effect of a self-management program (exercise and education) on **sleep** in patients with operable lung cancer compared with usual care (control).	Usual care plus self-management program (exercise and education); usual care	Full analysis population (§0)	Sleep disturbance-short form 8b PROMIS Item Bank sleep T-score at 3- and 6-months post-surgery	While-alive strategy	Absolute difference in mean change in sleep T-score from baseline between groups at 3- and 6-months post-surgery.	As per Table 6 with additional contrasts for comparisons at 6 months post-surgery.
To assess the effect of a self-management program (exercise and education) on **distress** in patients with operable lung cancer compared with usual care (control).	Usual care plus self-management program (exercise and education); usual care	Full analysis population (§0)	Distress thermometer at 3- and 6-months post-surgery	While-alive strategy	Absolute difference in mean change in distress score from baseline between groups at 3- and 6-months post-surgery.	As per Table 6 with additional contrasts for comparisons at 6 post-surgery.
To assess the effect of a self-management program (exercise and education) on **financial toxicity** in patients with operable lung cancer compared with usual care (control).	Usual care plus self-management program (exercise and education); usual care	Full analysis population (§0)	COmprehensive score for financial toxicity (COST) at 3- and 6-months post-surgery	While-alive strategy	Absolute difference in mean change in financial toxicity score from baseline between groups at 3- and 6-months post-surgery.	As per Table 6 with additional contrasts for comparisons at 6 months post-surgery.
To assess the effect of a self-management program (exercise and education) on **survival** in patients with operable lung cancer compared with usual care (control).	Usual care plus self-management program (exercise and education); usual care	Full analysis population (§0)	Survival status at 12 months post-surgery	While-alive strategy	Hazard ratio with usual care as the reference.	Kaplan-Meier method to estimate survival probability at 12 months in each arm

						with corresponding 95% CI. Hazard ratio and 95% CI with control as reference.
To assess the effect of a self-management program (exercise and education) on **return to work/usual activities** in patients with operable lung cancer compared with usual care (control).	Usual care plus self-management program (exercise and education); usual care	Full analysis population (§0)	Number of weeks to usual activities for those not employed and number of weeks to return to work in those employed	While-alive strategy	Difference in mean time (in weeks) to return to work (or usual activities) between groups	An accelerated failure time survival model to estimate the difference between arms.

Intercurrent event for all objectives is death

6.4 Exploratory Efficacy Analyses

6.4.1 Complier average causal effect

A sensitivity analysis will estimate the treatment effect on the primary outcome assuming adherence to the intervention, as defined in Section 0. The complier average causal effect will be estimated using an instrumental variables approach where randomisation is the instrument for adherence. Two-stage least square models will be fit, with complier average causal effects (CACE) reported with 95% confidence intervals and *p*-values.

If there is missing data in the primary outcome at 3 months consideration will be made to use multiple imputation for the purpose of performing the CACE analysis as an additional analysis.

6.4.2 Subgroup analyses

An exploratory subgroup analysis based on postoperative cancer treatment will be performed for the primary outcome at 3-months post-surgery using the full analysis population. The subgroups are:

i. Postoperative cancer treatment: postoperative treatment/postoperative chemotherapy, radiotherapy or chemoradiotherapy
ii. No postoperative cancer treatment: postoperative treatment/postoperative chemotherapy, radiotherapy or chemoradiotherapy

Heterogeneity of the intervention effect according to postoperative cancer treatment (no postoperative treatment/postoperative chemotherapy, radiotherapy or chemoradiotherapy) will be assessed by including interactions between postoperative treatment and study group. The model will include as covariates the stratification factors used in randomisation, and an interaction term estimating the interaction between the intervention and the subgroup variable. Specific subgroup intervention effect estimates and corresponding 95% confidence intervals will be presented obtained from the model along with the interaction *p*-value. If there is no evidence of interaction (*p*-value >0.05), any differences between subgroups will be regarded as due to chance.

7 SAFETY ANALYSES

Safety outcomes will be reported for the intervention group only. For all minor and serious adverse events, data including pseudo study ID, date/time of adverse event, description of the adverse event and action taken will be reported for each participant experiencing an adverse event as a line listing.

7.1 Adverse Events

The following adverse events will be reported:

- fall not resulting in injury
- severe breathlessness
- new or progressive pain
- neurological deficits
- altered mental status
- palpitations
- light headedness
- progressive fatigue
- progressive anorexia

Adverse events reported as "Other" will be reviewed and assessed by the PI if they should also be reported. The number and percentage of participants with at least one adverse event will be reported along with the total number of episodes for adverse events. This will also be provided for each type of adverse event (listed above) separately.

7.2 Serious Adverse Events

The number and percentage of participants with at least one serious adverse event will be reported along with the total number of serious adverse events. This will also be provided for each type of serious adverse event (life-threatening or result in death or hospitalisation (or prolongation of hospitalisation), incapacity or disability) separately.

8 Figures

Where appropriate, graphical displays of the primary and secondary analyses will be given and convey information display in a corresponding summary table. A list and template for the planned figures is provided in Appendix A and Appendix B.

9 Reporting Conventions

- The mean, standard deviation, and any other statistics other than quantiles, will be reported to one decimal place greater than the original data.
- Quantiles, such as median, or minimum and maximum will use the same number of decimal places as the original data.
- Percentages will be reported with no decimal place for sample sizes less than 100 and with 1 decimal point if greater than 100.
- Estimated parameters, not on the same scale as raw observations (e.g. regression coefficients) will be reported to 3 significant figures.
- *p*-values >0.0001 will be reported to 4 decimal places; *p*-values less than 0.0001 will be reported as "<0.0001". This follows the guidelines from the target journal *Thorax*.
- In any cells in a row of data for which no data are reported, two mid-dots (··) will be included in the table and NA (Not Applicable) as a footnote.

For publication purposes, if the target journals guidelines differ in their reporting conventions to those listed above, the reporting will change to reflect the guidelines of the journal.

10 Technical Details

Analysis will be conducted using Stata/SE for Windows version 17.0 (64-bit, x64). We will report the software and version used at the time of reporting.

11 Summary of Changes to the Protocol

Due to various constraints, outcomes noted in sections 0 and 0 that were specified in the trial protocol will not be included in this analysis.

Appendix A – Table and Figure Listing for the Final Report

Heading	Title	Population Set
Figure 1	The CONSORT flow chart	Not applicable
Figure 2	Measures over time	Randomised
Figure 3	Kaplan-Meier survival curve	Randomised
Table 1.1	Summary of Subject Disposition during study period	Randomised
Table 1.2	Summary of Baseline Characteristics by treatment arm and overall	Randomised
Table 1.3	Summary of treatment compliance	Randomised
Table 2	Summary of primary outcome by treatment arm	Randomised
Table 3.1	Summary of secondary outcomes with measures at baseline	Randomised
Table 3.2	Summary of secondary outcome – IPAQ-SF categorical outcome activity	Randomised
Table 3.3	Summary of survival secondary outcome	Randomised
Table 3.4	Summary of secondary outcome return to work/usual activities	Randomised
Table 4	Summary of Adverse Events (Intervention arm only)	Safety
Table 5.1	Summary of Serious Adverse Events (Intervention arm only)	Safety
Table 5.2	Listing of Serious Adverse Events (Intervention arm only)	Safety

All figures and tables will be repeated for the Per Protocol analysis population described in Section 0.

APPENDIX B – TEMPLATES FOR TABLES AND FIGURES

Figure 1 – CONSORT Flow Chart

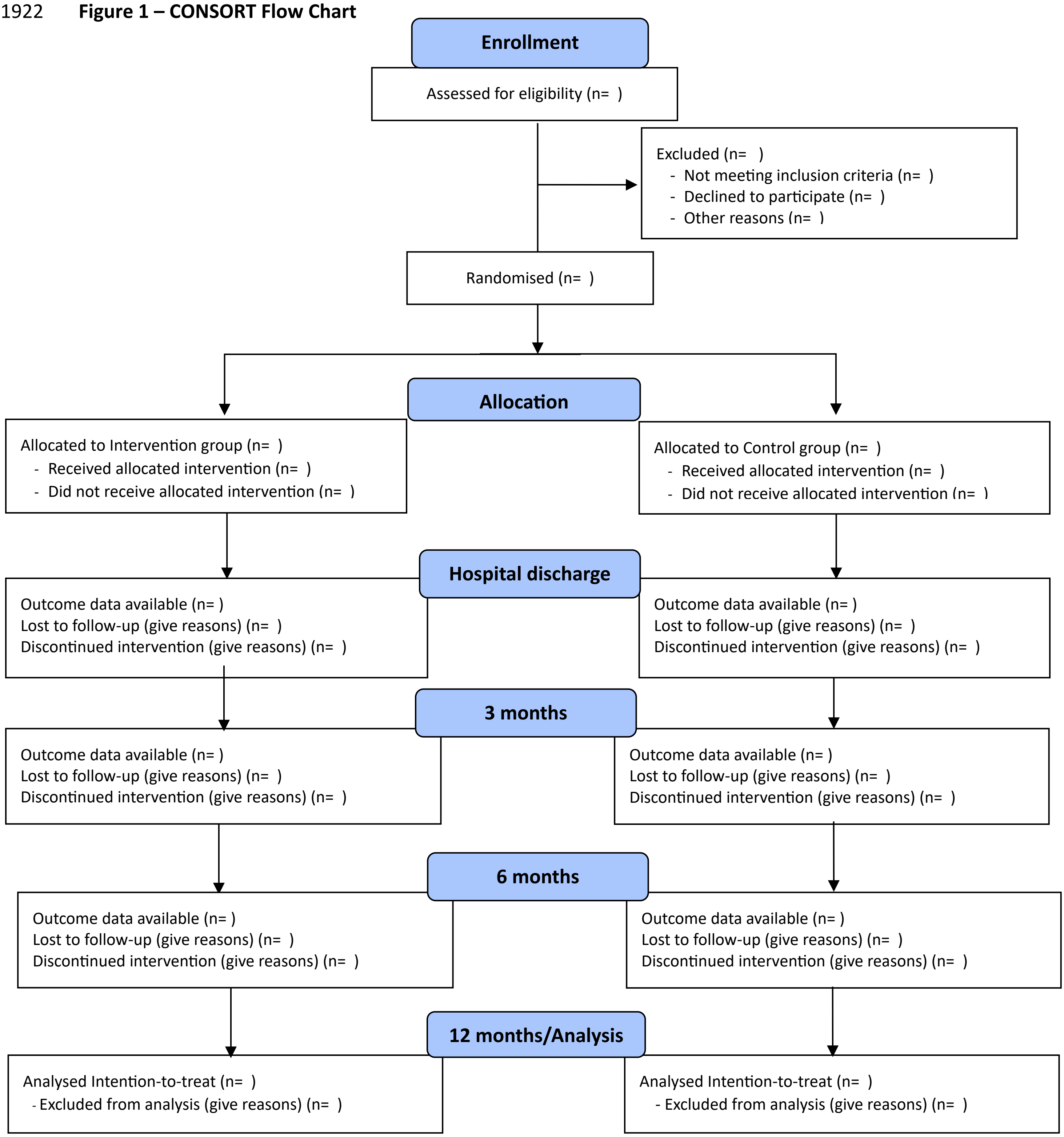

Figure 2 Measures over time (presented as mean and 95% confidence interval)

Mock example of QLQ-c30 Global score

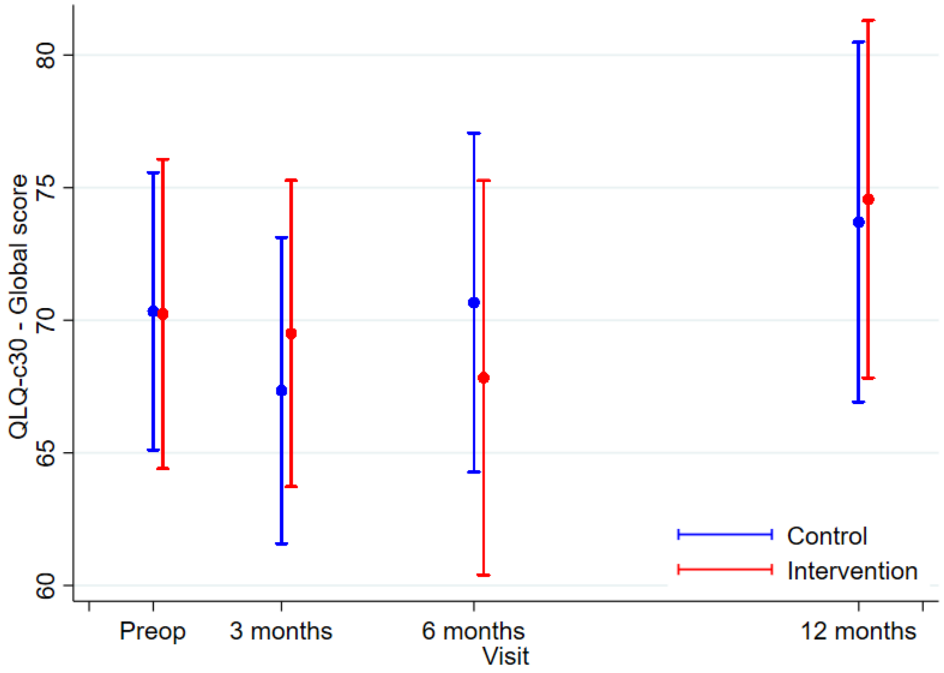

Figure 3 Kaplan-Meier survival curve

Mock example of survival curve

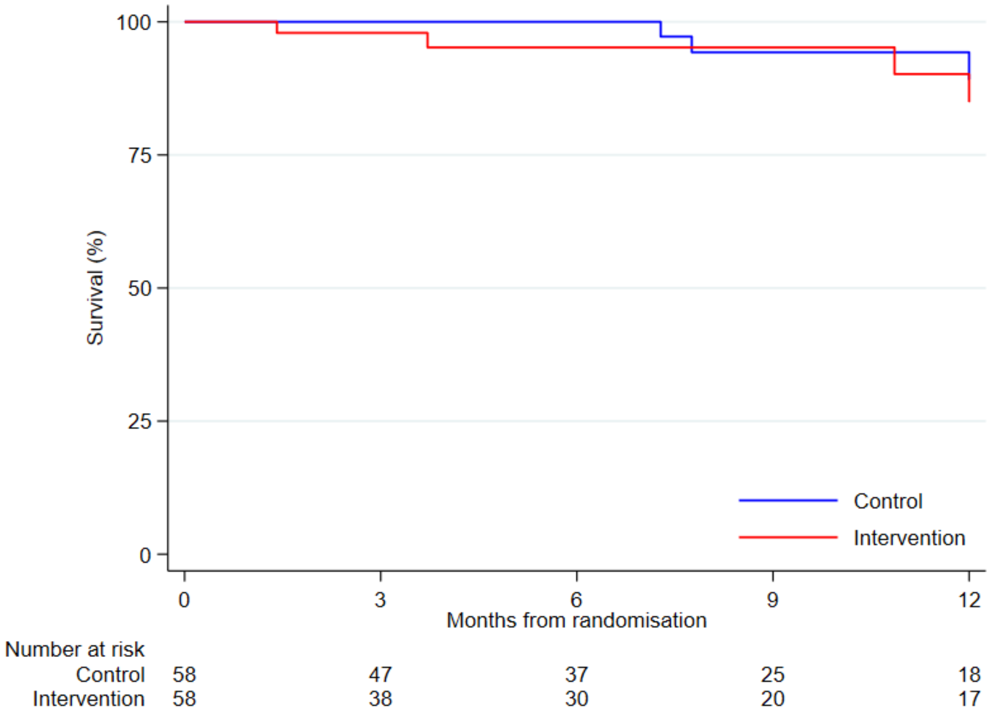

Table 1.1 - Summary of Subject Disposition during study period

Subject disposition	Control group N=	Intervention N=	All subjects N=
Randomised			
Discharged from hospital			
3 months post-surgery			
6 months post-surgery			
12 months post-surgery			
Early discontinuation			
Reason for discontinuation *			
Death			
Toxicity			
Treatment failure			
Withdrew consent			
Other			

Data are presented as n (%).
*More than one reason possible for discontinuation/withdrawal

Table 1.2 - Summary of Baseline Characteristics

Note: There may be some categorical variables such as occupation status and education level that will be reported based on collapsed similar categories or low frequencies. This will be decided at the discretion of the principle investigator.

Characteristic	Statistic	Control group N=	Intervention N=	All subjects N=
Age at surgery, years	Mean (SD)			
	Median [IQR]			
	Min- Max			
Sex				
Male	n (%)			
Female	n (%)			
CAPACITY study site				
Royal Melbourne Hospital	n (%)			
St Vincent’s Hospital	n (%)			
ECOG performance status				
0	n (%)			
1	n (%)			
2	n (%)			
Cancer histological type				
Squamous	n (%)			
Adenocarcinoma	n (%)			
Large cell	n (%)			
Other	n (%)			
- Other cancer type	n (%)			
- Non cancer	n (%)			
Cancer stage				
Stage IA	n (%)			

Characteristic	Statistic	Control group N=	Intervention N=	All subjects N=
Stage IB	n (%)			
Stage IIA	n (%)			
Stage IIB	n (%)			
Stage IIIA	n (%)			
Stage IV	n (%)			
Any co-morbidities	n (%)			
- COPD	n (%)			
- Tobacco consumption	n (%)			
- Diabetes	n (%)			
- Renal insufficiency	n (%)			
- Cardiovascular	n (%)			
- Neoplastic	n (%)			
- Alcoholism	n (%)			
Colinet co-morbidity Score	Mean (SD)			
	Median [IQR]			
	Min-Max			
0 (No comorbidities)	n (%)			
1	n (%)			
2	n (%)			
3	n (%)			
4	n (%)			
5	n (%)			
6	n (%)			
7 (all co-morbidities above)	n (%)			
Race/ethnicity				
White	n (%)			
Black/African American	n (%)			
Asian	n (%)			
American Indian or Alaska Native	n (%)			
Native Hawaiian/Pacific Islander	n (%)			
More than one race	n (%)			
Unknown	n (%)			
Body mass index	Mean (SD)			
	Median [IQR]			
	Min-Max			
Smoking history				
Never smoked	n (%)			
Quit smoking longer than 8 weeks ago	n (%)			
Current smoker	n (%)			
Education				
No formal schooling	n (%)			
Finished primary schooling	n (%)			

Characteristic	Statistic	Control group N=	Intervention N=	All subjects N=
Some or completed secondary or high school	n (%)			
Some or completed trade, community or TAFE college	n (%)			
Some university	n (%)			
Completed Bachelor's degree	n (%)			
Completed Masters or PhD degree	n (%)			
Other	n (%)			
Occupational status				
Working - Full Time (at least 35 hours per week)	n (%)			
Working - Part Time (less than 35 hours per week)	n (%)			
On paid or unpaid sick leave, but still employed	n (%)			
Temporarily laid off (without pay)	n (%)			
Unemployed	n (%)			
Going to school	n (%)			
Home maker/home duties/child care/elder care/ volunteer	n (%)			
Retired with/without any paid work	n (%)			
Receiving disability payments	n (%)			
Awaiting approval for disability payments	n (%)			
Other	n (%)			
Don’t know	n (%)			
No answer	n (%)			
Living arrangement				
Home alone	n (%)			
Home with family	n (%)			
Home with supports	n (%)			
Retirement village	n (%)			
Nursing home – low level care	n (%)			
Nursing home – high level care	n (%)			
Other	n (%)			
Respiratory function				
- FVC in litres actual	Mean (SD)			
	Median [IQR]			
	Min-Max			
- FVC in percent predicted	Mean (SD)			
	Median [IQR]			
	Min-Max			
- FEV1 in litres actual	Mean (SD)			
	Median [IQR]			
	Min-Max			

Characteristic	Statistic	Control group N=	Intervention N=	All subjects N=
- FEV1 in percent predicted	Mean (SD)			
	Median [IQR]			
	Min-Max			
- FEV1/FVC ratio	Mean (SD)			
	Median [IQR]			
	Min-Max			
- DLCO in ml/mmHg/min actual	Mean (SD)			
	Median [IQR]			
	Min-Max			
- DLCO in percent predicted	Mean (SD)			
	Median [IQR]			
	Min-Max			
**Surgical and hospital details**				
Type of surgery				
Lobectomy	n (%)			
Segmentectomy	n (%)			
Wedge resection	n (%)			
Pneumonectomy	n (%)			
Other (including combination)	n (%)			
Surgery side				
Left	n (%)			
Right	n (%)			
Surgery approach				
Thoracotomy	n (%)			
VATS or RATS	n (%)			
Other	n (%)			
Hospital length of stay, days	Mean (SD)			
	Median [IQR]			
	Min-Max			
Any postoperative complications				
ICU admission	n (%)			
Main reason for ICU admission				
Respiratory	n (%)			
Cardiovascular	n (%)			
Neurological	n (%)			
Renal	n (%)			
Other	n (%)			
ICU length of stay (days)	Mean (SD)			
	Median [IQR]			
	Min-Max			
ICU intubation	n (%)			
Mobility status				

**Characteristic**	**Statistic**	**Control group N=**	**Intervention N=**	**All subjects N=**
- Use of gait aid	n (%)			
SPS	n (%)			
2WF/4WF	n (%)			
GWF	n (%)			
Crutches	n (%)			
Other	n (%)			
- Level of independence				
Independent	n (%)			
1xA	n (%)			
2xA	n (%)			
Step t/f (1-2xA)	n (%)			
Hoist t/f only	n (%)			
Discharge destination				
Home alone	n (%)			
Home with family	n (%)			
Home with friends	n (%)			
GEM	n (%)			
Inpatient rehab	n (%)			
TCP (awaiting nursing home placement or respite)	n (%)			
Respite	n (%)			
Nursing home	n (%)			
Other	n (%)			
**Post-operative therapy**				
Type of chemotherapy or radiotherapy				
No chemo or RT	n (%)			
Post-op radical chemoRT	n (%)			
Post-op stereotactic radio (radical)	n (%)			
Post-op palliative radiotherapy	n (%)			
Post-op palliative chemotherapy	n (%)			
Post-op chemotherapy	n (%)			
Other	n (%)			

Note: If data are missing for a variable then an additional row will be included reporting the number of missing observation for that variable

Table 1.3 – Summary of treatment compliance (Intervention arm only)

Treatment compliance	Percentage
Adherence rate	
Completion rate	

Note: Adherence rate will be reported as the percentage of consultations delivered over the 12 weeks (number delivered against target of 12 sessions). Completion rate will be reported as the percentage of participants who continue consultations to 12 weeks.

Table 2 - Summary of primary outcome by treatment arm (Full analysis population)

Primary Outcome	Control group<br>N=<br>Mean ± SD	Intervention<br>N=<br>Mean ± SD	Estimate (95% CI)<br>(Absolute difference: Invention minus control)	p-value
EORTC QLQ-c30 Physical function score				
Complier average causal effect (Sensitivity analysis)				
Complier average causal effect (Sensitivity analysis, multiple imputation)				
Subgroup analysis (§0/§0)				
Postoperative cancer treatment				
No postoperative cancer treatment				

Note: Mean change from baseline at 3 months post-surgery

Table 3.1 - Summary of continuous secondary outcomes with measures at baseline

Secondary Outcome*	Control group<br>N=<br>Mean ± SD	Intervention<br>N=<br>Mean ± SD	Estimate (95% CI)<br>(Absolute difference: Invention minus control)
**Physical Function**			
EORTC QLQ-c30 Physical function score			
At 6-months post-surgery			
At 12-months post-surgery			
SPPB overall score			
At 3-months post-surgery			
At 6-months post-surgery			
SPPB gait score			
At 3-months post-surgery			
At 6-months post-surgery			
SPPB balance score			
At 3-months post-surgery			
At 6-months post-surgery			
SPPB chair			
At 3 months post-surgery			
At 6-months post-surgery			
**Physical activity (levels and self-efficacy)**			
IPAQ-SF total MET (minutes/week)			
At 3 months post-surgery			
At 6-months post-surgery			
Self-efficacy scales			

Secondary Outcome*	Control group N= Mean ± SD	Intervention N= Mean ± SD	Estimate (95% CI) (Absolute difference: Invention minus control)
Barriers			
At 3 months post-surgery			
At 6-months post-surgery			
Tasks			
At 3 months post-surgery			
At 6-months post-surgery			
Walking			
At 3 months post-surgery			
At 6-months post-surgery			
**Muscle Strength and Function**			
Quadriceps strength (peak force)			
At 3 months post-surgery			
At 6-months post-surgery			
Quadriceps strength (time to peak force)			
At 3 months post-surgery			
At 6-months post-surgery			
Hand group strength (peak force)			
At 3 months post-surgery			
At 6-months post-surgery			
Hand group strength (time peak force)			
At 3 months post-surgery			
At 6-months post-surgery			
**Health-Related Quality of Life (HRQoL) and symptoms**			
EORTC QLQ-c30 scales			
Global health status/QoL			
At 3 months post-surgery			
At 6-months post-surgery			
At 12-months post-surgery			
Role functioning			
At 3 months post-surgery			
At 6-months post-surgery			
At 12-months post-surgery			
Emotional functioning			
At 3 months post-surgery			
At 6-months post-surgery			
At 12-months post-surgery			
Cognitive functioning			
At 3 months post-surgery			
At 6-months post-surgery			
At 12-months post-surgery			
Social functioning			
At 3 months post-surgery			
At 6-months post-surgery			
At 12-months post-surgery			
Fatigue			
At 3 months post-surgery			

Secondary Outcome*	Control group N= Mean ± SD	Intervention N= Mean ± SD	Estimate (95% CI) (Absolute difference: Invention minus control)
At 6-months post-surgery			
At 12-months post-surgery			
Nausea and vomiting			
At 3 months post-surgery			
At 6-months post-surgery			
At 12-months post-surgery			
Pain			
At 3 months post-surgery			
At 6-months post-surgery			
At 12-months post-surgery			
Dyspnoea			
At 3 months post-surgery			
At 6-months post-surgery			
At 12-months post-surgery			
Insomnia			
At 3 months post-surgery			
At 6-months post-surgery			
At 12-months post-surgery			
Appetite loss			
At 3 months post-surgery			
At 6-months post-surgery			
At 12-months post-surgery			
Constipation			
At 3 months post-surgery			
At 6-months post-surgery			
At 12-months post-surgery			
Diarrhoea			
At 3 months post-surgery			
At 6-months post-surgery			
At 12-months post-surgery			
Financial difficulties			
At 3 months post-surgery			
At 6-months post-surgery			
At 12-months post-surgery			
EORTC QLQ-LC13 scales			
Dyspnoea			
At 3 months post-surgery			
At 6-months post-surgery			
At 12-months post-surgery			
Coughing			
At 3 months post-surgery			
At 6-months post-surgery			
At 12-months post-surgery			
Haemoptysis			
At 3 months post-surgery			
At 6-months post-surgery			

Secondary Outcome*	Control group<br>N=<br>Mean ± SD	Intervention<br>N=<br>Mean ± SD	Estimate (95% CI)<br>(Absolute difference:<br>Invention minus control)
At 12-months post-surgery			
Sore mouth			
At 3 months post-surgery			
At 6-months post-surgery			
At 12-months post-surgery			
Dysphagia			
At 3 months post-surgery			
At 6-months post-surgery			
At 12-months post-surgery			
Peripheral neuropathy			
At 3 months post-surgery			
At 6-months post-surgery			
At 12-months post-surgery			
Alopecia			
At 3 months post-surgery			
At 6-months post-surgery			
At 12-months post-surgery			
Pain in chest			
At 3 months post-surgery			
At 6-months post-surgery			
At 12-months post-surgery			
Pain in arm or shoulder			
At 3 months post-surgery			
At 6-months post-surgery			
At 12-months post-surgery			
Pain in other parts			
At 3 months post-surgery			
At 6-months post-surgery			
At 12-months post-surgery			
**Fatigue**			
Brief fatigue inventory global score			
At 3 months post-surgery			
At 6-months post-surgery			
**Sleep**			
Sleep disturbance-short form 8b PROMIS Item Bank sleep T-score			
At 3 months post-surgery			
At 6-months post-surgery			
**Distress**			
Distress thermometer			
At 3 months post-surgery			
At 6-months post-surgery			
**Financial Toxicity**			
At 3 months post-surgery			
At 6-months post-surgery			

Table 3.2a - Summary of secondary outcome – IPAQ-SF categorical outcome for activity level

Secondary Outcome	Control group n (%)			Intervention n (%)		
IPAQ-SF – categorical	**Baseline**	**3 months**	**6 months**	**Baseline**	**3 months**	**6 months**
Low						
Moderate						
High						
Missing (count only)						

And/Or

Table 3.2b - Summary of secondary outcome – IPAQ-SF categorical outcome for activity level

	Moderate vs Low RRR (95% CI)	High vs Low RRR (95% CI)
At 3 months post-surgery		
At 6-months post-surgery		

Note: Intervention vs Control (reference)

Table 3.3 - Summary of survival secondary outcome

Secondary Outcome	Control group N=	Intervention N=	HR (95% CI)
Survival probability* at 12 months post-surgery			

*Note: Reported with 95% CI.

Table 3.4 - Summary of secondary outcome return to work/usual activities

Secondary Outcome	**Control group** Mean±SD	**Intervention** Mean±SD	Estimate (95% CI) (Absolute difference: Invention minus control)
Time to return to work (including non-paid volunteer work)	N=	N=	
Time to return to usual activities	N=	N=	

Note: The sample size for each outcome will not overlap as patients will be classified as either working prior to surgery or not working prior to surgery. If the distribution of the outcome is skewed then the median and IQR, median difference and corresponding 95% CI will be reported.

Table 4 - Summary of adverse events (Intervention arm only)

Adverse Event	n (%)	Number of episodes
Fall not resulting in injury		
Severe breathlessness		
New or progressive pain		
Neurological deficits		

Adverse Event	n (%)	Number of episodes
Altered mental status		
Palpitations		
Light headedness		
Progressive fatigue		
Progressive anorexia		

Note: The number and percentage reflect participants in the intervention arm who experienced at least one episode of that adverse event type.

Table 5.1 Summary of serious adverse events (Intervention arm only)

Serious Adverse Event	n (%)	Number of episodes
Death		
Life threatening		
Required hospitalisation or prolonged existing hospitalisation		
Resulted in disability or incapacity		

Note: The number and percentage reflect participants in the intervention arm who experienced at least one episode of that serious adverse event type.

Table 5.2 Listing of serious adverse events (Intervention arm only)

Subject number	Date of SAE	Serious adverse event type	Description of SAE	Action taken

APPENDIX C – DATA MAPPING TO DATABASE

Table 8 Data mapping of baseline characteristics

	Characteristic	Data type	Variable name in database	Derived (outside of database)
**Baseline demographics**				
	Age at surgery	Continuous	age	No
	Sex	Binary	sex	No
	CAPACITY study site	Categorical	site	No
	ECOG performance status	Categorical	ecog_dr	No
	Cancer histological type	Categorical	histological_type	No
	Cancer stage	Categorical	tnm_stage	No
	Comorbidities<br>- COPD<br>- Tobacco consumption<br>- diabetes<br>- renal insufficiency<br>- respiratory<br>- cardiovascular<br>- neoplastic<br>- alcoholism<br>- Colinet comorbidity Score	Binary<br><br><br><br><br><br><br><br>Discrete	copd<br>tobacco_consumption<br>diabetes_mellitus_treated<br>renal_insufficiency<br>respiratory<br>cardiovascular<br>neoplastic<br>alcoholism<br>colinet_comorbidity_score	No
	Race/ethnicity	Categorical	race	No
	Body mass index	Continuous	bmi	No
	Smoking history	Categorical	smoking_status	No
	Education	Categorical	highest_education	No
	Occupational status	Categorical	employment_2_grouped	Yes, collapsed categories as per Table 1.2
	Living arrangement	Categorical	social_situation	No
	Respiratory function	Continuous		No

	Characteristic	Data type	Variable name in database	Derived (outside of database)
	- FVC in litres actual<br>- FVC in percent predicted<br>- FEV1 in litres actual<br>- FEV1 in percent predicted<br>- FEV1/FVC in percent predicted<br>- DLCO in ml/mmHg/min actual<br>- DLCO in percent predicted		fvc_litres<br>fvc_litres_percentpred<br>fev1_litres<br>fev1_litres_percentpred<br>fev1_over_fvc_percentpred<br>dlco_actual<br>dlco_percentpred	
**Surgical and hospital details**				
	Type of surgery<br>Side of surgery<br>Surgery approach	Categorical	type_of_surgery<br>type_of_surgery_side<br>type_of_surgery_approach	No
	Hospital length of stay	Continuous	los_surgtodc	No
	Any postoperative complications	Binary	hosp_comp	No
	ICU admission<br>Main reason for ICU admission<br>ICU length of stay<br>ICU intubation	Binary<br>Categorical<br>Continuous<br>Binary	hosp_icu<br>hosp_icu_reason<br>hosp_icu_nodays<br>hosp_icu_vent	No
	Mobility status<br>- Use of gait aid<br>- Level of independence	Binary<br>Categorical	dc_mob_aid, dc_mob_aid_yess<br>dc_mob_assistance	No
	Discharge destination	Categorical	dc_dest	No
**Prior therapy**				
	Type of chemotherapy or radiotherapy	Categorical	rx_type	No

Table 9 Data mapping of primary and secondary outcomes

	Outcome	Data type	Variable name in database	Derived (outside of database)	Value range
Primary outcome					
	EORTC QLQ C30 – physical function	Continuous	PF2	Yes	[0, 100]
Secondary outcomes					
	Short Physical Performance Battery SPPB				
	Total Balance test score (0-4)	Continuous	sppb_balance_score	No	[0, 4]
	Gait speed Test score (0-4)	Continuous	sppb_gait_score	No	[0, 4]
	Chair Stand Test score (0-4)	Continuous	sppb_chair_score	No	[0, 4]
	Total SPPB score (0-12)	Continuous	sppb_sum_ordinal_score	No	[0, 12]
	EORTC QLQ C30				
	Global	Continuous	QL2	Yes	[0, 100]
	Physical functioning	Continuous	PF2	Yes	[0, 100]
	Role functioning	Continuous	RF2	Yes	[0, 100]
	Emotional functioning	Continuous	EF	Yes	[0, 100]
	Cognitive functioning	Continuous	CF	Yes	[0, 100]
	Social functioning	Continuous	SF	Yes	[0, 100]
	Fatigue	Continuous	FA	Yes	[0, 100]
	Nausea and Vomiting	Continuous	NV	Yes	[0, 100]
	Pain	Continuous	PA	Yes	[0, 100]
	Dyspnea	Continuous	DY	Yes	[0, 100]
	Insomnia	Continuous	SL	Yes	[0, 100]
	Appetite loss	Continuous	AP	Yes	[0, 100]
	Constipation	Continuous	CO	Yes	[0, 100]
	Diarrhea	Continuous	DI	Yes	[0, 100]
	Financial difficulties	Continuous	FI	Yes	[0, 100]
	EORTC QLQ-LC13				

	Outcome	Data type	Variable name in database	Derived (outside of database)	Value range
	Dyspnoea	Continuous	LCDY	Yes	[0, 100]
	Coughing	Continuous	LCCO	Yes	[0, 100]
	Haemoptysis	Continuous	LCHA	Yes	[0, 100]
	Sore mouth	Continuous	LCSM	Yes	[0, 100]
	Dysphagia	Continuous	LCDS	Yes	[0, 100]
	Peripheral neuropathy	Continuous	LCPN	Yes	[0, 100]
	Alopecia	Continuous	LCHR	Yes	[0, 100]
	Pain in chest	Continuous	LCPC	Yes	[0, 100]
	Pain in arm or shoulder	Continuous	LCPA	Yes	[0, 100]
	Pain in other parts	Continuous	LCPO	Yes	[0, 100]
	6-minute walk distance (6MWD)				
	6 MW distance	Continuous	sixmin_distance_max	Yes, best of 2 attempts, otherwise first attempt: [sixmin_distance_1], [sixmin_distance_2]	[0, 800 meters]
	Muscle strength				
	Quadriceps strength test (bilateral)<br>- Peak force over 6s<br>- Time to peak force over 6s	Continuous	strength_knee_left_highest<br>strength_knee_left_longesttime_2<br>strength_knee_right_highest<br>strength_knee_right_longesttime	No	[0, 136 kg]<br><br>[0, 6s]
	Hand grip strength test (bilateral)<br>- Highest strength over 6s	Continuous	strength_grip_right_average<br>strength_grip_left_average	No	[0, 91 kg (200 lbs)]
	IPAQ-SF				
	Total MET-minutes/week of PA	Continuous	pa_met	Yes	[153, 14688]
	Categorical Score of IPAQ	Categorical	ipaq_pa_group	Yes	Coded as:<br>0 – Low<br>1 – Moderate

	Outcome	Data type	Variable name in database	Derived (outside of database)	Value range
					2 – High
	Sedentary time	Continuous	acsm_2	No	[0,24 hours]
	Self-efficacy for PA				
	Walking score	Continuous	eff_walk_avg	Yes	[0, 100]
	Barrier score	Continuous	eff_barrier_avg	Yes	[0, 100]
	Task score	Continuous	eff_task_avg	Yes	[0, 100]
	Brief Fatigue Inventory	Continuous	bfi_global	Yes	[0, 10]
	Distress Thermometer	Continuous	q_distress_thermometer	No	[0, 10]
	Sleep Disturbance-short form 8b PROMIS	Continuous	T_score_sleep	Yes	[28.9, 76.5]
	COmprehensive Score for financial Toxicity (COST)	Continuous	finance_total	Yes	[0, 44]
	Return to usual activities	Continuous	employment_5_weeks	No	[0, 52 weeks]
	Return to work	Continuous	employment_8_weeks	No	[0, 52 weeks]
	Survival				
	Survival status	Binary	alive_at_12mnth	No	Coded as 1 – alive, 0 – deceased
			surv_status	Yes	Coded as 0 – alive, 1- deceased
	Follow-up time	Continuous	death_surgtodeath_days	No	[0, 365 days]
			surv_time	Yes	

Table 10 Data mapping of safety outcomes

	Outcome	Data type	Variable name in database	Derived (outside of database)
Safety outcome (Minor adverse event)				
	Adverse event<br>- fall not resulting in injury<br>- severe breathlessness<br>- new or progressive pain<br>- neurological deficits<br>- altered mental status<br>- palpitations<br>- light headedness<br>- progressive fatigue<br>- progressive anorexia	Binary	ae_minor_type<br>ae1<br>ae2<br>ae3<br>ae4<br>ae5<br>ae6<br>ae7<br>ae8<br>ae9	Yes, only ae1 to ae9 derived from ae_minor_type, for reporting purposes binary indictors will be created
Safety outcome (Serious adverse event)				
	Date of Adverse Event	Date	ae_dtm	No
	Serious adverse event type<br>- resulted in death<br>- life threatening<br>- required hospitalisation or prolonged existing hospitalisation<br>- resulted in disability or incapacity	Categorical	ae_serious_type	No
	Description of SAE	String	ae_description	No
	Action taken	String	ae_action	No
