# Supplementary material for: Home-Based Exercise and Self-Management After Lung Cancer Resection: A Randomized Clinical Trial
Source: JAMA Netw Open. 2024 Dec 2;7(12):e2447325. doi: 10.1001/jamanetworkopen.2024.47325 (PMC11612835; doi:10.1001/jamanetworkopen.2024.47325)
Supplement: Supplement 2. — eMethods. eTable 1. Physical Function (Primary Outcome) Measured With the EORTC QLQ-c30 at 3 Months Post-operatively, Including Subgroup Analysis eTable 2. Summary of Continuous Secondary Outcomes (Health-Related Quality of Life and Symptoms) With Measures at Baseline eTable 3. Summary of Secondary Outcome (IPAQ-SF Categorical Outcome for Activity Level) eFigure. Mean Objectively Measured Physical Function (Short Physical Performance Battery) Over Time eReferences. [file jamanetwopen-e2447325-s002.pdf]

Supplementary Online Content

Granger CL, Edbrooke L, Antippa P, et al. Home-based exercise and self-management after lung cancer resection: a randomized clinical trial. *JAMA Netw Open.* 2024;7(12):e2447325. doi:10.1001/jamanetworkopen.2024.47325

eMethods.

eTable 1. Physical Function (Primary Outcome) Measured With the EORTC QLQ-c30 at 3 Months Post-operatively, Including Subgroup Analysis

eTable 2. Summary of Continuous Secondary Outcomes (Health-Related Quality of Life and Symptoms) With Measures at Baseline

eTable 3. Summary of Secondary Outcome (IPAQ-SF Categorical Outcome for Activity Level)

eFigure. Mean Objectively Measured Physical Function (Short Physical Performance Battery) Over Time

eReferences.

This supplementary material has been provided by the authors to give readers additional information about their work.

© 2024 Granger CL et al. *JAMA Network Open.*

eMethods.

Inclusion Criteria – additional details

Patients were eligible for inclusion if they were adults (aged 18 years or over), scheduled to receive surgical treatment for suspected or confirmed NSCLC, able to provide written informed consent for the trial and were expected to be alive for greater than six months from surgery. At study entry they were required to have an Eastern Cooperative Oncology Group (ECOG) performance status of zero to two and not already meeting the recommended volume of aerobic physical activity (150 minutes or more of moderate intensity, or 75 minutes or more of vigorous intensity physical activity per week). Patients were excluded if they did not have sufficient English language skills to complete questionnaires in English, had cognitive impairment to a level they could not provide consent for surgery, had stage IV disease at study entry, or any of the following concurrent medical issues affecting their potential safety to exercise unsupervised in the home setting: acute uncontrolled cardiovascular or respiratory issues, decompensated heart failure, severe aortic stenosis, uncontrolled arrhythmia, or acute coronary syndrome. Patients were excluded if at study entry they were non-ambulant, or ECOG performance-status three or above.

Randomization and blinding – additional details

Primary investigators (CG, SP), trial coordinator (SA), and trial physiotherapists were unblinded to group assignment. Statisticians (DZ, KL) were blinded until the statistical analysis plan was finalised, database cleaned, and the blinded data review had occurred. All other investigators were blinded throughout the study. Trial assessors were blinded and any occurrences of their unblinding recorded (and subsequent assessments completed with different assessors). Hospital staff delivering usual care were blinded. Due to the nature of the intervention, it is possible that patients were unblinded, although attempts were made to avoid specific allocation disclosure and trial staff did not discuss treatment details with patients at assessment time-points. Patients were informed the trial was comparing two types of physiotherapist-led treatments because patients in both arms received usual care physiotherapy treatment as inpatients after surgery.

Outcome – additional details

The European Organization for the Research and Treatment of Cancer Quality of Life Questionnaire (EORTC QLQ-C30) and lung cancer subscale (LC13)[6,7] is a self-reported measure of HRQoL and symptoms. The primary

© 2024 Granger CL et al. *JAMA Network Open.*

endpoint for the trial was the physical function domain. The global health status (global HRQoL), other functional domains, symptom scales and single item symptom scores were analysed as secondary outcomes. Lower scores on the global health status and functional domains indicate worse health/function, whereas higher scores on the symptom scales/items indicate worse symptoms. In the physical function domain, the threshold for clinical importance is less than 83 out of 100 points.[8] The minimal clinical importance difference (MCID) range (small difference) for patients undergoing cancer treatment in the physical function domain is 4 to 15 points and for global health status (HRQoL) is 4 to 10 points.[6,7]

The Short Physical Performance Battery (SPPB)[9] is an objective measure of physical function. The test includes three sub-scales (gait, balance and chair rise test) each scored 0 to 4 points, and an overall score out of 12. Higher scores indicate better performance. The MCID range for patients with chronic obstructive pulmonary disease is 0.83 to 0.96 points.[10]

The 6-minute walk test is an objective measure of functional exercise capacity. The participant is asked to walk up and down a 30-meter track. Repeated tests are conducted to account for the learning effect. The test was completed according to the American Thoracic Society guidelines.[11] Greater distance indicates better performance. The MCID range for lung cancer is 22 to 42 metres.[12]

Quadriceps and hand-grip muscle strength were assessed by hand-held and hand-grip dynamometry.[13] Three repeat tests were performed after a practice, and the highest score utilised for analyses. Higher peak force and shorter time to reach peak force indicate better performance.

Participants completed several patient-reported outcome measures using questionnaires in addition to the EORTC QLQ C30 already described. This included:

- Physical activity levels measured using the International Physical Activity Questionnaire short-form (IPAQ).[14] This asks participants about their level of physical activity over the last seven days. The output is reported as a continuous variable of total metabolic equivalent (MET) minutes per week (higher MET minutes/week indicate higher levels of physical activity) and a categorical score (low, moderate or high physical activity levels).
- Exercise self-efficacy measured using the Barrier, task and walking exercise self-efficacy scales.[15,16] Each scale is scored from 0 to 100%, with a lower score indicating lower self-efficacy.

© 2024 Granger CL et al. *JAMA Network Open.*

- Fatigue measured using the Brief Fatigue Inventory.[17] This is scored from 0 to 10 with higher scores indicating worse fatigue/interference.
- Sleep quality measured using the Sleep Disturbance-Short Form 8b PROMIS Item Bank V.1.0.[18] Higher scores indicate more disturbed sleep.
- Distress measured using the Distress Thermometer.[19] This is scored from 0 to 10 with higher scores indicating higher distress.
- Financial toxicity measured using the COmprehensive Score for financial Toxicity (COST) Version 1.[20] This is scored from 0 to 44 with lower scores indicating worse financial wellbeing. Financial toxicity was assessed given that a potential benefit of the intervention could be to prevent need to present to hospital because of increased capacity for self-management.

Statistical analysis - additional details

Analyses were performed in Stata/ SE, version 17.0 (Stata Corporation, College Station, TX, USA).

Outcomes were summarised using frequencies and percentages (based on the non-missing sample size) for categorical/binary variables, mean and standard deviation for continuous variables, or median and quartiles (25th and 75th percentile) for non-symmetrical continuous variables.

The secondary categorical outcome, IPAQ physical activity with “High”, “Moderate”, “Low” responses were combined to form a binary response of “Low” versus “Moderate/High” and was analysed using a likelihood-based longitudinal data analysis model with a logistic link function. The model included factors representing study group allocation, time point and a study group allocation by time point interaction and recruitment site included in the model. Results were expressed as an Odds Ratio (with corresponding 95% confidence intervals) for the relative comparison of the odds of the outcome compared between study groups at 3 months and 6 months post-randomisation.

The Kaplan-Meier method was used to estimate the survival probability at 12 months post-operative in each group. Follow-up time was measured from the date of operation until the date of death from any cause or date last known alive. Participants who were alive by the study closeout date were censored. Participants who withdrew or were lost to follow-up before the closeout date were censored at the date they were last known

© 2024 Granger CL et al. *JAMA Network Open.*

to be alive. Due to the small number of deaths, an estimate of the hazard ratio (HR) and corresponding 95% CI for time to death between the arms with the control group as the reference was not calculated.

COVID-19 Pandemic Modifications

The following important modifications were made to the trial protocol in 2020 by the investigators to allow the trial to be completed within the local hospital and government restrictions. Modifications were reviewed where required and approved by the funding bodies and ethics committee (see Supplement 1.2 for list of ethics amendments). Supplement 2 contains the CONSERVE-CONSORT checklist with itemised important trial modifications.

Outcomes

The assessment visit windows for the 3- and 6-month in-person assessments were widened from +/- 14 days to +/- 28 days to allow increased possibility of completing these in-person assessments outside of any periods of isolation. Isolations occurred regularly over 2020 and 2021 for reasons including: periods of home lockdown ordered by the government (262 days in total over years 2020 and 2021) restricting patients from leaving their home for research purposes, local hospital restrictions for staff and patient contact limiting in-person assessments for research in the home or hospital settings, and the required 14 days of COVID-19 home isolation for anyone contracting the illness. Additionally patients who were unable to complete in-person testing were offered to complete the PRO aspect of the assessment remotely via questionnaires sent in the post or with the assessor over the telephone, or a combination of the two. On these occasions objective data (6-minute walk test, Short Physical Performance Battery and muscle strength tests) were omitted.

Recruitment and funding

The duration of the trial including funding period was extended due to a longer than predicted recruitment duration. At the start of the pandemic only one site had opened for recruitment (The Royal Melbourne Hospital). This site was required to pause recruitment from March 23, 2020 to February 12, 2021. Opening of recruitment at the second site (St Vincent’s Hospital Melbourne) was delayed until September 2021 and the third site (Austin Hospital) never opened for recruitment as the final sample size was reached prior to this being possible. Recruitment numbers from the sites are uneven and strongly biased towards site 1. The recruitment rate for the trial was also lower than predicted. Once recruitment for the trial re-opened in

© 2024 Granger CL et al. *JAMA Network Open.*

February 2021 the lung cancer services primarily converted from an in-person outpatient clinic to a telehealth clinic. The protocol was modified to allow electronic consent via Research Electronic Data Capture (REDCap) for those patients who were approached and consented remotely.

© 2024 Granger CL et al. *JAMA Network Open.*

eTable 1. Physical Function (Primary Outcome) Measured With the EORTC QLQ-c30 at 3 Months Post-operatively, Including Subgroup Analysis

**Primary outcome**	**Control (n = 58)** mean (SD)	**Intervention (n = 58)** mean (SD)	**Mean difference* (95% CI)**	**p-value**
Physical functioning scale**	76.3 (18.8)	77.3 (20.9)	1.0 [8.0 to -6.0]	0.78
Secondary analysis				
Complier average causal effect	n/a	n/a	0.7 [8.3 to -7.0]	0.86
Complier average causal effect (multiple imputation)	n/a	n/a	0.3 [9.5 to -8.8]	0.95
*Subgroup analysis****				0.01
- Postoperative cancer treatment	74.4 (16.4)	70.2 (24.9)	-8.8 [13.2 to -2.3]	
- No postoperative cancer treatment	77.9 (20.8)	80.9 (17.9)	5.4 [1.4 to -19.0]	

Abbreviations: EORTC QLQ-c30, European Organization for the Research and Treatment of Cancer Quality of Life Questionnaire. *Footnotes:*

* The model estimates the mean difference between intervention and control from a common baseline so that any change over time between the groups is not due to any difference in baseline scores. Differences use control as the reference group. Baseline scores were mean 83.2 units (SD 16.9) and 85.8 units (SD 15.9) for the control arm and intervention arm, respectively.

** Score 0 to 100 with lower scores indicating worse function.

*** P-value for the interaction at the primary time-point presented.

© 2024 Granger CL et al. *JAMA Network Open.*

eTable 2. Summary of Continuous Secondary Outcomes (Health-Related Quality of Life and Symptoms) With Measures at Baseline

**Variable**	**Control group** mean (SD)	**Intervention** mean (SD)	**Mean difference* (95% CI)**
**EORTC QLQ-c30 scales[1]**			
Global health status/QoL			
Baseline	71.3 (20.4)	69.9 (21.1)	-
3 months	65.4 (20.4)	72.0 (19.4)	7.1 [13.8-0.4]
6 months	69.5 (24.0)	69.2 (22.5)	0.5 [8.2 to -7.3]
12 months	70.2 (21.2)	70.6 (19.7)	2.8 [9.8 to -4.1]
Physical functioning			
Baseline	83.2 (16.9)	85.8 (15.9)	-
6 months	75.6 (21.6)	77.5 (19.6)	0.5 [7.6 to -6.5]
12 months	79.3 (19.6)	79.4 (21.1)	0.2 [7.4 to -7.0]
Role functioning			
Baseline	77.2 (31.1)	79.2 (27.1)	-
3 months	73.8 (27.6)	78.1 (26.5)	4.8 [14.5 to -5.0]
6 months	69.3 (30.2)	77.1 (25.7)	6.9 [17.2 to -3.5]
12 months	81.3 (24.0)	77.0 (29.8)	-3.9 [5.3 to -13.1]
Emotional functioning			
Baseline			-
3 months	74.8 (23.7)	74.8 (23.6)	0.7 [8.6 to -7.3]
6 months	78.3 (19.3)	73.5 (22.7)	-1.5 [5.4 to -8.5]
12 months	80.8 (19.0)	75.9 (20.8)	-1.5 [4.6 to -7.6]
Cognitive functioning			
Baseline	71.3 (23.5)	71.3 (27.1)	-
3 months	78.4 (20.1)	83.0 (20.3)	4.6 [12.3 to -3.0]
6 months	82.3 (18.9)	77.9 (18.1)	-2.7 [3.7 to -9.1]
12 months	80.3 (20.3)	81.1 (23.5)	2.6 [10.4 to -5.3]
Social functioning			
Baseline	78.4 (32.9)	77.5 (26.6)	-
3 months	70.1 (32.6)	71.1 (32.3)	2.8 [14.4 to -8.7]
6 months	74.7 (31.5)	77.9 (26.2)	5.5 [15.7 to -4.8]
12 months	84.4 (26.0)	78.5 (29.6)	-3.5 [6.1 to -13.1]
Fatigue			
Baseline	27.1 (24.6)	25.6 (21.0)	-
3 months	37.9 (25.4)	32.1 (24.0)	-6.0 [2.8 to -14.8]
6 months	32.4 (24.5)	33.6 (22.7)	1.5 [9.7 to -6.8]
12 months	28.6 (22.8)	29.9 (25.1)	-0.1 [8.6 to -8.9]
Nausea and vomiting			
Baseline	4.7 (10.8)	8.8 (17.3)	-
3 months	9.9 (16.4)	8.5 (20.0)	-1.8 [5.3 to -8.9]
6 months	5.0 (15.5)	9.3 (14.7)	3.2 [9.0 to -2.5]
12 months	7.1 (16.3)	5.6 (11.8)	-1.9 [3.9 to -7.7]

© 2024 Granger CL et al. *JAMA Network Open.*

**Variable**	**Control group** mean (SD)	**Intervention** mean (SD)	**Mean difference*** **(95% CI)**
Pain			
Baseline	17.3 (26.3)	21.9 (28.0)	-
3 months	24.4 (26.2)	25.2 (26.3)	-0.8 [8.7 to -10.2]
6 months	26.7 (28.6)	27.1 (29.3)	-1.5 [8.6 to -11.6]
12 months	22.8 (27.8)	24.4 (25.0)	-2.8 [5.7 to -11.4]
Dyspnoea			
Baseline	22.8 (26.8)	17.0 (20.0)	-
3 months	37.7 (29.7)	31.9 (29.3)	-4.2 [6.5 to -14.9]
6 months	35.3 (31.2)	26.4 (21.3)	-4.8 [5.0 to -14.6]
12 months	34.0 (31.5)	31.9 (28.4)	1.2 [12.3 to -9.9]
Insomnia			
Baseline	31.6 (33.6)	35.7 (30.1)	-
3 months	31.5 (30.0)	29.6 (32.0)	-3.2 [8.1 to -14.6]
6 months	28.7 (31.6)	32.6 (31.3)	0.8 [11.2 to -9.6]
12 months	29.9 (32.1)	34.1 (36.3)	1.0 [12.8 to -10.8]
Appetite loss			
Baseline	16.4 (29.0)	20.2 (31.6)	-
3 months	24.7 (29.8)	16.3 (25.2)	-10.7 [-0.7 to -20.7]
6 months	15.3 (28.7)	16.3 (26.6)	-1.4 [8.7 to -11.5]
12 months	14.3 (21.5)	20.0 (32.1)	1.3 [11.1 to -8.4]
Constipation			
Baseline	18.1 (25.3)	9.9 (19.9)	-
3 months	17.9 (27.3)	14.8 (24.2)	-1.7 [8.7 to -12.0]
6 months	13.3 (20.2)	16.3 (26.6)	5.4 [14.0 to -3.2]
12 months	8.8 (20.2)	10.4 (22.3)	3.8 [11.6 to -4.0]
Diarrhea			
Baseline	7.0 (16.4)	5.8 (15.6)	-
3 months	10.1 (21.3)	5.2 (14.1)	-4.5 [1.8 to -10.8]
6 months	8.7 (22.1)	17.8 (27.6)	12.1 [2.9-21.4]
12 months	5.4 (15.7)	5.2 (14.1)	-0.1 [5.9 to -6.0]
Financial difficulties			
Baseline	16.4 (26.8)	19.9 (25.9)	-
3 months	17.9 (25.7)	17.0 (26.2)	-3.9 [4.5 to -12.4]
6 months	18.7 (30.2)	22.5 (25.9)	-1.3 [6.8 to -9.5]
12 months	11.6 (24.1)	14.1 (25.1)	-0.1 [7.8 to -8.1]
LC13 scales			
Dyspnoea			
Baseline	20.1 (20.7)	18.2 (16.9)	-
3 months	31.1 (24.6)	28.1 (21.3)	-3.2 [5.2 to -11.7]
6 months	29.8 (24.2)	27.0 (19.6)	-2.8 [5.7 to -11.3]
12 months	26.5 (23.3)	23.7 (23.2)	-3.3 [5.7 to -12.3]

© 2024 Granger CL et al. *JAMA Network Open.*

**Variable**	**Control group** mean (SD)	**Intervention** mean (SD)	**Mean difference*** **(95% CI)**
Coughing			
Baseline	35.1 (23.9)	33.9 (20.4)	-
3 months	38.9 (28.8)	39.4 (26.2)	-0.1 [10.5 to -10.6]
6 months	34.0 (21.8)	31.8 (24.1)	-5.0 [3.9 to -13.9]
12 months	27.9 (26.7)	33.3 (23.6)	5.3 [15.0 to -4.4]
Haemoptysis			
Baseline	2.3 (8.6)	3.5 (12.1)	-
3 months	1.2 (6.4)	1.5 (6.9)	-0.1 [3.1 to -3.3]
6 months	0.0 (0.0)	0.8 (5.1)	0.7 [3.1 to -1.6]
12 months	0.0 (0.0)	0.0 (0.0)	0.0 [0.0 to -0.0]
Sore mouth			
Baseline	4.1 (14.2)	2.9 (9.5)	-
3 months	6.2 (17.2)	3.0 (9.6)	-3.0 [2.5 to -8.5]
6 months	8.0 (18.5)	8.5 (26.3)	0.9 [9.9 to -8.2]
12 months	4.8 (15.2)	1.5 (6.9)	-3.0 [1.8 to -7.8]
Dysphagia			
Baseline	1.8 (7.5)	3.5 (10.3)	-
3 months	10.5 (24.1)	6.7 (16.8)	-4.1 [4.1 to -12.3]
6 months	4.7 (15.1)	2.3 (8.6)	-2.6 [2.4 to -7.5]
12 months	4.8 (15.2)	4.4 (13.5)	-0.6 [5.2 to -6.4]
Peripheral neuropathy			
Baseline	15.5 (25.4)	12.9 (23.4)	-
3 months	8.6 (19.6)	13.3 (21.8)	5.3 [12.6 to -1.9]
6 months	22.7 (30.4)	19.4 (27.4)	-2.4 [8.7 to -13.5]
12 months	19.0 (26.4)	17.0 (29.0)	-3.2 [6.5 to -13.0]
Alopecia			
Baseline	9.4 (20.7)	5.8 (14.3)	-
3 months	11.7 (23.5)	8.1 (21.5)	-3.6 [5.1 to -12.2]
6 months	18.7 (30.2)	14.7 (28.5)	-3.3 [7.8 to -14.4]
12 months	11.6 (26.0)	7.4 (20.0)	-2.5 [6.4 to -11.4]
Pain in chest			
Baseline	7.6 (16.7)	13.5 (20.8)	-
3 months	12.3 (19.7)	19.3 (25.1)	4.4 [13.1 to -4.2]
6 months	7.3 (18.2)	14.0 (22.1)	3.7 [11.0 to -3.6]
12 months	10.9 (21.9)	8.9 (16.5)	-3.1 [4.1 to -10.4]
Pain in arm or shoulder			
Baseline	18.1 (26.8)	14.6 (23.6)	-
3 months	17.3 (25.7)	17.8 (23.1)	1.7 [10.7 to -7.2]
6 months	24.7 (34.2)	18.6 (23.3)	-3.6 [7.6 to -14.8]
12 months	22.4 (32.9)	24.4 (33.6)	3.1 [15.3 to -9.1]

© 2024 Granger CL et al. *JAMA Network Open.*

Variable	Control group mean (SD)	Intervention mean (SD)	Mean difference* (95% CI)
Pain in other parts			
Baseline	29.8 (31.9)	28.7 (30.5)	-
3 months	24.1 (29.3)	35.6 (34.4)	12.0 [0.5-23.5]
6 months	36.0 (34.2)	38.0 (28.7)	1.7 [12.4 to -9.1]
12 months	28.6 (31.9)	39.3 (30.4)	8.6 [20.2 to -2.9]
**Other symptom scales**			
Global brief fatigue inventory score[2]			
Baseline	3.3 (2.5)	3.0 (2.4)	-
3 months	3.8 (2.7)	3.2 (2.6)	-0.6 [0.4 to -1.6]
6 months	3.1 (2.6)	3.2 (2.6)	0.1 [1.1 to -0.8]
Sleep disturbance, T-score[3]			
Baseline	50.3 (10.0)	53.9 (10.3)	-
3 months	51.5 (11.9)	50.3 (9.3)	-2.9 [0.8 to -6.6]
6 months	51.6 (10.8)	51.9 (8.2)	-1.6 [1.7 to -4.9]
Distress thermometer[4]			
Baseline	3.7 (3.1)	4.6 (2.9)	-
3 months	2.7 (2.7)	2.7 (2.6)	-0.4 [0.6 to -1.4]
6 months	2.9 (2.8)	2.7 (2.9)	-0.7 [0.2 to -1.7]
Financial toxicity score[5]			
Baseline	21.6 (5.8)	21.8 (6.5)	-
3 months	20.2 (7.0)	22.3 (7.3)	1.8 [4.6 to -0.9]
6 months	20.8 (7.0)	21.0 (6.5)	0.2 [2.9 to -2.5]

Abbreviations: EORTC QLQ-c30, European Organization for the Research and Treatment of Cancer Quality of Life Questionnaire; LC-13, lung cancer sub-scale; QoL, quality of life. *Footnotes:* [1]Lower scores on the global health status and functional scales indicate worse health/function; higher scores on the symptom scales/items indicate worse symptoms; [2] score 0 to 10 with higher scores indicating worse fatigue/interference; [3] higher scores indicate more disturbed sleep; [4] score 0 to 10 with higher scores indicating higher distress; [5] score 0 to 44 with lower scores indicate worse financial wellbeing.

* The model estimates the mean difference between intervention and control from a common baseline so that any change over time between the groups is not due to any difference in baseline scores. Differences use control as the reference group.

© 2024 Granger CL et al. *JAMA Network Open.*

eTable 3. Summary of Secondary Outcome (IPAQ-SF Categorical Outcome for Activity Level)

**IPAQ-SF – categorical**	**Control group n (%)**			**Intervention group n (%)**		
	**Baseline**	**3 months**	**6 months**	**Baseline**	**3 months**	**6 months**
Low	24 (41.4%)	23 (41.8%)	22 (44.0%)	33 (57.9%)	17 (37.8%)	15 (34.0%)
Moderate	33 (56.9%)	29 (52.7%)	26 (52.0%)	20 (35.1%)	24 (53.3%)	23 (52.3%)
High	1 (1.7%)	3 (5.5%)	2 (4.0%)	4 (7.0%)	4 (8.3%)	6 (13.6%)
Missing (count only)	0	3	8	1	13	14

Abbreviations: IPAQ, International Physical Activity Questionnaire short form.

© 2024 Granger CL et al. *JAMA Network Open.*

eFigure. Mean Objectively Measured Physical Function (Short Physical Performance Battery) Over Time

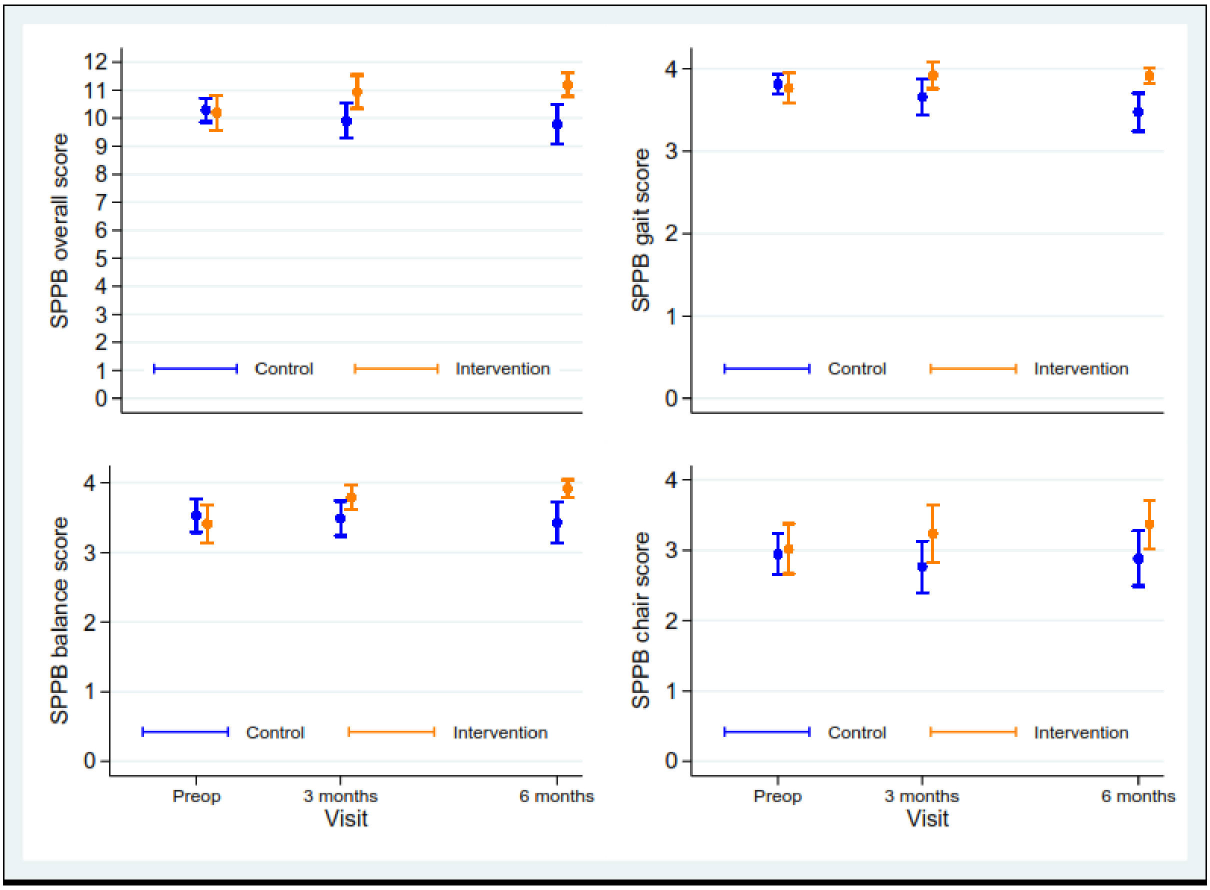

Mean Short Physical Performance Battery (SPPB) scores at baseline, 3- and 6-months follow-up in both intervention and control groups. Error bars represent 95 percent confidence intervals.

eReferences.

1. Hoffmann T, Glasziou P, Boutron I, et al. Better reporting of interventions: template for intervention description and replication (TIDieR) checklist and guide. *BMJ.* 2014;348.
2. Gale J, Skouteris H. *Health coaching: facilitating health behaviour change for chronic condition prevention and self-management. In: Caltabiano M, Ricciardelli L, editors. Handbook of applied topics in health psychology.* Hoboken (NJ): Wiley-Blackwell; 2013.
3. Schmitz K, Courneya K, Matthews C, et al. ACSM roundtable on exercise guidelines for cancer survivors. *Medicine and science in sports and exercise.* 2010;42(7):1409-1426.
4. Sasso J, Eves N, Christensen J, Koelwyn G, Scott J, Jones L. A framework for prescription in exercise-oncology research. *J Cachexia Sarcopenia Muscle.* 2015;6(2):115-124.
5. Borg G. Psychophysical bases for perceived exertion. *Medicine and science in sports and exercise.* 1982;14(5):377-381.
6. Aaronson N, Ahmedzai S, Bergman B, et al. The European Organization for Research and Treatment of Cancer QLQ-C30: a quality-of-life instrument for use in international clinical trials in oncology. *J Natl Cancer Inst.* 1993;85(5):365-376.
7. Bergman B, Aaronson N, Ahmedzai S, Kaasa S, Sullivan M. The EORTC QLQ-LC13: a modular supplement to the EORTC Core Quality of Life Questionnaire (QLQ-C30) for use in lung cancer clinical trials. EORTC Study Group on Quality of Life. *Eur J Cancer.* 1994;30A(5):635-642.
8. Giesinger JM, Kuijpers W, Young T, et al. Thresholds for clinical importance for four key domains of the EORTC QLQ-C30: physical functioning, emotional functioning, fatigue and pain. *Health Qual Life Outcomes.* 2016;14(1):87.
9. Guralnik JM, Simonsick EM, Ferrucci L, et al. A short physical performance battery assessing lower extremity function: association with self-reported disability and prediction of mortality and nursing home admission. *Journal of gerontology.* 1994;49(2):M85-94.
10. Stoffels AA, De Brandt J, Meys R, et al. Short Physical Performance Battery: Response to Pulmonary Rehabilitation and Minimal Important Difference Estimates in Patients With

© 2024 Granger CL et al. *JAMA Network Open.*

Chronic Obstructive Pulmonary Disease. *Archives of physical medicine and rehabilitation.* 2021;102(12):2377-2384.e2375.

11. Holland AE, Spruit MA, Troosters T, et al. An official European Respiratory Society/American Thoracic Society technical standard: field walking tests in chronic respiratory disease. *Eur Respir J.* 2014;44(6):1428-1446.
12. Granger C, Holland A, Gordon I, Denehy L. Minimal important difference of the 6-minute walk distance in lung cancer. *Chron Resp Dis.* 2015;12(2):146.
13. Mentiplay BF, Perraton LG, Bower KJ, et al. Assessment of Lower Limb Muscle Strength and Power Using Hand-Held and Fixed Dynamometry: A Reliability and Validity Study. *PLOS ONE.* 2015;10(10):e0140822.
14. Craig CL, Marshall AL, Sjostrom M, et al. International physical activity questionnaire: 12-country reliability and validity. *Medicine and science in sports and exercise.* 2003;35(8):1381-1395.
15. Rogers, Courneya KS, Verhulst., Markwell., Lanzotti., Shah. Exercise barrier and task self-efficacy in breast cancer patients during treatment. *Supportive care in cancer : official journal of the Multinational Association of Supportive Care in Cancer.* 2006;14:84-90.
16. McAuley E, Mihalko S. *Advances in sports and exercise psychology measurement. .* Morgantown, WV: Fitness Information Technology, Inc.; 1998.
17. Mendoza TR, Wang XS, Cleeland CS, et al. The rapid assessment of fatigue severity in cancer patients: use of the Brief Fatigue Inventory. *Cancer.* 1999;85(5):1186-1196.
18. Yu L, Buysse DJ, Germain A, et al. Development of short forms from the PROMIS sleep disturbance and Sleep-Related Impairment item banks. *Behavioral sleep medicine.* 2011;10(1):6-24.
19. Roth A, Kornblith A, Batel-Copel L, Peabody E, Scher H, Holland J. Rapid screening for psychologic distress in men with prostate carcinoma: a pilot study. *Cancer.* 1998;82(10):1904-1908.

© 2024 Granger CL et al. *JAMA Network Open.*

20. de Souza JA, Yap BJ, Wroblewski K, et al. Measuring financial toxicity as a clinically relevant patient-reported outcome: The validation of the COmprehensive Score for financial Toxicity (COST). *Cancer.* 2017;123(3):476-484.

© 2024 Granger CL et al. *JAMA Network Open.*
