# Supplementary material for: Home-Based Exercise and Self-Management After Lung Cancer Resection: A Randomized Clinical Trial
Source: JAMA Netw Open. 2024 Dec 2;7(12):e2447325. doi: 10.1001/jamanetworkopen.2024.47325 (PMC11612835; doi:10.1001/jamanetworkopen.2024.47325)
Supplement: Supplement 3. — Data Sharing Statement [file jamanetwopen-e2447325-s003.pdf]

Data Sharing Statement

Granger. Home-Based Exercise and Self-Management After Lung Cancer Resection. *JAMA Netw Open*. Published December 02, 2024. doi:10.1001/jamanetworkopen.2024.47325

Data
Additional Information: Australian and New Zealand Clinical Trials Registry ANZCTR https://anzctr.org.au/ Identifier: ACTRN12617001283369.
Data available: Yes
Data types: Deidentified participant data, Data dictionary
How to access data: Available from corresponding author via email request to catherine.granger@unimelb.edu.au
When available: With publication

Supporting Documents
Document types: Statistical/analytic code, Informed consent form, Other (please specify)
Additional Information: Also available - patient educational material and data collection forms
How to access documents: Available from corresponding author via email request to catherine.granger@unimelb.edu.au
When available: With publication

Additional Information
Who can access the data: Researchers whose proposed use of the data has been approved.
Types of analyses: For specified research purpose.
Mechanisms of data availability: With investigator support, after approval of a proposal and appropriate ethical approval, with a signed data access agreement.
Any additional restrictions: Nil
